# Cartilaginous Epiphyses in Extant Archosaurs and Their Implications for Reconstructing Limb Function in Dinosaurs

**DOI:** 10.1371/journal.pone.0013120

**Published:** 2010-09-30

**Authors:** Casey M. Holliday, Ryan C. Ridgely, Jayc C. Sedlmayr, Lawrence M. Witmer

**Affiliations:** 1 Department of Pathology and Anatomical Sciences, School of Medicine, University of Missouri, Columbia, Missouri, United States of America; 2 Department of Biomedical Sciences, College of Osteopathic Medicine, Ohio University, Athens, Ohio, United States of America; 3 Department of Cell Biology and Anatomy, School of Medicine, Louisiana State University - New Orleans, New Orleans, Louisiana, United States of America; Raymond M. Alf Museum of Paleontology, United States of America

## Abstract

Extinct archosaurs, including many non-avian dinosaurs, exhibit relatively simply shaped condylar regions in their appendicular bones, suggesting potentially large amounts of unpreserved epiphyseal (articular) cartilage. This “lost anatomy” is often underappreciated such that the ends of bones are typically considered to be the joint surfaces, potentially having a major impact on functional interpretation. Extant alligators and birds were used to establish an objective basis for inferences about cartilaginous articular structures in such extinct archosaur clades as non-avian dinosaurs. Limb elements of alligators, ostriches, and other birds were dissected, disarticulated, and defleshed. Lengths and condylar shapes of elements with intact epiphyses were measured. Limbs were subsequently completely skeletonized and the measurements repeated. Removal of cartilaginous condylar regions resulted in statistically significant changes in element length and condylar breadth. Moreover, there was marked loss of those cartilaginous structures responsible for joint architecture and congruence. Compared to alligators, birds showed less dramatic, but still significant changes. Condylar morphologies of dinosaur limb bones suggest that most non-coelurosaurian clades possessed large cartilaginous epiphyses that relied on the maintenance of vascular channels that are otherwise eliminated early in ontogeny in smaller-bodied tetrapods. A sensitivity analysis using cartilage correction factors (CCFs) obtained from extant taxa indicates that whereas the presence of cartilaginous epiphyses only moderately increases estimates of dinosaur height and speed, it has important implications for our ability to infer joint morphology, posture, and the complicated functional movements in the limbs of many extinct archosaurs. Evidence suggests that the sizes of sauropod epiphyseal cartilages surpassed those of alligators, which account for at least 10% of hindlimb length. These data suggest that large cartilaginous epiphyses were widely distributed among non-avian archosaurs and must be considered when making inferences about locomotor functional morphology in fossil taxa.

## Introduction

Most vertebrate movement is dependent on articulations that join bony elements together, and these joints are generally located at the ends of long bones. Beyond permitting movement, the ends of bones contribute to other functions as well, including the lubrication of the joint [1], intracapsular ligament attachment [2], force transmission of locomotor impact [3], and bone growth [4]. However, the ends of bones are not completely osseous, but rather have variable amounts of cartilage. In the process of skeletonization, whether in nature or the lab, these terminal cartilaginous caps are lost. Thus, the dried bony elements are not the same functional elements used by an animal, but rather just the mineralized portion. Whereas the extent of the cartilaginous cap can be directly assessed among extant animals, decomposition, fossilization, and other taphonomic processes strip away this functional information, and paleontologists are left to hypothesize the limb and joint anatomy of extinct taxa without what could be a substantial part of the functional limb of the organism.

**Figure 1:**
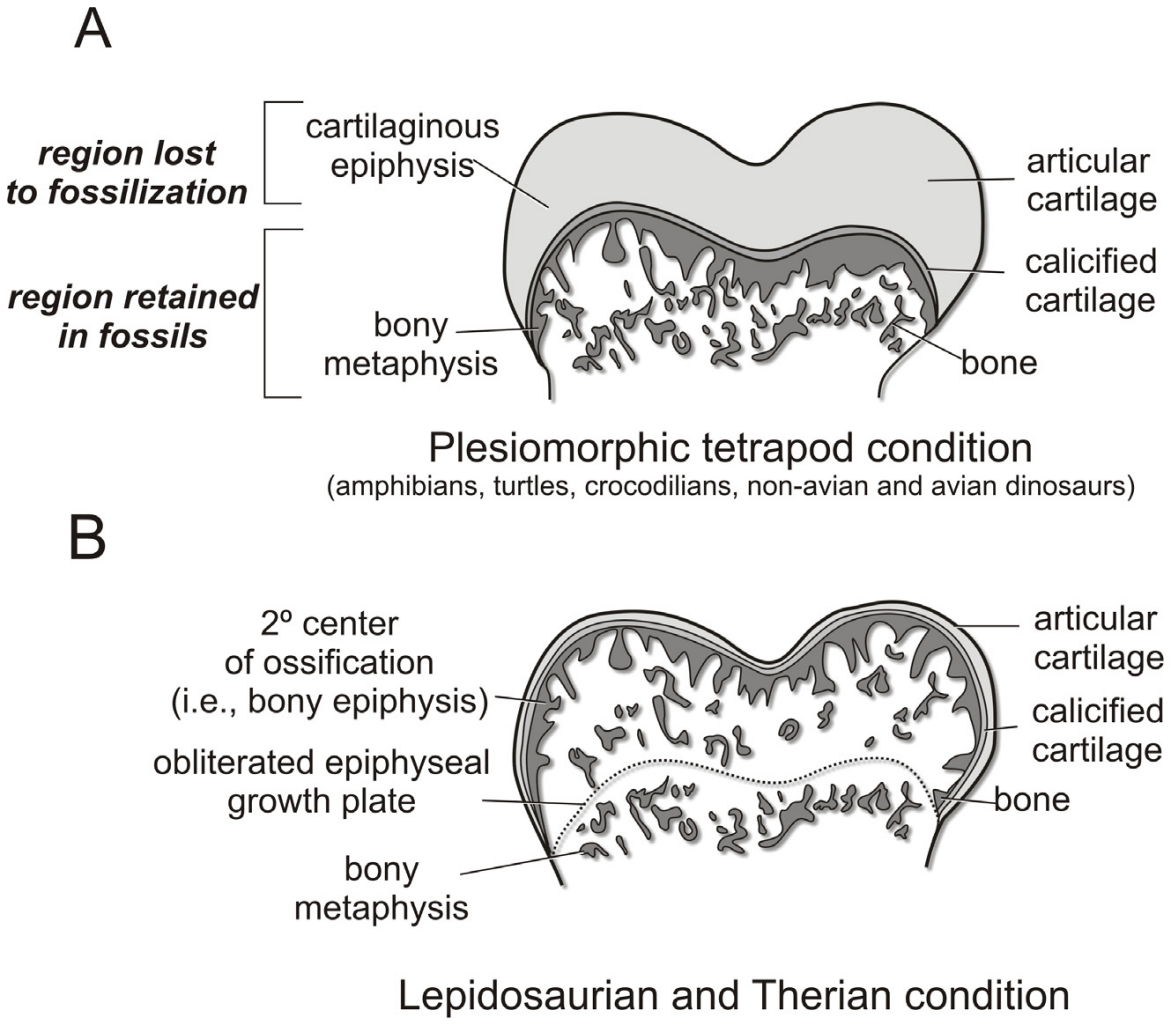
Different extant tetrapod clades retain variable amounts of epiphyseal cartilage. **A**, Plesiomorphic tetrapod condition, also characteristic of turtles, crocodylians, birds, and likely non-avian dinosaurs. **B**, Lepidosaurian and therian condition. Modified from Haines [9].

Among extant animals, epiphyseal cartilage has been the focus of anatomical descriptions [5]–[11], proposed mechanical and evolutionary models [3], phylogenetic interpretations [12], [13], and numerous biomedical studies [14]–[15]. Different clades of amniotes vary in the extent to which they retain or ossify their epiphyses. During early limb development in amphibians, turtles, crocodylians, birds, and hypothetically non-avian dinosaurs [16]–[19], a cartilage cone develops within the metaphysis that is connected to the epiphyseal cartilage [3], [9], [20] (Fig. 1A). Endochondral ossification continues just deep to the epiphyseal region, eventually engulfing and obliterating the cartilage cone, leaving the terminal cartilaginous cap that comprises the epiphysis. In birds, ossification proceeds much as in crocodylians and turtles, but differs in that most of the epiphysis is eventually assimilated into endochondral bone, leaving just the relatively thin hyaline cartilage of the articular surface. Secondary centers of ossification (i.e., bony epiphyses) are absent in turtles, crocodylians, and birds [12] (Fig. 1B). On the other hand, in mammals and most lizards, secondary centers of ossification develop [20], leaving only a relatively thin layer of hyaline articular cartilage on the terminal ends of the element. Endochondral ossification continues as chondrocytes hypertrophy, proliferate, and form a growth plate between the metaphyseal bone and epiphyseal cartilage [19] [21]. This scaffold of cartilage cells forms a thin lamina of calcified cartilage that persists as an evenly curved surface on the end of the bone [20]. Despite our understanding of skeletal tissue biology, few studies have attempted to quantify how much of an epiphyseal cartilaginous cap is present, particularly in reptiles [22]–[24].

Some of the earliest published inferences of dinosaur behavior were based on the seemingly poorly ossified, “unfinished” ends of sauropod limb bones. For example, Owen [25], Cope [26], [27], Marsh [28], [29], Osborn [30], Hatcher [31], and Hay [32]regarded “the abundance of cartilage around all the limb joints…[as] positive evidence that the limbs were not continuously subjected to the hard impact of the enormous weight of the body by motion on land.” Thus was born the notion of aquatic or amphibious sauropods that held currency until the 1970s [33]. Cartilaginous epiphyseal tissues have been identified at the histological level on the distal metaphyses of some sauropod specimens (*Cetiosaurus*; [34]). However, the extent to which the epiphyseal cartilaginous caps covered the ends of long bones remains unexplained [35]. More recently, although the presence of epiphyses on dinosaur limb bones has sometimes been noted [36]–[40], dinosaur skeletons typically are reconstructed with the ends of the limb bones directly contacting each other, as if there never were a cartilaginous cap [41]–[47]. Moreover, several sophisticated functional and biomechanical analyses gave little or no specific attention to any role potentially played by the cartilages [42], [43], [48]–[52], whereas others explicitly included estimates of missing epiphyses while framing functional questions [11], [53]–[55]. Although Thulborn [56] introduced a 9% correction factor to account for missing epiphyses in some dinosaur taxa and Hutchinson et al., [57] incorporated correction factors based on a crocodylian specimen in their analysis of *Tyrannosaurus* running, to date, there remains no published objective basis for just how much cartilage needs to be reconstructed, and potential problems associated with ignoring the epiphyseal cartilage have not been identified. Inferences of epiphysis size may simply affect our estimations of dinosaur size and height. On the other hand, epiphyseal considerations may greatly influence our interpretation of joint function and posture.

Adult crocodylian limb bones have notoriously poorly-defined bony epiphyses making reconstructions of joint articulations based solely on bones challenging. Limb bones of birds, on the other hand, have more well-defined structures resulting in more congruent bony articulations. These casual observations led us to test the hypothesis that there are significant changes in dimensions of limb elements before and after the removal of epiphyseal cartilages. Because epiphyseal cartilages are in many cases developmentally important sites of bone elongation, we expected cartilage thickness to decrease relative to body size (a proxy for age), thus exhibiting negative allometry. We then used these data on extant taxa to infer how much cartilage dinosaurs and other extinct archosaurs may have had on the ends of their bones, to assess how significant an impact it may have on interpreting biology, and to offer new functional and evolutionary insights in this primary investigation of the gross anatomy of archosaurian epiphyseal cartilages. Finally, two similar, recent studies into the epiphyseal cartilages of archosaurs, both of which were inspired by this article's original conference abstract [58], have also demonstrated significant changes in long bone length and shape after skeletonization [11], [59] and this paper complements their findings.

## Materials and Methods

### Ethics Statement

All research was conducted on salvaged animal specimens and no approval from Ohio University Institutional Animal Care and Use Committee was necessary.

**Figure 2:**
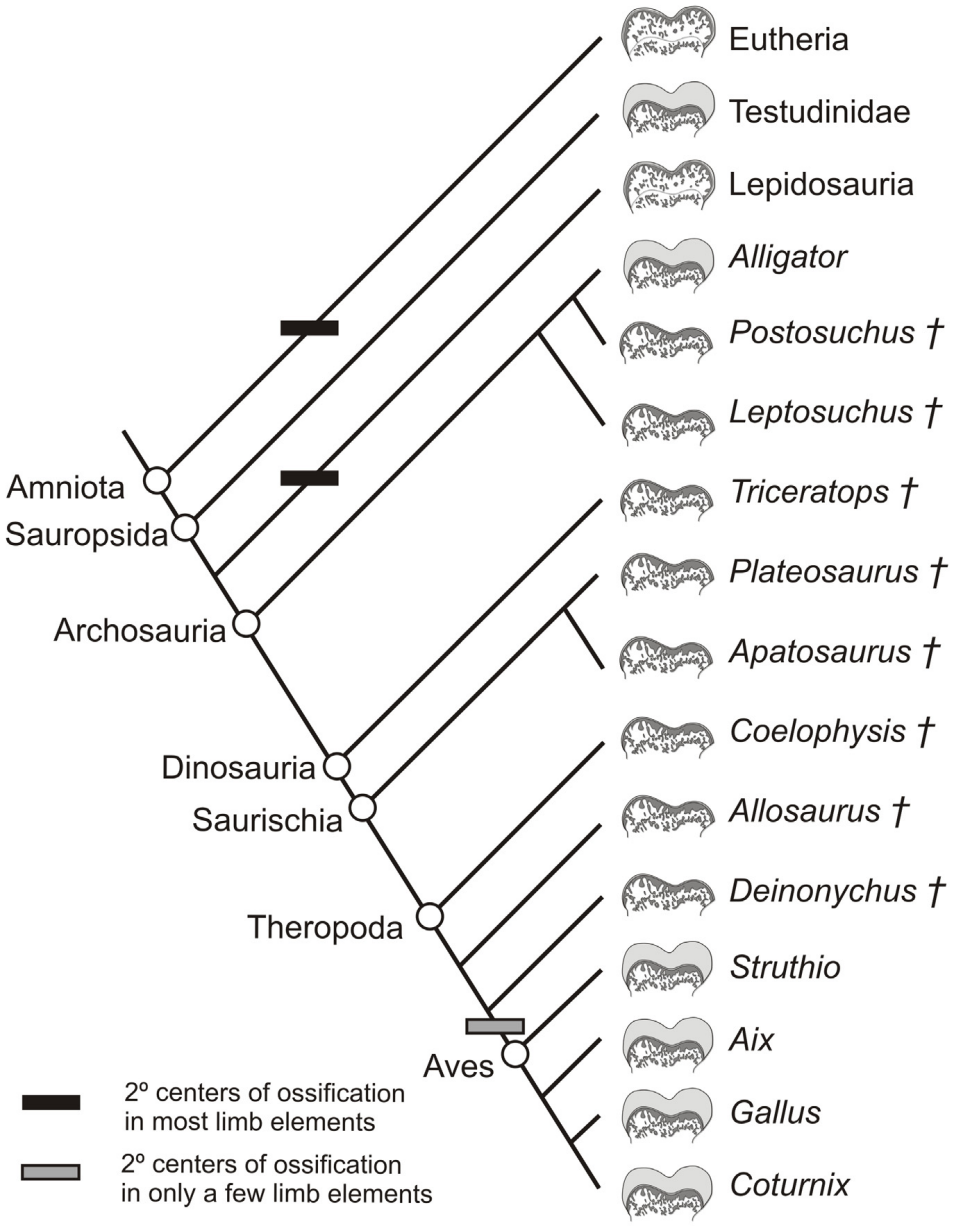
Phylogenetic framework of extant and extinct archosaur taxa examined in this study including characteristic epiphyseal morphology. Phylogenetic relationships based on Brochu [71]. †, extinct taxa.

We employed the extant phylogenetic bracket (EPB) approach [60] to investigate the soft-tissue relations of articular structures (e.g., cartilages, joint capsular ligaments, muscles) and their bony signatures in extant and fossil archosaurs (Fig. 2). By investigating the epiphyseal cartilages of the two closest extant relatives of non-avian dinosaurs (i.e., crocodylians and birds), more accurate inferences can be made regarding the amount of epiphyseal cartilage of dinosaurs. The limbs of living archosaurian taxa were investigated to discover (1) whether there is a significant amount of limb epiphyseal cartilage present, (2) which limb elements show the most change after skeletonization, and (3) how crocodylian and avian epiphyses differ. These anatomical observations of the living taxa will constrain inferences regarding the soft-tissue reconstruction and limb function of the extinct clades.

Specimens used were typical representatives of extant archosaurs. Crocodylia was represented by American alligator (*Alligator mississippiensis*, Ohio University Vertebrate Collections [OUVC] 9401–9415). Aves was represented primarily by ostrich (*Struthio camelus*, OUVC 9422–9439), supplemented with chicken (*Gallus gallus*, OUVC 9419–9420), Japanese quail (*Coturnix japonicus*, OUVC 9416–9418), and wood duck (*Aix sponsa*, OUVC 9421). The limb lengths of representative non-avian dinosaurs and published hindlimb postures of *Struthio* and *Tyrannosaurus rex* [52] were used in a quantitative sensitivity analysis to test the affect that epiphyseal cartilage has on estimations of height, posture, and walking and running speed. Finally, the femora of several basal archosaurs (*Leptosuchus* and *Postosuchus*) and non-avian dinosaurs (*Plateosaurus*, *Coelophysis*, *Triceratops*, *Apatosaurus*, *Allosaurus*, and *Deinonychus*) were studied to qualitatively compare limb morphology with the extant archosaurs.

### Preparation and measurement

The alligator sample consisted of 15 specimens obtained from the Rockefeller Wildlife Refuge (Grand Chenier, LA) ranging in size from about 0.5 m to 2.5 m total length. Ostriches were obtained from a commercial processing center, and all individuals were of roughly equivalent size. The twenty specimens of ostrich included two whole, intact individuals, 12 sets of hindlimbs and humeri, and six individual femora. Two different age classes of ostriches were distinguishable, a subadult and adult class, the former having unfused cnemial ossification centers on the proximal tibia and a very rugose and unfinished condylar bone texture. The limbs were carefully disarticulated and defleshed manually, leaving the epiphyses and articular cartilage intact. For alligators, the following limb elements were used: humerus, ulna, radius, femur, tibia, and fibula. Because of the large number of partial ostrich specimens, only the femora, tibiae, fibulae, and humeri of birds were used. Fibular lengths were not measured in avian taxa because the fibula tapers distally to a splint along the tibiotarsus.

**Figure 3:**
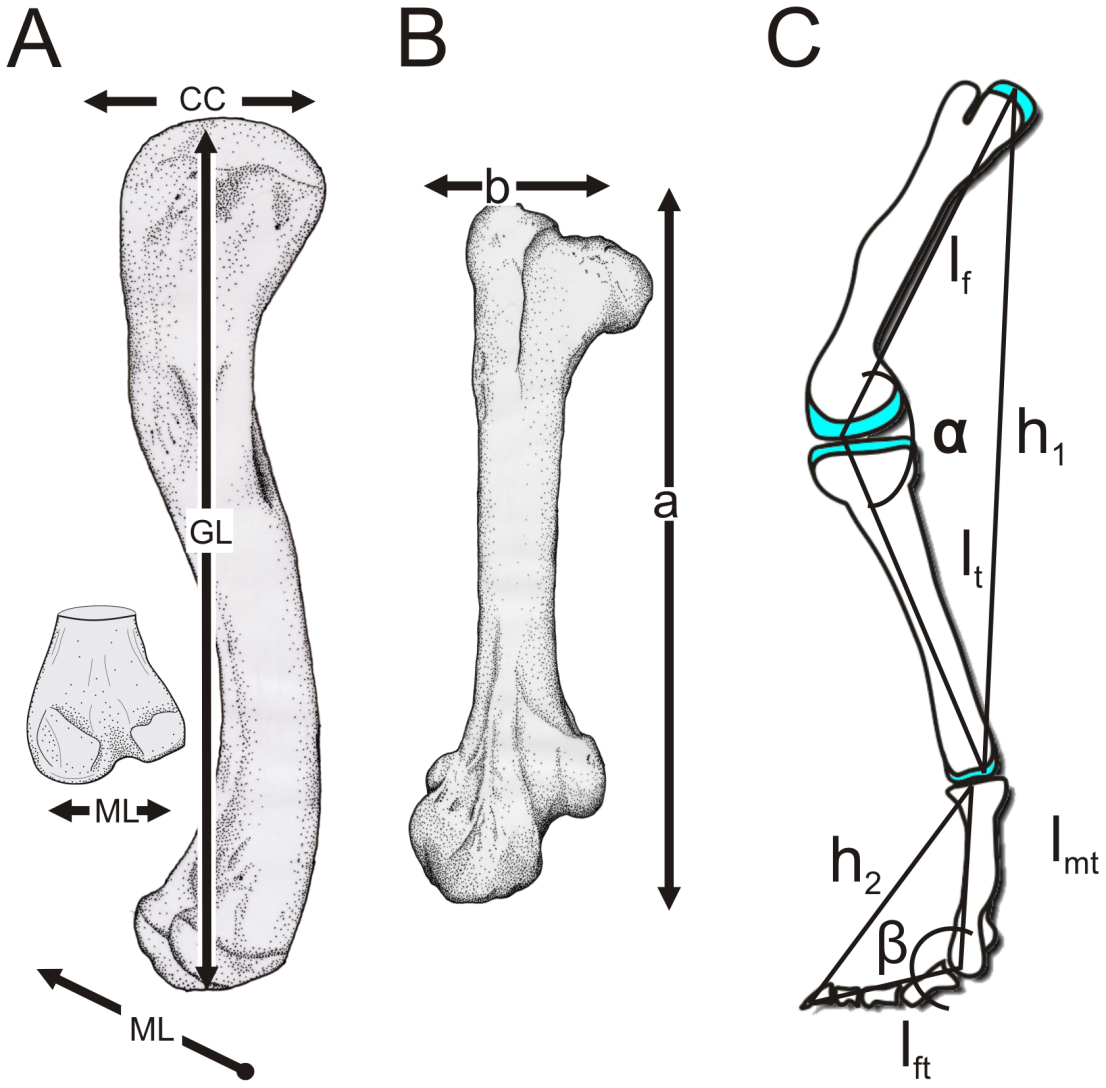
Measurements conducted for quantitative analyses. **A**, measurements indicated on left femur of *Alligator mississippiensis* (American alligator) in medial view: CC, craniocaudal; GL, greatest length; ML, mediolateral. **B**, GL measurement for avian specimens indicated on left femur of *Struthio camelus* (ostrich) in cranial view. GL in birds equals the length from the distal condyles to trochanteric shelf (a) plus the length from the same point on the trochanteric shelf to the medial end of the femoral head (b). **C**, Segmental measurements and joint angles used from non-avian dinosaur speed estimates (adapted from Gatesy et al. [55]).

**Figure 4:**
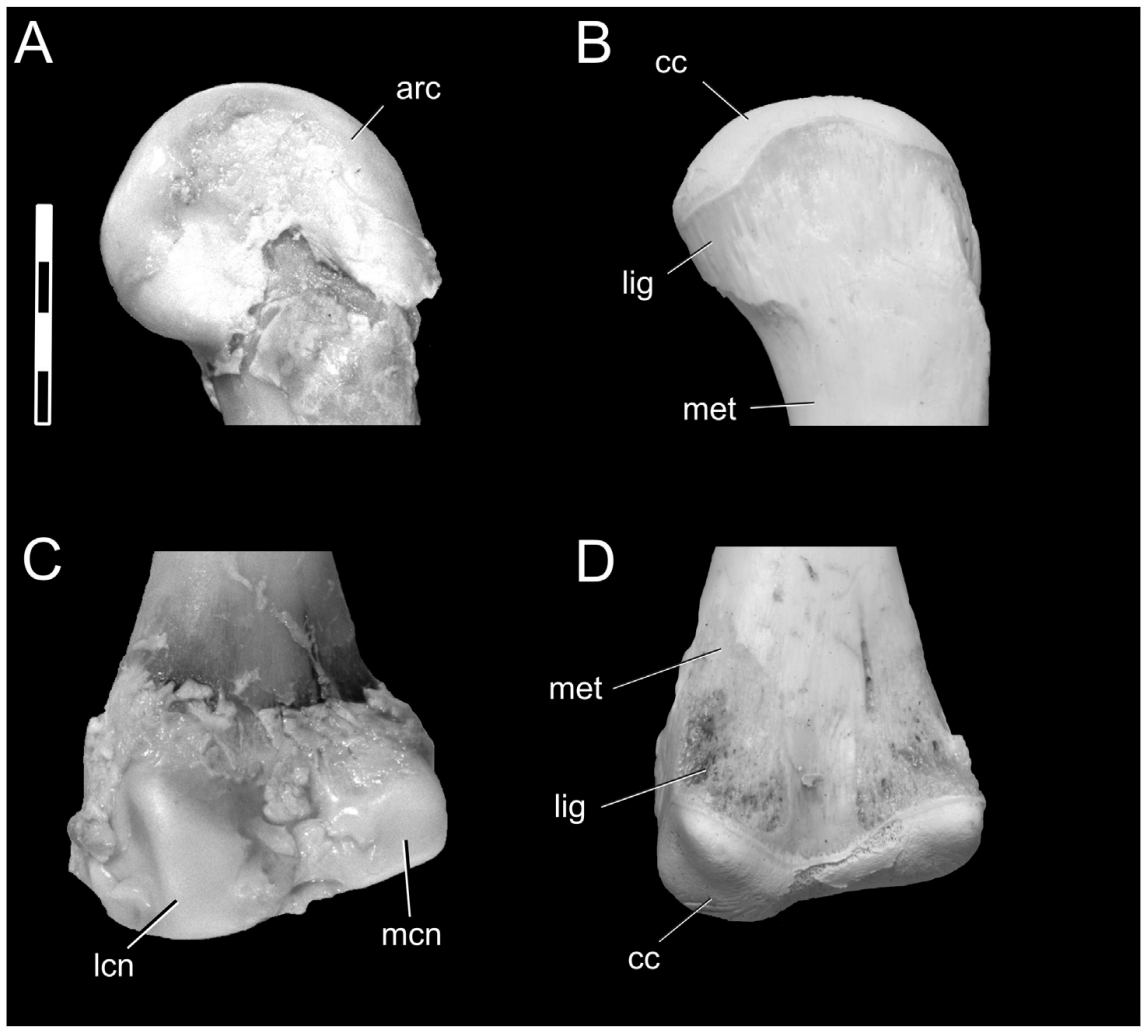
Changes in epiphyses of *Alligator mississippiensis* upon skeletonization. Left femur (OUVC 9407) before (**left**) and after (**right**) skeletonization. **A** and **B**: proximal end, cranial view. C, D: distal end, caudal view. Abbreviations: **ac**, articular cartilage; **cc**, calcified cartilage; **lig**, scar from ligaments and synovial capsule; **lfc**, lateral femoral condyle; **met**, metaphysis; **mfc**, medial femoral condyle. Scale bar increments equal 0.5 cm.

**Figure 5:**
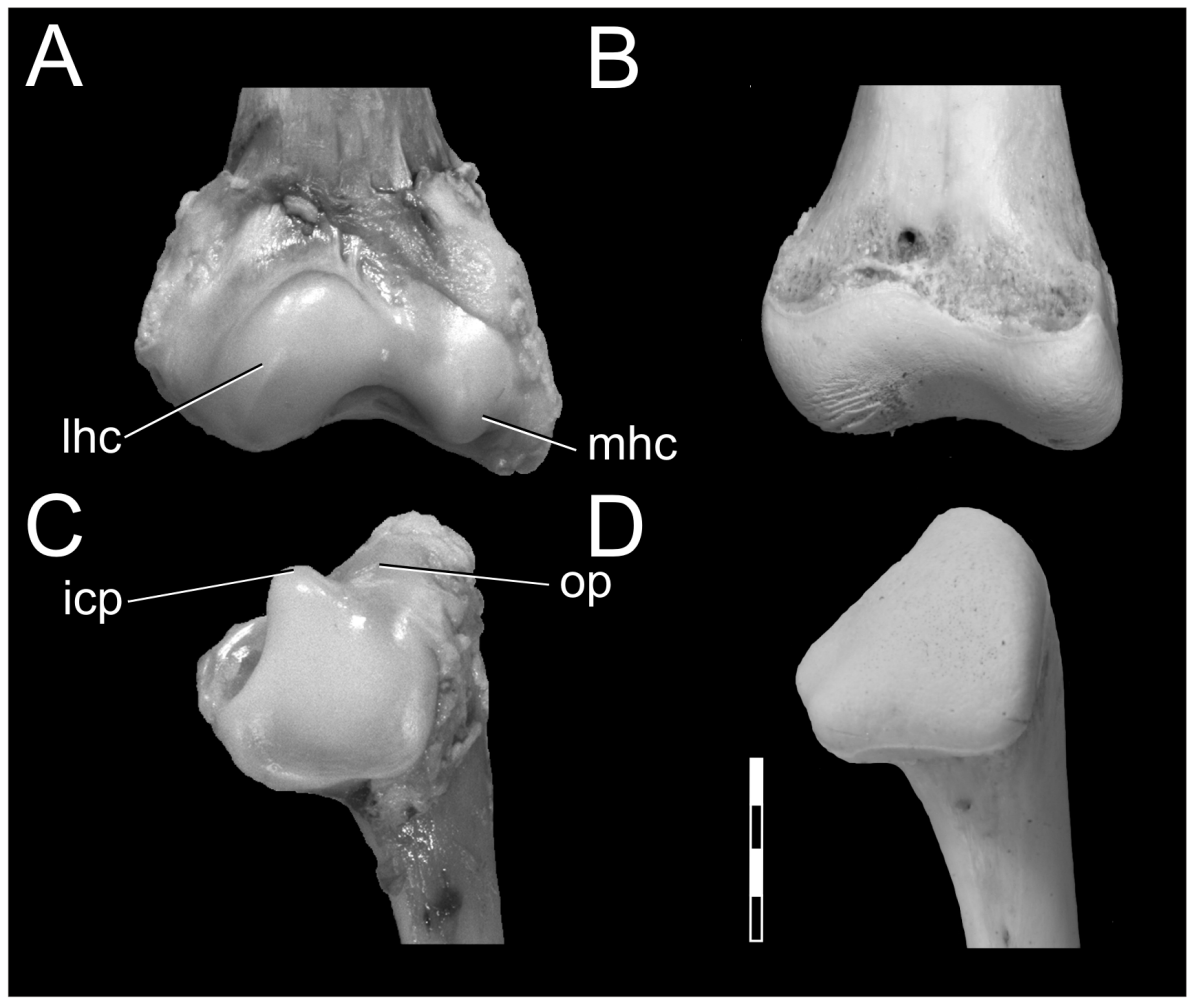
Changes in epiphyses of *Alligator mississippiensis* upon skeletonization. Left distal humerus and proximal ulna (OUVC 9407) before (left) and after (right) skeletonization. **A** and **B**: humerus, distal end, cranial view. **C**, **D**: ulna, proximal end, cranial view. Abbreviations: **icp**, intercotylar process; **lhc**, lateral humeral condyle; **mhc**, medial humeral condyle; **op**, olecranon process. Scale bar increments equal 0.5 cm.

Measurements taken from each limb element included greatest length (GL) and proximal and distal condylar breadths in both craniocaudal (CC) and mediolateral (ML) directions (Figs. 3A; 4, 5). Because of the angulation of the femoral head and neck relative to the shaft in birds, GL measurements differed from those of alligators in that two measurements were used to equal GL: the distance from the distal condyles to the trochanteric shelf plus the distance from the same point on the trochanteric shelf to the medial end of the femoral head (Fig. 3B: GL  = a+b). Three replicates of each measurement were made for each element, and the right and left elements were then averaged to represent one individual. The fleshy element was then skeletonized in a warm water bath with Terg-a-Zyme (Alconox Inc., Jersey City, NJ) biological detergent or macerated by dermestid beetles to remove the soft tissues without affecting the bone. After the bony element had completely dried, the same measurements were repeated to document the amount of change between fleshy and bony element, and hence the amount of epiphyseal cartilage.

### Qualitative Analysis

Qualitative observations regarding soft-tissue structures associated with the articular regions—including surrounding musculature, synovial and fibrous capsules, and ligaments—were documented with dissection and photography to clarify the anatomy of the limb elements and to ensure that the cartilage under study was indeed epiphyseal in nature. To preserve the morphology of the fleshy elements, the limb elements of one alligator specimen (OUVC 9401) were prepared as above, leaving the epiphyses intact. The fleshy limb was then molded in Por-a-Mold (S-333) polyurethane and cast in Por-a-Kast (Synair Co., Chattanooga, TN) to retain size and shape attributes for future comparison with the skeletonized element. Fujiwara et al. [11] also molded and cast their specimens.

### Quantitative analysis

Linear changes between fleshy and bony elements of all taxa were analyzed with paired t-tests (NCSS, Kaysville, UT) using a Bonferroni adjustment (p<0.01) and the percent change from fleshy to bony phase of the each element was calculated to demonstrate how much of the functional dimension was lost to skeletonization (Table 1). Two-sample t-tests of arcsin-transformed percentages were employed to gauge differences between (1) alligators and ostriches and between (2) subadult and adult ostriches (Table 2).

**Table 1:** Mean, standard deviation, and paired t-test results of changes in limb element dimensions, reported as percent change, of all taxa after skeletonization.

	Percent Change in Dimension (Mean ± SD)				
Limb element	GL	Proximal CC	Proximal ML	Distal CC	Distal ML
*Alligator* (n = 15)					
Humerus	7.99±2.84*	12.56±7.65*	26.94±8.25*	11.91±6.04*	22.82±5.55*
Ulna	8.58±2.42*	18.54±6.91*	20.83±6.74*	18.78±4.94*	23.67±10.90*
Radius	8.18±2.32*	17.90±5.70*	17.72±7.28*	22.29±7.13*	21.01±7.92*
Femur	6.29±1.65*	13.26±4.16*	22.83±6.38*	8.80±3.20*	15.46±5.60*
Tibia	4.52±2.67*	14.62±4.64*	15.26±7.02*	12.60±4.61*	15.15±9.74*
Fibula	6.10±1.63*	19.78±7.24*	22.18±11.57*	18.42±5.81*	21.19±7.17*
*Struthio* Adult (n = 8)					
Humerus	2.35±1.10*	9.24±3.49*	11.22±5.79*	7.56±2.24*	9.74±3.59*
Ulna (1)	4.20	9.50	5.50	2.50	1.90
Radius (1)	3.00	3.00	5.00	10.00	7.00
Femur	4.69±1.64*	7.43±2.55*	4.65±3.15*	2.30±1.38*	3.26±1.71*
Tibia	2.07±0.92*	1.95±1.22*	4.03±2.36*	4.03±2.12*	4.11±0.90*
Fibula	nm	13.39±4.46*	7.43±5.72*	nm	nm
*Struthio* Sub-adult (n = 4)					
Humerus	3.72±0.47*	8.04±2.72*	19.71±4.14*	15.75±8.44*	19.69±6.78*
Ulna (1)	8.00	19.00	14.00	13.00	27.00
Radius (1)	3.00	24.00	12.00	9.00	14.00
Femur	6.40±2.25*	26.72±4.13*	18.33±6.79*	5.68±2.10*	5.28±1.31*
Tibia	4.38±0.98*	5.60±1.95*	11.84±1.77*	5.22±3.81*	7.23±3.81*
Fibula	nm	16.02±5.87*	7.47±3.00*	nm	nm
*Coturnix* (n = 3)					
Humerus	0.56±0.23	5.41±3.13	6.34±1.68	10.77±3.84*	5.63±3.99
Ulna	2.36±2.05	0.68±1.18	5.52±1.93	4.40±1.55*	2.43±2.27*
Radius	0.620±0.82	3.99±0.46	2.06±0.69	4.82±0.79	1.43±0.12
Femur	1.00±0.38	4.14±2.22	5.45±2.99	3.36±0.43*	2.88±3.13
Tibia	0.84±0.53	3.16±2.66	0.71±1.24	4.90±1.14*	2.89±0.84*
Fibula	nm	5.99±2.06	4.61±1.61	nm	nm
*Gallus* (n = 2) ‡					
Humerus	3.97±3.51	13.23±1.49	8.47±9.32	12.99±4.69	31.47±10.98
Ulna	3.89±0.94	17.57±5.1	14.18±5.40	9.95±6.43	16.48±16.05
Radius	5.70±5.11	20.47±9.49	3.63±4.46	11.80±1.54	12.59±3.66
Femur	9.54±3.25	30.63±3.90	19.89±3.05	14.90±0.10	6.99±5.50
Tibia	5.47±3.45	14.94±0.45	5.75±1.61	4.16±2.55	17.18±19.11
Fibula	nm	8.89±5.84	13.95±2.16	nm	nm
*Aix* (n = 1) ‡					
Humerus	0.07	1.70	13.41	3.03	1.48
Ulna	0.31	6.78	2.73	0.46	6.60
Radius	0.00	5.70	0.92	0.69	0.92
Femur	0.92	1.66	4.46	1.30	1.59
Tibia	0.11	3.23	3.02	0.91	1.44
Fibula	nm	7.14	2.75	nm	nm

Results of Bonferroni-adjusted paired t-tests as follows:

*, *p*<0.01;

‡, paired t-tests not applicable due to small sample size; nm, not measured. Abbreviations: GL, greatest length; CC, craniocaudal dimension; ML, mediolateral dimension; nm, dimension not measured.

**Table 2:** Results of Bonferroni-adjusted paired t-tests of arcsin-transformed percent changes of archosaur limb element dimensions.

	GL	Proximal CC	Proximal ML	Distal CC	Distal ML
Percent Change Subadult *Struthio* (n = 4) vs. Adult *Struthio* (n = 8)					
Humerus	ns	ns	*	ns	**
Femur	*	*	*	ns	ns
Tibia	ns	ns	*	ns	ns
Percent Change *Alligator* (n = 14) vs. Adult *Struthio* (n = 8)					
Humerus	*	ns	*	*	*
Femur	ns	*	*	*	*
Tibia	ns	*	*	*	*

H_o_: there is no difference between compared elements. Results of t-tests: *, significant (*p*<0.0); ns, not significant. Abbreviations as in Table 1.

Because of the large range of body size among the alligators, it was unclear if the size of the epiphyses of smaller animals was significantly different from those of larger ones. If ontogenetic differences were not apparent, we would be justified in our pooling of the individuals. Studies of other vertebrates have shown that femoral midshaft cross-sectional area calculated from biplanar X-rays is an accurate proxy for body mass [61]–[65]. We measured alligator subperiosteal and endosteal radii using Craftsman needle-nosed calipers on hand-developed Kodak Industrex M X-ray film exposed using a HP Faxitron soft X-ray machine (30 kvp, 2.75 mA, duration  = 180sec, film-to-source distance = 122 cm) and calculated circular cross-sectional area to the nearest 0.01 mm. Scaling relationships between the log_10_ difference between fleshy and bony limb element dimensions and log_10_ femoral midshaft cross sectional area were estimated using model II reduced major axis (RMA) regressions calculated using SYSTAT version 9 (SPSS Inc.; Chicago, IL). Evaluation of allometry was based on whether 95% confidence intervals included slope values expected for isometry, in this case m = 0.50 for length-area relationships [64], [66] (Table 3).

**Table 3:** Absolute change in limb length after epiphysis removal compared to body size in *Alligator*.

Element Dimension	95% confidence interval	r	Trend
Greatest Length			
Femur	0.113–0.329	0.69*	-
Tibia	0.181–0.521	0.34	ns
Fibula	0.181–0.521	0.71*	0
Humerus	0.044–0.348	0.24	ns
Ulna	0.152–0.497	0.63*	-
Radius	0.147–0.473	0.65*	-
Proximal Craniocaudal			
Femur	0.187–0.569	0.68*	0
Tibia	0.121–0.687	0.38	ns
Fibula	0.198–0.687	0.61*	0
Humerus	0.223–0.432	0.87*	-
Ulna	0.114–0.481	0.52	-
Radius	0.140–0.481	0.60*	-
Proximal Mediolateral			
Femur	0.165–0.489	0.69*	-
Tibia	0.089–0.636	0.29	ns
Fibula	0.057–0.776	0.04	ns
Humerus	0.266–1.246	0.49	0
Ulna	0.233–0.853	0.59*	0
Radius	0.112–0.765	0.30	
Distal Craniocaudal			
Femur	0.177–0.756	0.51	0
Tibia	0.370–0.982	0.74*	0
Fibula	0.145–0.771	0.41	ns
Humerus	0.233–0.475	0.87*	-
Ulna	0.044–0.679	0.03	ns
Radius	0.277–0.776	0.71*	0
Distal Mediolateral			
Femur	0.135–0.576	0.52	0
Tibia	0.110–0.686	0.34	ns
Fibula	0.234–0.580	0.77*	0
Humerus	0.024–0.580	0.21	ns
Ulna	0.363–0.760	0.76*	0
Radius	0.237–0.567	0.03	0

RMA regressions of log_10_ difference between element with and without epiphyseal cartilage compared to log_10_ femoral midshaft cross-sectional area. Abbreviations:

*, *p*<0.05;

-, negative allometry (m<0.5); 0, isometry (m = 0.5).

### Applications to fossil taxa

First, select ornithischian and saurischian dinosaur limb lengths were calculated with columnar arrangements, with and without different correction factors. Second, these corrected limb lengths were used to estimate locomotor speeds at Froude numbers (Fr) of 1 and 16 (Table 4). Froude number [calculated by Fr  =  (velocity^2^)/(hip height x *g*), where g  = 9.81 ms^−2^] is the ratio of centripetal to gravitational forces and is a routine means of estimating theoretical forward velocity [67]–[68]. It is expected that at a Froude number near 1, an animal is moving at a slow run. Hutchinson and Garcia [68] used Fr  = 16 to estimate theoretical, highest-speed running in *Tyrannosaurus* (although they regarded such a speed as unlikely), and thus we apply that assumption here as well. Third, theropods likely used more crouched joint postures than the more columnar sauropods and many other taxa. Thus, to better refine speed estimates, we applied the same cartilage correction factors to hip heights calculated from different joint postures [55] in a sample of theropod taxa [69]. CCFs for alligator and ostrich were applied to the femoral and tibial lengths of a variety of small- and large-bodied theropod dinosaurs. Then, different knee and metatarsophalangeal angles for a modestly crouched *Tyrannosaurus* (*T. rex*: 124°, knee; 147°, ankle; fig. 5D in Gatesy et al. [55] and ostrich (*Struthio*: 109°, knee; 142°, ankle; fig 5F in Gatesy et al. [55]) were used to calculate hip height via trigonometry (Fig. 3), which was then used to calculate speed at Froude numbers of 1 and 16, as above.

**Table 4:** Effects of epiphysis size on hindlimb length and estimated speed at Fr = 1 (slow running) in representative non-theropod dinosaurs with a columnar limb posture.

Taxon	Hindlimb length (m)				Speed at Slow Running (Froude = 1), ms^−1^				
	No CCF	*Alligator* CCF (10.8%)	*Struthio* CCF (6.8%)	*Coturnix* CCF (1.8%)	V (no CCF)	V (*Alligator* CCF)	V (*Struthio* CCF)	V (*Coturnix* CCF)	Mean V
*Protoceratops* *	0.65	0.72	0.69	0.66	2.52	2.65	2.60	2.54	2.58
*Triceratops* *	1.81	2.01	1.94	1.85	4.22	4.44	4.36	4.25	4.32
*Edmontosaurus* *	2.62	2.91	2.80	2.67	5.07	5.34	5.24	5.12	5.19
*Thecodontosaurus* *	0.18	0.20	0.19	0.18	1.32	1.39	1.36	1.33	1.35
*Brachiosaurus* *	3.43	3.80	3.66	3.49	5.79	6.10	5.99	5.85	5.93
*Camarasaurus* *	2.34	2.59	2.50	2.38	4.79	5.04	4.95	4.83	4.90
*Diplodocus* *	2.75	3.05	2.94	2.80	5.19	5.46	5.36	5.24	5.12

CCF, correction factor,

*, feet not included in limb length estimate.

**CCF**  =  mean change in lengths of femur plus tibia (from Table 1). *Alligator* CCF = 10.8%; Adult *Struthio* CCF = 6.8%; *Coturnix* CCF = 1.8%). Slow-running velocity (V) calculated by equation: v =  sqrt(1*9.8 ms^−2^*limb length[m]). Limb length data were taken from Marsh [29], Brown and Schlaikjer [121], Lull and Wright [122], Mazzetta et al. [88], and Royo-Torres et al. [89].

## Results

### Quantitative results

Table 1 presents means and standard deviations for percent change in limb element dimensions, as well as the results of the intraspecific paired sample t-tests. File S1 presents the raw measurement data used in the analysis.

#### Alligator (*Alligator mississippiensis*)

Mean percent changes in lengths of elements as a result of skeletonization ranged from a low of 4.52% in the tibia to a high of 8.58% in the ulna (see Table 1). Mean percent changes in condylar dimensions were much larger than length changes, resulting in a range of about 9% (femur, distal CC) to about 27% (humerus, proximal ML). All changes in alligator limb elements after skeletonization were significant (*p*<0.01).

#### Ostrich (*Struthio camelus*)

Mean percent changes in lengths of elements as of result of skeletonization ranged from a low of 2.07% in the tibia to a high of 4.69% in the femur (see Table 1). In the one whole-individual adult ostrich, the ulnae changed 4.20% and radii 3.00%. Mean condylar breaths changed between 1.95% (tibia, proximal CC) and 13.39% (fibula, proximal CC). Almost all changes in adult ostrich limbs were statistically significant (*p*<0.01). The subadult ostrich limb elements changed more than the adults: humerus, 3.72%; femur, 6.40%; tibia, 4.38%. Changes in the subadult ostrich were all significant (*p*<0.01).

#### Quail (*Coturnix japonica*)

Quail limb element dimensions changed less than those of ostrich and alligator after skeletonization. Greatest length changed between an average of 0.56% (humerus) and 2.36% (ulna) which were non-significant changes. Condylar dimensions generally changed more than length with the smallest mean change being 0.68% (ulna, proximal CC) and largest being 10.77% (humerus, distal CC). Only a few distal condylar measures in quail limbs changed significantly after skeletonization.

#### Chicken (*Gallus gallus*)

The two chickens studied differed greatly from each other in the amount of change after skeletonization, but had results comparable to the alligators. Mean percent change in greatest length ranged from 3.89% (ulna) to 9.54% (femur), and condylar dimensions ranged from 3.63% (radius, proximal ML) to 31.47% (femur, distal ML). Although the percent change was substantial, paired sample t-tests were not applicable due to the small sample size.

#### Wood duck (*Aix sponsa*)

The single wood duck specimen showed less change as a result of skeletonization than did the other bird specimens in that the greatest lengths of all limb elements changed by less than one percent. Condylar dimensions changed between 0.92% (radius, proximal ML and distal ML) and 13.41% (humerus, proximal ML).

### Comparisons between groups

Statistical comparisons between different groups of specimens are presented in Table 2. Two sample t-tests of the arcsin-transformed percent change among limb elements in subadult and adult ostriches had varied outcomes. For example, differences between the two ostrich age-classes in femoral greatest length were significant (*p*<0.01) whereas humeral and tibial greatest lengths were not significantly different. Several condylar dimensions were significantly different (*p*<0.01), such as the proximal craniocaudal dimension in all elements and the humeral distal mediolateral dimension; but most were not. Comparisons between alligators and ostriches in percent change after skeletonization resulted in many dimensions being significantly different (*p*<0.01). However, the changes in the proximal craniocaudal dimension of the humerus and the greatest lengths of the femur and tibia did not differ significantly between the two clades.

### Effects of body size on epiphysis size in *Alligator*

One might predict that the amount of epiphyseal cartilage will decrease with increasing size and age; that is, as an animal ages, it would tend to ossify existing cartilage. Thus, a general prediction would be negative allometry among the cartilaginous contributions to a limb element. The results of the regression analysis found 15 of the 32 comparisons to be significant (Table 3). Of these, eight reflected the predicted negatively allometric trend (e.g., femoral, ulnar, and radial length), and seven reflected isometry (e.g., fibular length).

### Qualitative anatomical changes

Changes in anatomical shape attributes after skeletonization were most evident in alligators, in which many cartilaginous articular surfaces that had functionally important roles in life virtually disappeared after the removal of the epiphyseal cap. Such large changes also characterized the subadult ostrich, and to a less extent the adult ostriches. On the other hand, the other avian taxa studied changed very little in articular morphology after the removal of the epiphyseal cartilage.

Alligator limb bones have a large amount of epiphyseal cartilage on their proximal and distal ends. For example, femora (which are often used in comparative investigations) change not only ∼7% in length, but also 15% in the condylar width. With cartilage intact, the femoral head is much more pronounced and rounded (with a medially oriented ball; compare Fig. 4A, B) than that which is preserved in the skeleton. The distal femoral condyles are also enlarged, more acutely defined, and rectangular in shape (Fig. 4C, D), whereas the bony surface is simpler, smooth, and more ill-defined.

In addition to these general changes, several exclusively cartilaginous joint structures were lost. In the elbow, the distal condyles of an alligator humerus are composed of large cartilaginous condyles that differ substantially from the underlying bone's shape (Fig. 5A, B). Likewise, the proximal ulna has a large cartilaginous protuberance, the intercotylar process, which articulates with the olecranon fossa of the distal humerus. In the living animal, these structures form a highly congruent elbow joint that appears to restrict transverse movement and extension. However, upon skeletonization, these structures are barely noticeable and the functional joint is non-existent (Fig. 5C, D). Our findings regarding the elbow joint of crocodylians broadly agree with those of Fujiwara et al. [11]. In the knee, the cartilaginous femoral condyles are more pronounced and defined compared to their underlying bony surface. On the other hand, the bony portion of the tibial plateau (not figured) best approximated the shape of the overlying cartilaginous epiphysis.

Although we have metric data on only *Alligator mississippiensis*, we are certain that these findings pertain to crocodylians generally. A survey of extant crocodylian osteology in museum collections and the literature reveals the generality of the same bony morphology observed in alligators [70]–[72]. Moreover, dissection of *Crocodylus johnstoni* (OUVC 10425) confirms that, as in alligators, the ends of the fleshy limb elements exhibit large epiphyses and relatively complicated condylar morphology, which likewise are lost during skeletonization. Again, Fujiwara et al. [11] reported similar findings for four other extant crocodylian species.

**Figure 6:**
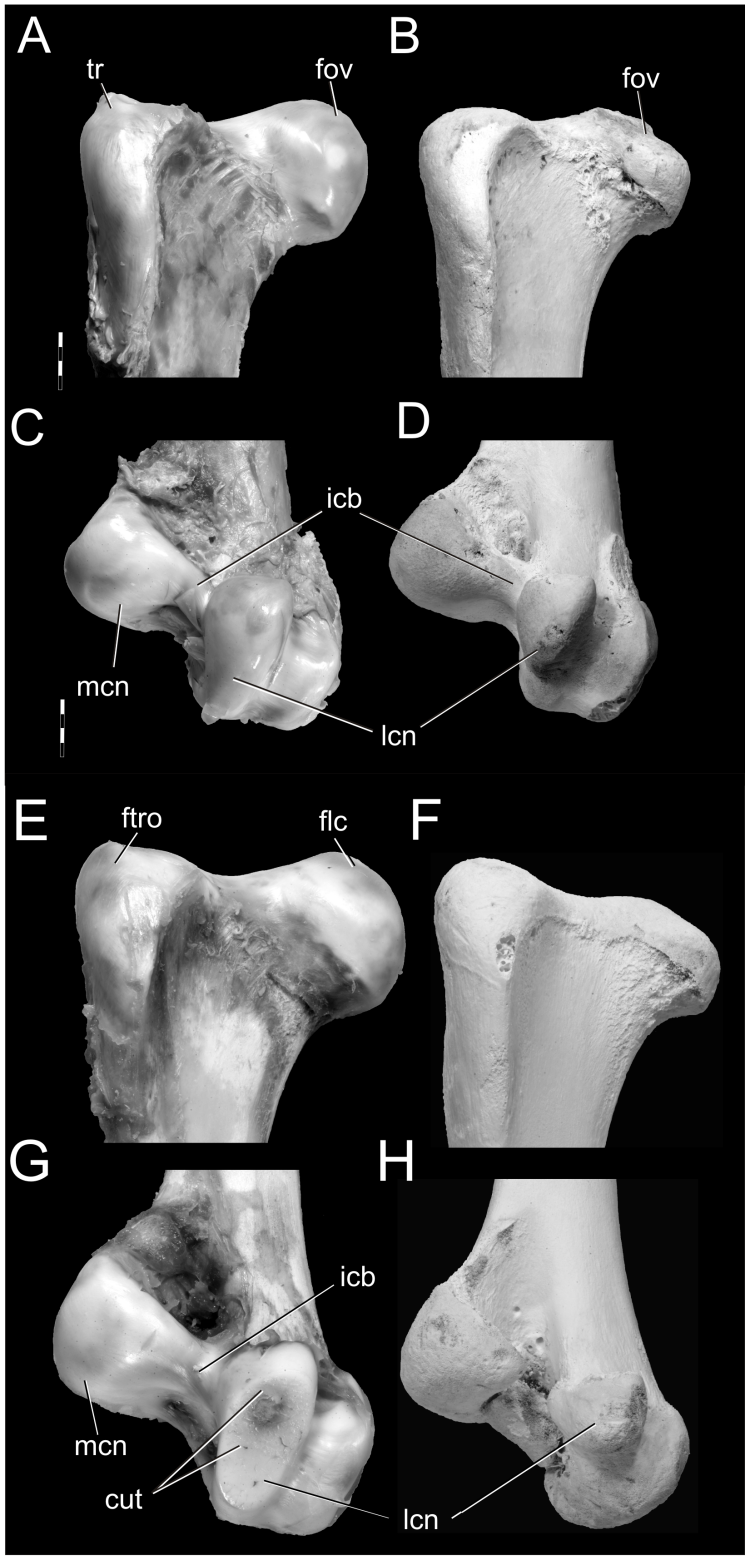
Changes in epiphyses of *Struthio camelus*. Adult (**A**–**D**; OUVC 9438) and subadult (**E**–**H**; OUVC 9439) right femora before (**left**) and after (**right**) skeletonization. **A,** **B**: adult proximal end, cranial view. **C**, **D**: adult distal end, caudal view. **E**, **F**: subadult proximal end, cranial view. **G**, **H**: subadult distal end, caudal view. Abbreviations: **cut**, cut portion of cartilaginous epiphysis; **fov**, fovea; **icb**, intercondylar bridge; **lfc**, lateral femoral condyle; **mfc**, medial femoral condyle; **tr**, femoral trochanter. Scale bar increments equal 0.5 cm.

The cartilaginous condylar morphologies of subadult and adult ostriches were very similar, which made it difficult to judge the age class of the functional (or fleshy) limb elements. Following skeletonization, however, the bony morphologies were clearly different. In adult ostrich femora, the articular cartilage formed a reasonably thick cap on the craniodorsal surface of the head and forms the fovea ligamentum capitis [73] (Fig. 6A, B). The distal lateral condyle is composed of a large amount of cartilage that expands the functional surface cranially and medially (Fig. 6C, D), whereas the distal medial condyle is covered only by a thin layer of cartilage that only expands the shape slightly. Bony condylar texture was smooth on both ends of adult ostrich limbs. With skeletonization of subadult ostrich femora, remarkable shape changes occurred after the large cartilaginous cap was removed. Epiphyseal cartilage formed a thick cap extending from the medial rim of the femoral head, over the trochanteric fossa, and over the antitrochanteric articular surface (Fig. 6E, F). Distally, there was an even greater change in shape, because most of the condylar architecture was composed of cartilage (Fig 6G, H). The intercondylar bridge (Fig. 6) completely disappears between the largely cartilaginous medial and lateral condyles. The bone surface was heavily scarred by vascular grooves and pits, and the lamina of calcified cartilage was not uniformly distributed across the surface. Similar changes in condylar architecture were observed in the tibiae, fibulae, and humeri of subadults. Quail and duck showed no obvious changes in condylar morphology after skeletonization.

## Discussion

Crocodylians, palaeognaths, and neognaths are considerably different with regard to ossification of the ends of their limb bones. Crocodylians have a substantial amount of cartilage, such that skeletonization—either in the laboratory or in nature—strips away much of the functionally relevant anatomy, both quantitatively and qualitatively. Significant shape changes occurred in the condylar morphology with the loss of the epiphyseal cartilaginous cap, often removing key articular structures responsible for joint articulation, mobility, and congruence. Therefore, skeletonized crocodylian bones are poor representatives of what the living animal actually used. Ostrich elements also retain a large amount of cartilage, although not as much as in crocodylians. In general, skeletonized adult neognath bird bones are more faithful representations of the living element than those of ostrich and crocodylians and lose only negligible amount of tissue to skeletonization. However, although not nearly as dramatic as in crocodylians, some elements do exhibit statistically significant changes in dimensions, suggesting that the problem cannot be discounted even in birds.

In alligators and less so in birds, epiphyseal cartilage is responsible for joint congruence, angulation, and hence posture. However, unlike mammals, bony condylar morphology does not necessarily accurately represent the morphology of the functional articular end [9], [74]. Joint angulation and congruence may be affected by expansions of cartilage from the bony condyles, as in alligator distal femoral condyles (Fig. 3), and further by purely cartilaginous articular structures, as in the alligator proximal ulna (Fig. 4). Although not featured as part of the primary analysis, profound changes in size and morphology were also found in the pectoral girdles (e.g., glenoid width and depth) and pelvic girdles (e.g., acetabular width and depth) of both alligators and ostrich, indicating that these joints are also built by large amounts of articular cartilage. These findings demonstrate that common reconstructions of archosaur limb posture [47], [48], [53], [55], [75]–[82] may be affected by this missing and likely important functional information. Despite this missing morphology, Hutchinson and Gatesy [53] and Gatesy et al. [55] have shown that, among all possible joint angles in the limb, there is only a limited range of biomechanically feasible postures. Regardless of posture, the presence of extensive cartilaginous epiphyses in large, non-avian dinosaurs likely impacted the mechanical loading environment of the appendicular skeleton. For example, if a 1.9 m-long femur of the sauropod dinosaur *Apatosaurus* possessed cartilaginous epiphyses comparable to those found in alligators (i.e., 6.5% of total femoral length, with equal cartilage thicknesses on the proximal and distal ends; Table 4), there would be at least 5 cm of epiphyseal cartilage covering each end of the femur. If cartilaginous condyles sufficient in size to maintain joint congruence are envisioned, this thickness would increase even more. Therefore, if cartilaginous epiphyses do absorb loads imparted onto limbs during locomotion [83]–[85], then these soft-tissue structures, along with other recognized morphological changes in limb structure such as element eccentricity [86] and limb-stance gauge [81] found among sauropods and other non-avian dinosaurs, may help alleviate the huge loads likely experienced by these massive animals.

### Element length and Cartilage Correction Factors

Large epiphyseal cartilaginous caps create longer functional elements which increase limb excursion estimates and thus speed estimates in dinosaurs [45], [55], [68], [87]. To illustrate this relationship, lengths of hindlimb elements (femur, tibia, metatarsus) from representative ornithischians and sauropods, and lengths of all hindlimb elements (i.e., femur, tibia, ankle, foot) of theropod dinosaurs were obtained from the literature [55], [69], [88], [89]. The lengths of the femora and tibiae were modified using three different cartilage correction factors (CCF) (alligator, ostrich, quail) to account for the missing epiphyseal cartilage (Table 4) whereas metatarsal and foot measurements were left as is. For example, the partial hindlimb length (i.e., femur, tibia, metatarsal) of *Tyrannosaurus* (MOR 555) using only bony elements (i.e., with no correction factor) and a columnar stance is approximately 3.089 m [69], whereas after the addition of cartilaginous epiphyses, hindlimb length extends to between 3.42 m (*Alligator* CCF) and 3.14 m (*Coturnix* CCF). Partial hindlimb length (i.e., femur, tibia, metatarsal) of the sauropod *Brachiosaurus* ranges from 3.80 m (*Alligator* CCF) to 3.43 m (no CCF). These modifications in overall limb length are modest in some respects (e.g., less than half a meter), however not trivial, because they add as much as 0.4 m (1.3ft) to the length of a femur or tibia.

### Speed, posture, and Cartilage Correction Factors

Slow running speed estimates for a columnar-postured *Tyrannosaurus* range from 6.00 ms^−1^ (no CCF) to 6.32 ms^−1^ (*Alligator* CCF) and for *Brachiosaurus* (excluding pedal length for ease of comparison), 5.79 ms^−1^ (no CCF) to 6.10 ms^−1^ (*Alligator* CCF) (Table 5). As expected, these speeds in sauropods are similar to those estimated by Alexander [90], but faster than those estimated from trackway evidence [91]. The slow running speeds of *Triceratops* (4.22–4.44 ms^−1^) and *Edmontosaurus* (5.07–5.34 ms^−1^) vary by similar amounts (Table 4) based on CCF application. The inclusion of postural changes (i.e., crouched, “ostrich-like”) with CCF expectedly decreased the estimated speed of *Tyrannosaurus* and other theropods compared to columnar postures (Table 5). Thus, although the effect of inclusion of CCFs into speed estimates proves to be relatively modest, perhaps adding ∼0.6 ms^−1^ (2.1kph, 1.2mph) to an estimate, these data do further refine and narrow the range of error that may impact inferences about the locomotor behavior of extinct taxa. Regardless, the impact of this corrective factor is somewhat miniscule when set within the scope of variability in locomotor estimates of fossil taxa as a whole given the numerous sources of error, such as substrate, posture, body mass, and center of mass, to name a few [53], [55], [68], [69], [92]. Hutchinson et al. [57] and Gatesy et al. [55] both showed that an increase in limb length would require even more extensor muscle mass. Therefore, particularly in crouched taxa like theropod dinosaurs, an increase in limb length may actually lead to a decrease in possible speed.

**Table 5:** Effects of articular cartilage and posture on forward velocity of slow running and fast running theropod dinosaurs.

Taxon	No CCF						Gator CCF						Ostrich CCF					
	*T. rex*		*Struthio*		Col		*T. rex*		*Struthio*		Col		*T. rex*		*Struthio*		Col	
	FR 1	FR 16	FR 1	FR 16	FR 1	FR 16	FR 1	FR 16	FR 1	FR 16	FR 1	FR 16	FR 1	FR 16	FR 1	FR 16	FR 1	FR 16
*Herrerasaurus*	2.77	11.09	2.95	11.28	3.09	11.21	2.82	11.78	3.00	12.00	3.15	11.92	2.80	12.36	2.98	12.59	3.13	12.51
*Coelophysis*	2.24	8.97	2.36	9.10	2.47	9.05	2.28	9.46	2.40	9.61	2.51	9.55	2.26	9.89	2.39	10.05	2.50	9.99
*Dilophosaurus*	3.67	14.68	3.88	14.90	4.06	14.81	3.72	15.50	3.94	15.75	4.13	15.65	3.70	16.23	3.91	16.50	4.10	16.40
*Allosaurus*	4.02	16.09	4.27	16.36	4.48	16.26	4.09	17.08	4.35	17.39	4.56	17.28	4.06	17.93	4.32	18.26	4.53	18.13
*Compsognathus*	1.35	5.39	1.42	5.47	1.49	5.44	1.37	5.69	1.44	5.78	1.51	5.74	1.36	5.95	1.44	6.05	1.50	6.01
*Velociraptor*	2.12	8.48	2.25	8.62	2.35	8.56	2.15	8.98	2.28	9.14	2.39	9.08	2.14	9.41	2.27	9.58	2.38	9.51
*Archaeopteryx*	1.24	4.97	1.31	5.04	1.36	5.01	1.26	5.22	1.33	5.30	1.39	5.27	1.25	5.46	1.32	5.54	1.38	5.51
Small tyrannosaur	3.29	13.17	3.47	13.37	3.63	13.29	3.34	13.89	3.53	14.11	3.69	14.02	3.32	14.53	3.51	14.76	3.67	14.67
*Tyrannosaurus*	5.41	21.63	5.72	21.97	6.00	21.84	5.49	22.90	5.82	23.28	6.10	23.14	5.46	24.00	5.78	24.41	6.06	24.26

***T. rex*** posture (124°, knee; 147°, ankle), ***Struthio*** posture (109°, knee; 142°, ankle), and columnar (**Col**) posture. All values are ms^−1^. Limb length data were taken from Sereno and Arcucci [79] and Hutchinson [69].

Besides their utility in speed estimates, bony limb element lengths are often used to determine intra- and interspecific scaling patterns in archosaurs [88], [93]–[101]. However, the presence of a significantly large epiphyseal cartilaginous cap would have a wide-ranging affect on these comparisons because (1) bony and functional limb element lengths differ, (2) proximal and distal ends differ in the amount of cartilage, (3) different limb elements have relatively different amounts of epiphyseal cartilage, (4) ontogenetic differences in articular cartilage may exist in some but not all taxa, and (5) different clades of amniotes vary in the amounts of epiphyseal cartilage hence confounding broad intraspecific comparisons. Any of these changes may impact the slope, intercept, and correlation coefficient of a regression analysis and thus impact estimations of body mass.

Finally, our results, as well as those of Fujiwara et al., [11] and Bonnan et al., [59] indicate missing epiphyseal and articular cartilages significantly alter the articular morphology of limb bones in non-avian archosaurs. Whereas some postural insight may be gleaned from articulated specimens [102] or even careful manual manipulations [52], the architecture of cartilaginous structures such as intercondylar processes and olecranon processes, as well as the menisci and ligaments that undoubtedly attached to these cartilaginous surfaces, are lost, along with their osteological (or cartilaginous) correlates. Moreover, the taphonomic processes involved during rapid burials of even the best-preserved specimens may impact soft-tissue anatomy and posture, via twisting, separation, or compaction in unclear ways [103]. We do not suggest that these reconstructions and tests are impossible, but we suggest that explicit care and hypothesis testing be incorporated where limb posture is crucial to the forwarding of functional explanations and inferences of behavior.

### Osteological correlates and extinct archosaur femoral articular morphology

**Figure 7:**
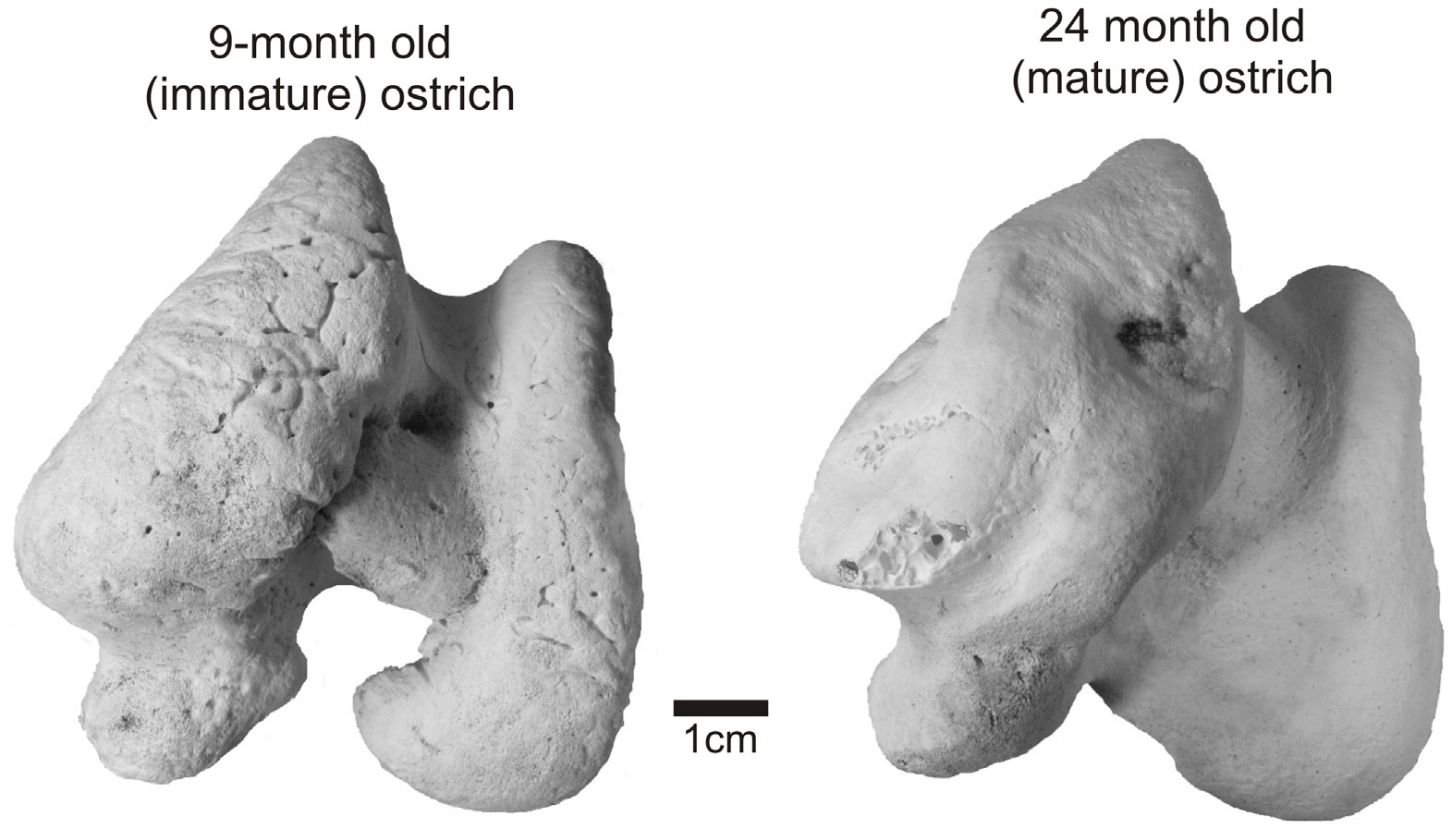
Condylar surface texture in the distal femora of *Struthio camelus*. **A**: adult (OUVC 9439); **B**: subadult (OUVC 9438) ostrich. Scale bar equals 1 cm.

Following skeletonization, the lamina of calcified cartilage is the closest representative of the functional articular surface and has often been identified as the scar left by the epiphyseal cartilage [26], [104]. In healthy adult mammals, turtles, lizards, crocodylians, and birds, this surface is almost always smooth and simple [9]. In alligators, the lamina of calcified cartilage is continuous across the terminal bony surface and expands around the periphery of the articular structures forming an equatorial scar around the widest part of the metaphysis (Fig. 1A). Like those of mammals, adult bird limb bones are characteristically different in that the lamina is restricted to the terminal articular surface and is not always continuous across different bony condylar structures. This extremely thin layer of calcified cartilage, however, is easily damaged in fossils during preservation and preparation and may not be an accurate reflection of the original morphology in the fossil bone. Textural differences are noticeable between the condyles of subadult and adult ostrich limbs (Fig. 7). Despite their equivalent sizes, younger individuals have pitted, porous, and generally unfinished textures that show bony signatures of vascular canals.

**Figure 8:**
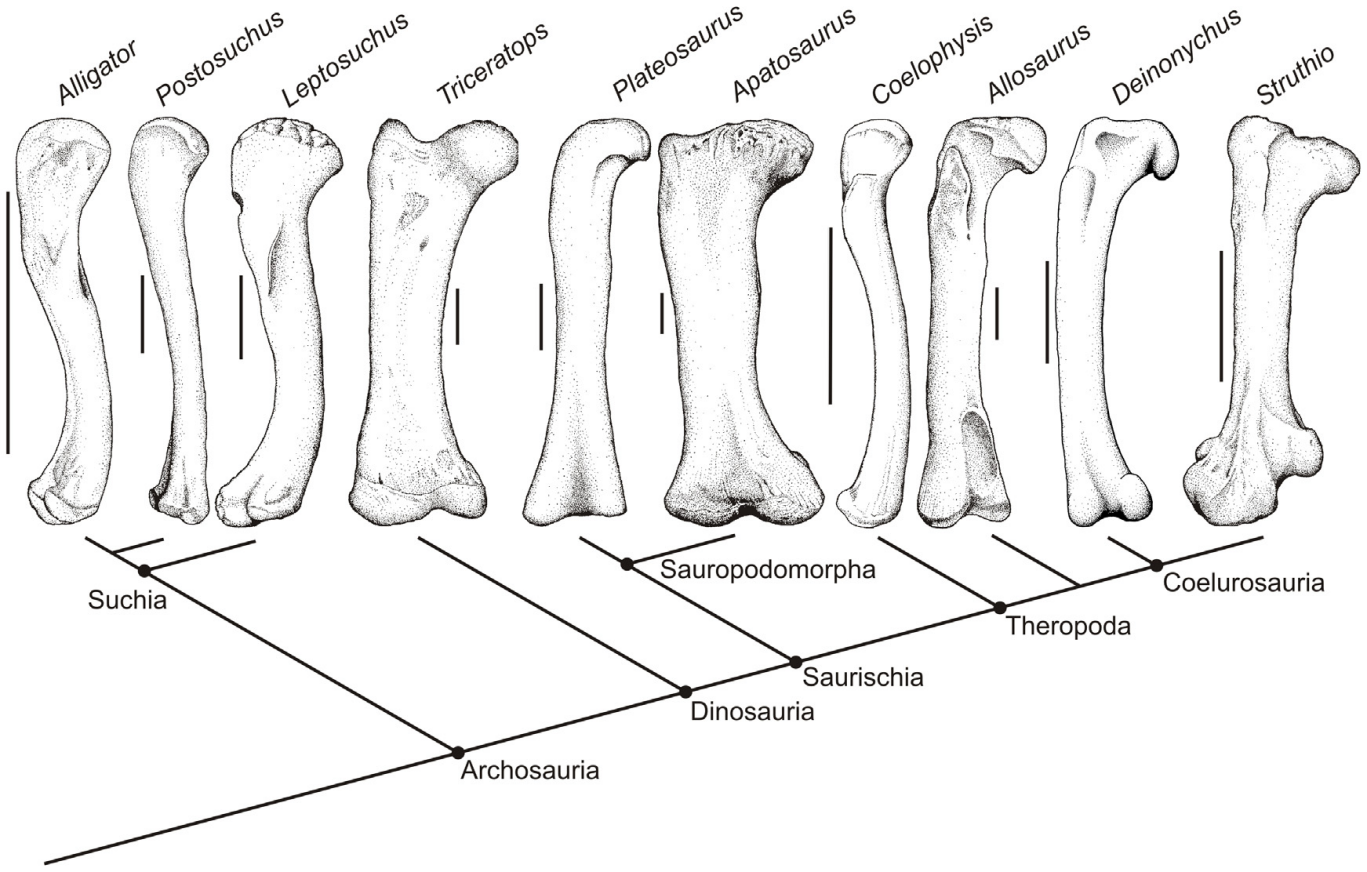
Skeletonized femora of living and extinct archosaur taxa illustrating lack of bony condylar structures, suggesting the presence of significant amounts of epiphyseal cartilage. *Postosuchus* redrawn from Chatterjee [105], *Leptosuchus* modified from Long and Murray [107]; *Triceratops* modified from Dodson [80]; *Plateosaurus* modified from Galton and Upchurch [108]; *Apatosaurus* modified from Ostrom and McIntosh [110]; *Coelophysis* modified from Colbert [109]; *Allosaurus* modified from Madsen [113]; *Deinonychus* modified from Ostrom [114]. Scale bar equals 10 cm.

With the above knowledge in mind, a more accurate investigation into the loss of epiphyseal cartilage may be made by comparing the ends of archosaurian femora. The terminal morphologies of alligator femora and those of other early archosaurs are very similar (Fig. 8). The terrestrial rauisuchid *Postosuchus* [105] and semi-aquatic phytosaur *Leptosuchus* [106], [107] both have simple, convex proximal and distal ends with no articular structures (Fig. 8). A similar terminal morphology is present in the prosauropod *Plateosaurus* [108] and in the early theropod *Coelophysis* [109]. In these taxa, the laminae of calcified cartilage expand to the peripheral (equatorial) margins of the epiphyses and encapsulate the terminal bony condyles. These basal taxa lack the well-defined articular structures similar to the cartilaginous ones present in extant crocodylians or the bony structures in adult ostrich. Thus, it appears that these taxa likely had largely cartilaginous articular structures.

Ornithischian dinosaurs, albeit diverse, show fairly uniform terminal morphology in their femora. For example, *Triceratops* [80] (Fig. 8) does appear to have a well demarcated femoral head. However, its distal condyles are only slightly convex and seem to offer little support for a congruent articulation with the tibial plateau. Such morphology is fairly typical among ceratopsian dinosaurs, and it seems likely that these animals possessed significant amounts of epiphyseal cartilage. Sauropods, such as *Camarasaurus* [110] (Fig. 8), have no discernible articular structures, and, in agreement with the early hypotheses of Marsh [111] and Cope [112], must have had extensive epiphyseal cartilaginous caps. The roughened articular texture of most sauropod limbs is most similar to the bony ends of subadult ostrich femora (Fig. 7) in which there is an undulating chondroosseous junction [19].

Bird-like articular structures become more evident during theropod evolution, including postural changes and the development of a well-defined femoral neck [53], [69], [82]. In *Allosaurus* [113], *Tyrannosaurus*, *Struthiomimus*, and *Deinonychus* [114], the femoral head and distal condyles are smooth and more distinct compared to those found in sauropods and ornithischians. Unlike more basal theropod taxa such as *Coelophysis* in which the calcified cartilage is present around the peripheral margins of the condyles, the lamina of calcified cartilage persists only on the most terminal portions of the bony condyles in most coelurosaurs (Fig. 8). Therefore, it is reasonable to hypothesize that derived theropod dinosaurs had less epiphyseal cartilage than other dinosaurs and the amount of cartilage decreased on the theropod lineage leading to birds. Regardless, a large amount may still have been present, and given the significant contribution from cartilage to the breadth of epiphyseal surfaces, hypothesizing congruence, posture, and range of motion in the joints of fossil archosaurs remains quite challenging.

### Significance of large cartilaginous epiphyses

Longitudinal growth of long bones is due to the proliferation, hypertrophy, and subsequent apoptosis of chondrocytes, which produce extracellular matrix [15] [115]. In mammals and birds, growth ceases after these cells are exhausted and bony tissues replace the cartilage, in turn leaving only a thin layer of articular cartilage. This does not appear to be the case in alligators and turtles, and the presence of large cartilaginous epiphyses in non-avian dinosaurs suggests that these animals not only might have maintained large reservoirs of chondrocytes and extracellular matrix-producing cells in their epiphyses, but that these epiphyses may have remained cartilaginous because bone growth simply did not overtake cartilage development. Although these hypotheses require testing in relevant extant taxa (e.g., young crocodylians), they do support current hypotheses that sauropods and other dinosaurs may have achieved fast growth rates [116]–[119].

In addition, mature hyaline and articular cartilages are generally avascular in mammals and birds and rely on diffusion to supply necessary nutrients [15], [120]. During growth, vascular canals perforate the chondroosseous junction to supply the cartilage and leave the characteristic rugose, perforated texture found in immature mammals and birds, as well as mature non-avian dinosaurs. However, if some sauropod cartilaginous epiphyses were actually 5–7 cm thick (alligator CCF), and 20–50 cm wide in either direction across the condylar surfaces with similar correction factors applied, this results in a conservative estimate of approximately 5000–7000 cubic centimeters of cartilage! Therefore, passive or locomotion-driven diffusion was not likely an adequate means of nutrition, and vascular canals were likely necessary minimally to maintain healthy epiphyseal cartilage. Finally, the discovery of fossilized cartilaginous tissue relatively proximal to the end of the humerus of *Cetiosaurus* [34] further suggests that the cartilage caps of sauropods may have been larger than those predicted by an *Alligator* CCF and that the caps extended fairly far onto the metaphysis of some long bones. Therefore, rugose condylar textures and their inferred soft tissues seem necessary regardless of growth rate in at least sauropods, if not other large-bodied taxa. Because crocodylians, turtles, some non-avian dinosaurs, many small-bodied non-avian theropods, and most birds simply have smaller absolute volumes of epiphyseal cartilage than those found in the largest dinosaurs, diffusion may still be feasible and, like mammals and lizards, these taxa eliminate most vascular canals early in ontogeny and maintain smooth bony condylar surfaces. Therefore, the presence of large cartilaginous epiphyses in mature individuals (e.g., like those ubiquitous in sauropods and ornithischians) suggests that: (1) these cartilaginous structures may be paedomorphic—individuals retain juvenile epiphyseal structures into maturity; (2) the rugose condylar morphologies and their inferred vascular channels are functional necessities to maintain these absolutely huge cartilaginous structures; and (3) avian-style epiphyseal morphologies likely evolved during coelurosaurian theropod evolution.

## Supporting Information

File S1 Holliday et al. 2010 PLOS ONE Archosaur Epiphyses.
(1.29 MB DOC)

## References

[1] McCutchen C, Hall BK (1983). Lubrication of and by articular cartilage.. Cartilage: structure, function, and biochemistry.

[2] Baumel JJ, Raikow R, Baumel JJ (1993). Arthrologia.. Handbook of Avian Anatomy: Nomina Anatomica Avium 2nd ed.

[3] Carter D, Mikic B, Padian K (1998). Epigenetic mechanical factors in the evolution of long bone epiphyses.. Zool J Linn Soc.

[4] Enlow D, Owen R, Goodfellow J, Bullough P (1980). Dynamics of skeletal growth and remodeling.. Scientific Foundations of Orthopaedics and Traumatology.

[5] Moodie R (1908). Reptilian epiphyses Amer J Anat.

[6] Lubosch W (1924). Die Bildung des Markknochens beim Hühnchen und bei Säugetieren und das Wesen der endchondralen Ossifikation in historischer Betrachtung.. Morphologie Jarbusch.

[7] Haines R (1939). The structure of the epiphysis in *Sphenodon* and the primitive form of secondary centre.. J Anat.

[8] Haines RW (1941). Epiphyseal structure in lizards and marsupials.. J Anat.

[9] Haines RW (1969). Epiphyses and Sesamoids.. Biology of the Reptilia.

[10] Enlow D, Gans C (1969). The Bone of Reptiles.. The Biology of the Reptilia Volume 1 Morphology A.

[11] Fujiwara S, Taru H, Suzuki D (2010). Shape of articular surface of crocodilian (Archosauria) elbow joints and its relevance to sauropsids.. J Morphol.

[12] Haines RW (1942). The evolution of epiphyses and of endochondral bone.. Biol Rev.

[13] Moss M, Moss-Salentijn L, H BK (1983). Cartilage: structure, function and biochemistry..

[14] Kronenberg H (2003). Developmental regulation of the growth plate.. Nature.

[15] Hall BK (2005). Bones and cartilage: developmental and evolutionary skeletal biology..

[16] Reid R (1996). Bone histology of the Cleveland-Lloyd Dinosaurs and of dinosaurs in general, Part I: introduction to bone tissues.. BYU Geol Stud.

[17] Reid R, Ferguson M (1984). The histology of dinosaurian bone and its possible bearing on dinosaurian physiology.. Zool Soc Lond Symp 52 The structure, development and evolution of reptiles.

[18] de Ricqlès A, Hall BK (1991). Comparative microstructure of bone.. Bone: bone matrix and bone specific products.

[19] Barreto CR, Albrecht M, Bjorling DE, Horner JR, Wilsman NJ (1993). Evidence of the growth plate and the growth of long bones in juvenile dinosaurs.. Science.

[20] Haines RW, Gans C (1969). Epiphyses and sesamoids.. Biology of the Reptilia Volume 1 Morphology A.

[21] Geist N, Jones T (1996). Juvenile skeletal structure and the reproductive habits of dinosaurs.. Science.

[22] Simon W (1970). Scale effects in animal joints: 1. Articular cartilage thickness and compressive stress.. Arth Rheumat.

[23] Eckstein F AC, Sittek H, Becker C, Milz S, Schulte E (1997). Non-invasive determination of cartilage thickness throughout joint surfaces using magnetic resonance imaging.. J Biomech.

[24] Egger GF, Witter K, Weissengruber G, Forstenpointer G (2008). Articular cartilage in the knee joint of the African elephant, *Loxodonta africana*, Blumehbach 1797.. J Morphol.

[25] Owen R (1875). Monographs of the British Fossil Reptilia of the Mesozoic Formations. Part II. (Genera *Bothriospondylus*, *Cetiosaurus*, *Omosaurus*).. Palaeontographical Society Monographs.

[26] Cope E (1878). On the saurians recently discovered in the Dakota Beds of Colorado.. Amer Nat.

[27] Cope E (1884). Marsh on *Diplodocus*.. Amer Nat.

[28] Marsh O (1883). Principle characters of American Jurassic dinosaurs. Part VI: Restoration of Brontosaurus.. Amer J Sci.

[29] Marsh O (1896). The dinosaurs of North America.. USGS 16th Ann Rep.

[30] Osborn H (1898). Additional characters of the great herbivorous dinosaur *Camarasaurus*.. Bull Amer Mus Nat Hist.

[31] Hatcher J (1901). *Diplodocus* (Marsh): its osteology, taxonomy, and probable habits, with a restoration of the skeleton.. Mem Carn Mus.

[32] Hay O (1908). On the habits and the pose of the sauropodous dinosaurs, especially of *Diplodocus*.. Amer Nat.

[33] Bakker R (1971). Ecology of the brontosaurs.. Nature.

[34] Schwarz D, Wings O, Meyer C (2007). Super sizing the giants: first cartilage preservation at a sauropod limb joint.. J Geol Soc London.

[35] Coombs WJ (1975). Sauropod habits and habitats.. Palaeogeography, Palaeoclimatology, Palaeoecology.

[36] Norman D (1980). On the ornithischian dinosaur *Iguanodon bernissartensis* from the Lower Cretaceous of Bernissart (Belgium).. Institut Royal des Sciences Naturelles de Belgique Memoire.

[37] Nicholls E, Russell A (1985). Structure and function of the pectoral girdle and forelimb of *Struthiomimus altus* (Theropoda: Ornithomimidae).. Palaeont.

[38] Carpenter K (1999). Eggs, Nests, and Baby Dinosaurs: a look at dinosaur reproduction..

[39] Wilson J, Sereno P (1998). Early evolution and higher-level phylogeny of sauropod dinosaurs.. J Vert Paleont Society of Vertebrate Paleontology Memoir 4.

[40] Wilson J, Currey Rogers KA Wilson J (2005). Overview of sauropod phylogeny and evolution.. The Sauropods: evolution and paleobiology.

[41] Bakker R (1986). The Dinosaur Heresies: William Morrow, New York City.

[42] Parrish J (1986a). Locomotor adaptations in the hindlimb and pelvis of the Thecodontia.. Hunteria.

[43] Parrish J, Padian K (1986b). Structure and function of the tarsus in the phytosaurs (Reptilia: Archosauria).. The Beginning of the Age of Dinosaurs.

[44] Hallett M, Czerkas S, Olson E (1987). The scientific approach to the art of bringing dinosaurs to life.. Dinosaurs past and present, Volume 1.

[45] Paul G (2002). Dinosaurs of the air..

[46] Paul G (1998). Predatory dinosaurs of the world: a complete illustrated guide..

[47] Paul G, Christiansen P (2000). Forelimb posture in neoceratopsian dinosaurs: implications for gait and locomotion.. Paleobiol.

[48] Johnson R, Ostrom J, Thomason J (1995). The forelimb of *Torosaurus* and an analysis of the posture and gait of ceratopsian dinosaurs.. Functional Morphology in Vertebrate Morphology.

[49] Gishlick A, Gauthier J, Gall L (2001). The function of the manus and forelimb of *Deinonychus antirrhopus* and its importance for the origin of avian flight.. New perspectives on the origin and early evolution of birds.

[50] Bonnan M (2004). Morphometric analysis of humerus and femur shape in Morrison sauropods: implications for functional morphology and paleobiology.. Paleobiology.

[51] Senter P, Robin J (2005). Range of motion in the forelimb of the theropod dinosaur *Acrocanthosaurus atokensis* and implications for predatory behaviour.. J Zool.

[52] Senter P (2005). Function in the stunted forelimbs of *Monoykus olecranus* (Theropoda), a dinosaurian anteater.. Paleobiol.

[53] Hutchinson J, Gatesy S (2006). Dinosaur locomotion: beyond the bones.. Nature.

[54] Bonnan MF, Senter P, Barrett PM, Batten D (2007). Were the basal sauropodomorph dinosaurs *Plateosaurus* and *Massospondylus* habitual quadrupeds?. Evolution and palaeobiology of early sauropodomorph dinosaurs: Special Papers in Palaeontology.

[55] Gatesy S, Bäker M, Hutchinson JR (2009). Constraint-based exclusion of limb poses for reconstructing theropod dinosaur locomotion.. J Vert Paleont.

[56] Thulborn R (1982). Speeds and gaits of dinosaurs.. Palaeogeog Palaeoclim Palaeoeco.

[57] Hutchinson JR, Anderson F, Blemker S, Delp S (2005). Analysis of hindlimb muscle moment arms in *Tyrannosaurus rex* using a three-dimensional musculoskeletal computer model: implications for stance, gait, and speed Paleobiology..

[58] Holliday CM, Ridgely RC, Sedlmayr JC, Witmer LM (2001). The articular cartilage of extant archosaur limb bones: implications for dinosaur functional morphology and allometry.. Journal of Vertebrate Paleontology.

[59] Bonnan M, Sandrik J, Nishiwaki T, Wilhite D, Elsey R Calcified cartilage shape in archosaur long bones reflect overlying joint shape in stress-bearing elements: implications for non-avian dinosaur locomotion.. Anat Rec.

[60] Witmer LM, Thomason J (1995). The extant phylogenetic bracket and the importance of reconstructing soft tissues in fossils.. Functional Morphology in Vertebrate Paleontology.

[61] Currey J, Alexander R (1985). The thickness of the walls of tubular bones.. J Zool.

[62] Ruff C, Damuth J, MacFadden B (1990). Body mass and hindlimb bone cross-sectional and articular dimensions in anthropoid primates.. Body Size in Mammalian Paleobiology: estimation and biological implications.

[63] Biknevicius A, Ruff C (1992). Use of biplanar radiographs for estimating cross-sectional geometric properties of mandibles.. Anat Rec.

[64] Heinrich R, Biknevicius A (1998). Skeletal allometry and interlimb scaling patterns in mustelid carnivorans.. J Morphol.

[65] Biknevicius A (1999). Body mass estimation in armoured mammals: cautions and encouragements for the use of parameters from the appendicular skeleton.. J Zool.

[66] Schmidt-Nielson K (1984). Scaling: Why is animal size so important?.

[67] Alexander R, Jayes A (1983). A dynamic similarity hypothesis for the gaits of quadrupedal mammals.. J Zool.

[68] Hutchinson JR, Garcia M (2002). *Tyrannosaurus* was not a fast runner.. Nature.

[69] Hutchinson JR (2004). Biomechanical modeling and sensitivity analysis of bipedal running ability. II. Extinct taxa.. J Morphol.

[70] Frey E (1988). Das Tragsystem der Krokodile—ein biomechanische und phylogenetische Analyse.. Stuttgarter Beiträge zur Naturkunde, Serie A (Biologie).

[71] Brochu CA (2001). Progress and future directions in archosaur phylogenetics.. Journal of Paleontology.

[72] Brochu CA (1999). Phylogeny, systematics, and historical biogeography of Alligatoroidea.. Society of Vertebrate Paleontology Memoir 6 J Vert Paleont.

[73] Baumel JJ Witmer LM, Baumel JJ (1993). Osteologia.. Handbook of Avian Anatomy: Nomina Anatomica Avium 2nd ed.

[74] Haines RW (1938). The primitive form of the epiphysis in the long bones of the tetrapods.. J Anat.

[75] Galton P (1970). The posture of hadrosaurian dinosaurs.. J Paleont.

[76] Charig A, Joysey K, Kemp T (1972). The evolution of the archosaur pelvis and hind-limb: an explanation in functional terms.. Studies in vertebrate evolution.

[77] Alexander RMcN (1985). Mechanics of posture and gait of some large dinosaurs.. Zool J Linn Soc.

[78] Sereno PC (1991). Basal archosaurs: phylogenetic relationships and functional implications.. J Vert Paleont.

[79] Sereno PC, Arcucci A (1993). Dinosaurian precursors from the middle Triassic of Argentina: *Lagerpeton chanarensis*.. J Vert Paleont.

[80] Dodson P (1996). The Horned Dinosaurs: a natural history..

[81] Wilson J, Carrano M (1999). Titanosaurs and the origin of “wide-gauge” trackways: a biomechanical and systematic perspective on sauropod locomotion.. Paleobiology.

[82] Hutchinson JR, Gatesy S (2000). Adductors, abductors, and the evolution of archosaurs locomotion.. Paleobiology.

[83] Carter DR, Mikic B, Padian K (1998). Epigenetic mechanical factors in the evolution of long bone epiphyses.. The Zoological Society of London.

[84] Carter D, Beaupré G (2001). Skeletal Form and Function..

[85] Carter DR Wong M (2003). Modeling cartilage mechanobiology.. Phil Trans Roy Soc London B.

[86] Carrano MT (2001). Implications of limb bone scaling, curvature and eccentricity in mammals and non-avian dinosaurs.. J Zool.

[87] Thulborn R, Gillette D, Lockley M (1989). The gaits of dinosaurs.. Dinosaur Tracks and Traces.

[88] Mazzetta G, Christiansen P, Farina R (2004). Body size of some southern South American Cretaceous dinosaurs.. Hist Biol.

[89] Royo-Torres R, Cobos A, Alcalá L (2006). A giant European dinosaur and a new sauropod clade.. Science.

[90] Alexander R (1991). How dinosaurs ran.. Sci Amer.

[91] Upchurch P, Barrett P, Dodson P, Weishampel D, Dodson P, Osmólska H (2004). Sauropoda.. The Dinosauria, 2nd edition.

[92] Hutchinson JR, Anderson FC, Blemker S, Delp SL (2005). Analysis of hindlimb muscle moment arms in *Tyrannosaurus rex* using a three-dimensional musculoskeletal computer model: implications for stance, gait, and speed.. Paleobiology.

[93] Heinrich R, Ruff C, Weishampel D (1993). Femoral ontogeny and locomotor biomechanics of Dryosaurus lettowvorbecki (Dinosauria, Iguanodontia).. Zool J Linn Soc.

[94] Holtz T (1994). The arctometatarsalian pes, an unusual structure of the metatarsus of Cretaceous Theropoda (Dinosauria: Saurischia).. J Vert Paleont.

[95] Gatesy S, Middleton K (1997). Bipedalism, flight, and the evolution of theropod locomotor diversity.. J Vert Paleont.

[96] Carrano MT (1998). Locomotion in non-avian dinosaurs: integrating data from hindlimb kinematics, in vivo strains, and bone morphology.. Paleobiology.

[97] Carrano MT (1999). What, if anything, is a cursor? Categories versus continua for determining locomotor habit in mammals and dinosaurs.. J Zool.

[98] Christiansen P (1999). Long bone scaling and limb posture in non-avian theropods: evidence for differential allometry.. J Vert Paleont.

[99] Blob RW (2000). Interspecific scaling of the hindlimb skeleton in lizards, crocodilians, felids and canids: does limb bone shape correlate with limb posture?. J Zool, Lond.

[100] Farlow J, Gatesy S, Holtz TJ, Hutchinson J, Robinson J (2000). Theropod locomotion.. Amer Zool.

[101] Farlow JO, Elsey RM (2004). Femoral dimensions and mid-thigh circumference in *Alligator mississippiensis*.. Lethaia.

[102] Senter P (2007). Anaylsis of forelimb function in basal ceratopsians.. Journal of Zoology.

[103] Faux CM, Padian K (2007). The opisthotonic posture of vertebrate skeletons: postmortem contraction or death throes?. Paleobiol.

[104] Carpenter K, Farlow J, Brett-Surman M (1997). Dinosaurs as Museum Exhibits.. The Complete Dinosaur.

[105] Chatterjee S (1985). *Postosuchus*, a new thecodontian reptile from the Triassic of Texas and the origin of tyrannosaurs.. Phil Trans Roy Soc London B.

[106] Colbert E (1947). Studies of the phytosaurs, *Machaeroprosopus* and *Rutiodon*.. Bull Amer Mus Nat Hist.

[107] Long R, Murray R (1995). Late Triassic (Carnian and Norian) tetrapods from the Southwestern United States.. Bull New Mex Mus Nat Hist.

[108] Galton P, Upchurch P, Weishampel D, Dodson P, Osmólska H (2004). Prosauropoda.. The Dinosauria, 2nd edition.

[109] Colbert E (1989). The Triassic dinosaur *Coelophysis*.. Mus Nor Ariz Bull.

[110] Ostrom J, McIntosh J (1966). Marsh's Dinosaurs: the collections from Como Bluff..

[111] Marsh OC (1883). Principle characters of American Jurassic dinosaurs. Part VI. Restoration of *Brontosaurous*.. American Journal of Science.

[112] Cope ED (1878). On the Saurians recently discovered in the Dakota Beds of Colorado.. American Naturalist.

[113] Madsen JJ (1976). *Allosaurus fragilis*: a revised osteology.. Utah Geological Survey Bulletin.

[114] Ostrom J (1976). On a new specimen of the lower Cretaceous theropod dinosaur *Deinonychus antirrhopus*.. Brevoria.

[115] Starck JM, Chinsamy A (2002). Bone Microstructure and Developmental Plasticity in Birds and Other Dinosaurs.. J Morphol.

[116] Erickson GM, Rogers KC, Yerby SA (2001). Dinosaurian growth patterns and rapid avian growth rates.. Nature.

[117] Padian K, de Ricqlés A, Horner JR (2001). Dinosaurian growth rates and bird origins.. Nature.

[118] Curry-Rogers K, Erickson G, Rogers KC, Wilson J (2005). Sauropod histology: microscopic views on the lives of giants.. The Sauropods: evolution and paleobiology.

[119] Erickson G (2005). Assessing dinosaur growth patterns: a microscopic revolution.. TREE.

[120] Jaramillo D, Villegas-Medina O, Doty D, Rivas R, Strife K (2004). Age-related vascular changes in the epiphysis, physis, and methaphysis: normal findings on gadolinium-enhanced MRI of piglets.. Amer J Roentgen.

[121] Brown B Schlaikjer EM (1940). The structure and relationships of *Protoceratops*.. Ann New York Acad Sci.

[122] Lull RS Wright NE (1942). Hadrosaurian dinosaurs of North America.. Geol Soc Am Spec Pap.

