# Supplementary material for: Cartilaginous Epiphyses in Extant Archosaurs and Their Implications for Reconstructing Limb Function in Dinosaurs
Source: PLoS One. 2010 Sep 30;5(9):e13120. doi: 10.1371/journal.pone.0013120 (PMC2948032; doi:10.1371/journal.pone.0013120)
Supplement: File S1 — Holliday et al. 2010 PLOS ONE Archosaur Epiphyses. (1.29 MB DOC) [file pone.0013120.s001.doc]

**Supporting Information (S1)**

**Holliday et al. 2010 PLOS ONE Archosaur Epiphyses**

**Table 1:** List of Archosaur specimens and relevant measurements used in study including disposition and body mass where available. All extant specimens are housed in the Ohio University Vertebrate Collections (**OUVC)**. Body masses are in grams (g). Femoral midshaft cross-sectional area in (mm). All other measurements are in (cm). Pgs 2-16: *Alligator*; Pgs 17-24: Birds (Ostrich, Quail, Chicken, Duck). Percent change in greatest length (GL) is reported for total length only in avian femora. Greatest lengths of avian non-femoral elements are listed under Condyle-trochanter for sake of space, however these measurements are GL measurements as described in the text.

|  | | | |  |
| --- | --- | --- | --- | --- |
| **OUVC** | ***Taxon*** | **Disposition** | **Body Mass (g)** | **Femoral cross-sectional area (mm)** |
| **9401** | *Alligator mississippiensis* | Trunk only | UK | 572.71 |
| **9402** | *Alligator mississippiensis* | whole specimen | UK | 34.55 |
| **9403** | *Alligator mississippiensis* | whole specimen | 16400 | 286.98 |
| **9404** | *Alligator mississippiensis* | whole specimen | 6900 | 156.62 |
| **9405** | *Alligator mississippiensis* | whole specimen | 6400 | 178.07 |
| **9406** | *Alligator mississippiensis* | whole specimen | 3110 | 79.02 |
| **9407** | *Alligator mississippiensis* | Trunk only | UK | 391.84 |
| **9408** | *Alligator mississippiensis* | whole specimen | 2900 | 130.53 |
| **9409** | *Alligator mississippiensis* | whole specimen | 3200 | 90.54 |
| **9410** | *Alligator mississippiensis* | whole specimen | 3000 | 137.66 |
| **9411** | *Alligator mississippiensis* | whole specimen | UK | 877.75 |
| **9412** | *Alligator mississippiensis* | whole specimen | UK | 815.93 |
| **9413** | *Alligator mississippiensis* | whole specimen | UK | 625.66 |
| **9414** | *Alligator mississippiensis* | whole specimen | UK | 412.95 |
| **9415** | *Alligator mississippiensis* | whole specimen | UK | 572.71 |
| **9416** | *Coturnix japonica* | whole specimen | 78 |  |
| **9417** | *Coturnix japonica* | whole specimen | UK |  |
| **9418** | *Coturnix japonica* | whole specimen | 136 |  |
| **9419** | *Gallus gallus* | whole specimen | 2110 |  |
| **9420** | *Gallus gallus* | whole specimen | 2450 |  |
| **9421** | *Aix sponsa* | whole specimen | UK |  |
| **9422** | *Struthio camelus* | whole specimen | 89810 |  |
| **9423** | *Struthio camelus* | whole specimen | 86180 |  |
| **9424** | *Struthio camelus* | hindlimbs and humeri | UK |  |
| **9425** | *Struthio camelus* | hindlimbs and humeri | UK |  |
| **9426** | *Struthio camelus* | hindlimbs and humeri | UK |  |
| **9427** | *Struthio camelus* | hindlimbs and humeri | UK |  |
| **9428** | *Struthio camelus* | hindlimbs and humeri | UK |  |
| **9429** | *Struthio camelus* | hindlimbs and humeri | UK |  |
| **9430** | *Struthio camelus* | hindlimbs and humeri | UK |  |
| **9431** | *Struthio camelus* | hindlimbs and humeri | UK |  |
| **9432** | *Struthio camelus* | hindlimbs and humeri | UK |  |
| **9433** | *Struthio camelus* | hindlimbs and humeri | UK |  |
| **9434** | *Struthio camelus* | femur, L. | UK |  |
| **9435** | *Struthio camelus* | femur, L. | UK |  |
| **9436** | *Struthio camelus* | femur, L. | UK |  |
| **9437** | *Struthio camelus* | femur, R. | UK |  |
| **9438** | *Struthio camelus* | femur, R. | UK |  |
| **9439** | *Struthio camelus* | femur, R. | UK |  |

| ***Alligator mississippiensis*** | | | | **Proximal** | | | | | | **Distal** | | | | | |
| --- | --- | --- | --- | --- | --- | --- | --- | --- | --- | --- | --- | --- | --- | --- | --- |
| **OUVC 9401** | **GreatestLength** | **w/o cart** | **Percent change** | **Craniocaudal** | **w/o cart.** | **Percent change** | **Medio**  **lateral** | **w/o cart.** | **Percent change** | **Craniocaudal** | **w/o cart.** | **Percent change** | **Medio**  **lateral** | **w/o cart.** | **Percent change** |
| **L. Femur** | 133.40 | 128.35 | 3.79 | 17.45 | 14.03 | 19.60 | 33.32 | 29.90 | 10.26 | 23.67 | 19.75 | 16.56 | 33.00 | 30.18 | 8.55 |
| **R. Femur** | 133.33 | 128.00 | 4.00 | 17.42 | 14.62 | 16.07 | 33.30 | 29.95 | 10.06 | 24.00 | 19.80 | 17.50 | 32.92 | 30.40 | 7.65 |
| **Mean** | 133.37 | 128.18 | 3.89 | 17.43 | 14.33 | 17.79 | 33.31 | 29.93 | 10.15 | 23.83 | 19.78 | 17.00 | 32.96 | 30.29 | 8.10 |
| **L. Tibia** | 102.54 | 101.30 | 1.21 | 27.73 | 25.28 | 8.84 | 28.08 | 24.48 | 12.82 | 9.98 | 6.67 | 33.17 | 25.83 | 24.03 | 6.97 |
| **R. Tibia** | 103.17 | 101.22 | 1.89 | 27.55 | 25.32 | 8.09 | 28.65 | 24.40 | 14.83 | 10.32 | 6.58 | 36.24 | 25.70 | 24.05 | 6.42 |
| **Mean** | 102.86 | 101.26 | 1.56 | 27.64 | 25.30 | 8.47 | 28.37 | 24.44 | 13.85 | 10.15 | 6.63 | 34.68 | 25.77 | 24.04 | 6.71 |
| **L. Fibula** | 104.45 | 97.97 | 6.20 | 5.90 | 4.68 | 20.68 | 14.62 | 13.07 | 10.60 | 15.19 | 12.12 | 20.21 | 12.53 | 10.47 | 16.44 |
| **R. Fibula** | 103.15 | 99.20 | 3.83 | 5.53 | 4.70 | 15.01 | 14.37 | 13.08 | 8.98 | 14.70 | 12.12 | 17.55 | 12.62 | 10.47 | 17.04 |
| **Mean** | 103.80 | 98.58 | 5.03 | 5.72 | 4.69 | 18.01 | 14.49 | 13.08 | 9.73 | 14.95 | 12.12 | 18.93 | 12.58 | 10.47 | 16.77 |
| **L. Humerus** | 121.32 | 115.70 | 4.63 | 10.45 | 7.70 | 26.32 | 30.67 | 30.45 | 0.72 | 20.82 | 15.05 | 27.71 | 31.23 | 27.05 | 13.38 |
| **R. Humerus** | 120.15 | 115.77 | 3.65 | 10.53 | 7.92 | 24.79 | 32.07 | 30.07 | 6.24 | 19.98 | 15.13 | 24.27 | 30.90 | 27.10 | 12.30 |
| **Mean** | 120.65 | 115.73 | 4.08 | 10.49 | 7.81 | 25.55 | 31.36 | 30.26 | 3.51 | 20.22 | 15.09 | 25.37 | 31.05 | 27.08 | 12.79 |
| **L. Ulna** | 91.77 | 85.80 | 6.51 | 19.60 | 17.03 | 13.11 | 21.58 | 19.37 | 10.24 | 9.33 | 7.30 | 21.76 | 16.05 | 12.92 | 19.50 |
| **R. Ulna** | 91.45 | 85.45 | 6.56 | 19.44 | 17.10 | 12.04 | 21.97 | 18.92 | 13.88 | 9.42 | 7.43 | 21.13 | 15.42 | 12.58 | 18.42 |
| **Mean** | 91.73 | 85.60 | 6.68 | 19.57 | 17.05 | 12.88 | 21.66 | 19.16 | 11.54 | 9.38 | 7.37 | 21.43 | 15.73 | 12.75 | 18.94 |
| **L. Radius** | 81.25 | 77.80 | 4.25 | 12.45 | 10.65 | 14.46 | 16.78 | 14.80 | 11.80 | 9.17 | 6.97 | 23.99 | 18.95 | 15.68 | 17.26 |
| **R. Radius** | 82.32 | 74.83 | 9.10 | 12.27 | 10.63 | 13.37 | 16.50 | 14.35 | 13.03 | 9.23 | 6.93 | 24.92 | 18.57 | 16.03 | 13.68 |
| **Mean** | 81.81 | 76.32 | 6.71 | 12.33 | 10.64 | 13.71 | 16.63 | 14.58 | 12.33 | 9.14 | 6.95 | 23.96 | 18.83 | 15.86 | 15.77 |

| ***Alligator mississippiensis*** | | | | **Proximal** | | | | | | **Distal** | | | | | |
| --- | --- | --- | --- | --- | --- | --- | --- | --- | --- | --- | --- | --- | --- | --- | --- |
| **OUVC 9402** | **GreatestLength** | **w/o cart** | **Percent change** | **Craniocaudal** | **w/o cart.** | **Percent change** | **Medio**  **lateral** | **w/o cart.** | **Percent change** | **Craniocaudal** | **w/o cart.** | **Percent change** | **Medio**  **lateral** | **w/o cart.** | **Percent change** |
| **L. Femur** | 50.85 | 47.00 | 7.57 | 5.52 | 4.57 | 17.21 | 10.73 | 9.47 | 11.74 | 5.52 | 4.57 | 17.21 | 10.48 | 9.33 | 10.97 |
| **R. Femur** | 50.83 | 47.03 | 7.48 | 5.68 | 4.57 | 19.54 | 10.73 | 9.43 | 12.12 | 5.68 | 4.57 | 19.54 | 10.48 | 9.37 | 10.59 |
| **Mean** | 50.84 | 47.02 | 7.51 | 5.60 | 4.57 | 18.39 | 10.73 | 9.45 | 11.93 | 5.60 | 4.57 | 18.39 | 10.48 | 9.35 | 10.78 |
| **L. Tibia** | 41.42 | 37.78 | 8.79 | 41.42 | 37.78 | 8.79 | 9.80 | 8.37 | 14.59 | 8.35 | 7.23 | 13.41 | 9.22 | 8.50 | 7.81 |
| **R. Tibia** | 41.40 | 37.83 | 8.62 | 41.40 | 37.83 | 8.62 | 9.80 | 8.28 | 15.51 | 8.35 | 7.23 | 13.41 | 9.23 | 8.47 | 8.23 |
| **Mean** | 41.41 | 37.81 | 8.69 | 41.41 | 37.81 | 8.69 | 9.80 | 8.33 | 15.00 | 8.35 | 7.23 | 13.41 | 9.23 | 8.48 | 8.13 |
| **L. Fibula** | 39.48 | 37.58 | 4.81 | 39.48 | 37.58 | 4.81 | 5.52 | 4.32 | 21.74 | 2.43 | 1.80 | 25.93 | 4.17 | 3.23 | 22.54 |
| **R. Fibula** | 39.48 | 37.58 | 4.81 | 39.48 | 37.58 | 4.81 | 5.50 | 4.33 | 21.27 | 2.42 | 1.78 | 26.45 | 4.18 | 3.23 | 22.73 |
| **Mean** | 39.48 | 37.58 | 4.81 | 39.48 | 37.58 | 4.81 | 5.51 | 4.33 | 21.42 | 2.43 | 1.79 | 26.34 | 4.18 | 3.23 | 22.73 |
| **L. Humerus** | 45.45 | 39.15 | 13.86 | 45.45 | 39.15 | 13.86 | 11.25 | 9.80 | 12.89 | 5.28 | 3.90 | 26.14 | 10.78 | 9.38 | 12.99 |
| **R. Humerus** | 45.22 | 39.12 | 13.49 | 45.22 | 39.12 | 13.49 | 11.20 | 9.80 | 12.50 | 5.30 | 3.88 | 26.79 | 10.82 | 9.42 | 12.94 |
| **Mean** | 45.33 | 39.13 | 13.68 | 45.33 | 39.13 | 13.68 | 11.23 | 9.80 | 12.73 | 5.29 | 3.89 | 26.47 | 10.80 | 9.40 | 12.96 |
| **L. Ulna** | 35.48 | 30.45 | 14.18 | 35.48 | 30.45 | 14.18 | 6.85 | 6.05 | 11.68 | 7.45 | 5.33 | 28.46 | 5.50 | 4.47 | 18.73 |
| **R. Ulna** | 35.50 | 30.43 | 14.28 | 35.50 | 30.43 | 14.28 | 6.87 | 6.02 | 12.37 | 7.45 | 5.37 | 27.92 | 5.50 | 4.47 | 18.73 |
| **Mean** | 35.49 | 30.44 | 14.23 | 35.49 | 30.44 | 14.23 | 6.86 | 6.03 | 12.10 | 7.45 | 5.35 | 28.19 | 5.50 | 4.47 | 18.73 |
| **L. Radius** | 32.60 | 30.20 | 7.36 | 32.60 | 30.20 | 7.36 | 5.97 | 5.03 | 15.75 | 4.22 | 3.07 | 27.25 | 6.15 | 5.33 | 13.33 |
| **R. Radius** | 32.58 | 30.22 | 7.24 | 32.58 | 30.22 | 7.24 | 5.95 | 5.00 | 15.97 | 4.20 | 3.07 | 26.90 | 6.17 | 5.33 | 13.61 |
| **Mean** | 32.59 | 30.21 | 7.30 | 32.59 | 30.21 | 7.30 | 5.96 | 5.02 | 15.77 | 4.21 | 3.07 | 27.08 | 6.16 | 5.33 | 13.47 |

| ***Alligator mississippiensis*** | | | | **Proximal** | | | | | | **Distal** | | | | | |
| --- | --- | --- | --- | --- | --- | --- | --- | --- | --- | --- | --- | --- | --- | --- | --- |
| **OUVC 9403** | **GreatestLength** | **w/o cart** | **Percent change** | **Craniocaudal** | **w/o cart.** | **Percent change** | **Medio**  **lateral** | **w/o cart.** | **Percent change** | **Craniocaudal** | **w/o cart.** | **Percent change** | **Medio**  **lateral** | **w/o cart.** | **Percent change** |
| **L. Femur** | 113.32 | 106.45 | 6.06 | 16.75 | 12.92 | 22.87 | 30.50 | 26.67 | 12.56 | 19.61 | 17.07 | 12.95 | 29.12 | 26.13 | 10.27 |
| **R. Femur** | 114.57 | 106.50 | 7.04 | 16.97 | 12.95 | 23.69 | 30.35 | 26.68 | 12.09 | 23.00 | 19.28 | 16.17 | 28.57 | 26.22 | 8.23 |
| **Mean** | 113.94 | 106.48 | 6.55 | 16.86 | 12.93 | 23.31 | 30.43 | 26.68 | 12.32 | 21.31 | 18.18 | 14.69 | 28.84 | 26.18 | 9.22 |
| **L. Tibia** | 86.62 | 79.00 | 8.80 | 24.48 | 19.78 | 19.20 | 25.30 | 21.65 | 14.43 | 7.17 | 5.75 | 19.80 | 24.20 | 21.00 | 13.22 |
| **R. Tibia** | 88.58 | 78.93 | 10.89 | 25.13 | 21.70 | 13.65 | 24.97 | 19.45 | 22.11 | 7.32 | 5.85 | 20.08 | 24.12 | 21.08 | 12.60 |
| **Mean** | 87.53 | 78.93 | 9.83 | 24.82 | 20.74 | 16.44 | 25.15 | 20.55 | 18.29 | 7.23 | 5.80 | 19.78 | 24.13 | 21.03 | 12.85 |
| **L. Fibula** | 88.23 | 82.10 | 6.95 | 6.38 | 5.15 | 19.28 | 15.10 | 12.38 | 18.01 | 15.38 | 11.83 | 23.08 | 11.37 | 9.15 | 19.53 |
| **R. Fibula** | 86.63 | 82.10 | 5.23 | 6.47 | 5.20 | 19.63 | 15.12 | 12.60 | 16.67 | 15.30 | 11.80 | 22.88 | 11.43 | 9.25 | 19.07 |
| **Mean** | 87.39 | 82.10 | 6.05 | 6.44 | 5.18 | 19.57 | 15.13 | 12.49 | 17.45 | 15.32 | 11.82 | 22.85 | 11.43 | 9.20 | 19.51 |
| **L. Humerus** | 108.93 | 99.60 | 8.57 | 15.27 | 14.60 | 4.39 | 24.25 | 23.07 | 4.87 | 19.72 | 16.55 | 16.08 | 27.02 | 26.42 | 2.22 |
| **R. Humerus** | 108.38 | 98.40 | 9.21 | 16.32 | 11.25 | 31.07 | 25.72 | 23.13 | 10.07 | 18.47 | 16.90 | 8.50 | 27.33 | 27.10 | 0.84 |
| **Mean** | 108.69 | 99.00 | 8.92 | 15.76 | 12.93 | 17.96 | 25.01 | 23.07 | 7.76 | 19.11 | 16.73 | 12.45 | 26.82 | 27.05 | -0.86 |
| **L. Ulna** | 84.23 | 73.68 | 12.53 | 23.50 | 18.80 | 20.00 | 24.10 | 17.70 | 26.56 | 6.07 | 5.57 | 8.24 | 16.43 | 11.27 | 31.41 |
| **R. Ulna** | 82.48 | 72.57 | 12.02 | 22.57 | 18.17 | 19.49 | 24.38 | 17.82 | 26.91 | 7.68 | 5.32 | 30.73 | 15.85 | 11.70 | 26.18 |
| **Mean** | 83.34 | 73.11 | 12.28 | 23.08 | 18.48 | 19.93 | 24.24 | 17.76 | 26.73 | 6.82 | 5.43 | 20.38 | 16.18 | 11.48 | 29.05 |
| **L. Radius** | 70.83 | 64.00 | 9.64 | 12.80 | 11.10 | 13.28 | 19.22 | 14.03 | 27.00 | 6.58 | 6.50 | 1.22 | 15.85 | 6.58 | 58.49 |
| **R. Radius** | 70.77 | 64.00 | 9.57 | 12.62 | 11.00 | 12.84 | 19.18 | 14.50 | 24.40 | 6.50 | 6.25 | 3.85 | 16.20 | 6.50 | 59.88 |
| **Mean** | 70.93 | 64.00 | 9.77 | 12.66 | 11.05 | 12.72 | 19.09 | 14.28 | 25.20 | 6.60 | 6.38 | 3.33 | 16.03 | 6.60 | 58.83 |

| ***Alligator mississippiensis*** | | | | **Proximal** | | | | | | **Distal** | | | | | |
| --- | --- | --- | --- | --- | --- | --- | --- | --- | --- | --- | --- | --- | --- | --- | --- |
| **OUVC 9404** | **GreatestLength** | **w/o cart** | **Percent change** | **Craniocaudal** | **w/o cart.** | **Percent change** | **Medio**  **lateral** | **w/o cart.** | **Percent change** | **Craniocaudal** | **w/o cart.** | **Percent change** | **Medio**  **lateral** | **w/o cart.** | **Percent change** |
| **L. Femur** | 86.35 | 79.02 | 8.49 | 11.38 | 7.83 | 31.20 | 21.02 | 16.58 | 21.12 | 8.97 | 8.97 | 0.00 | 19.33 | 17.57 | 9.11 |
| **R. Femur** | 86.77 | 79.03 | 8.92 | 11.43 | 7.92 | 30.71 | 20.80 | 16.63 | 20.05 | 10.90 | 10.02 | 8.07 | 19.35 | 17.62 | 8.94 |
| **Mean** | 86.56 | 79.03 | 8.70 | 11.41 | 7.88 | 30.94 | 20.91 | 16.61 | 20.56 | 9.93 | 9.49 | 4.43 | 19.34 | 17.59 | 9.05 |
| **L. Tibia** | 67.77 | 62.48 | 7.81 | 17.92 | 12.55 | 29.97 | 18.02 | 14.97 | 16.93 | 8.98 | 5.52 | 38.53 | 15.08 | 12.88 | 14.59 |
| **R. Tibia** | 67.47 | 62.48 | 7.40 | 17.77 | 12.50 | 29.66 | 18.07 | 14.92 | 17.43 | 8.62 | 5.52 | 35.96 | 15.10 | 12.87 | 14.77 |
| **Mean** | 67.58 | 62.49 | 7.53 | 17.78 | 12.53 | 29.53 | 18.06 | 14.93 | 17.33 | 8.82 | 5.51 | 37.53 | 15.09 | 12.89 | 14.58 |
| **L. Fibula** | 64.73 | 61.48 | 5.02 | 4.30 | 2.67 | 37.91 | 10.40 | 7.60 | 26.92 | 10.15 | 6.43 | 36.65 | 8.30 | 7.48 | 9.88 |
| **R. Fibula** | 64.65 | 61.48 | 4.90 | 4.45 | 2.63 | 40.90 | 10.47 | 7.57 | 27.70 | 10.28 | 6.42 | 37.55 | 8.32 | 7.45 | 10.46 |
| **Mean** | 64.69 | 61.47 | 4.98 | 4.38 | 2.63 | 39.95 | 10.50 | 7.60 | 27.62 | 10.20 | 6.44 | 36.86 | 8.33 | 7.47 | 10.32 |
| **L. Humerus** | 78.97 | 70.50 | 10.73 | 7.57 | 4.52 | 40.29 | 16.50 | 11.58 | 29.82 | 11.55 | 8.92 | 22.77 | 15.70 | 15.52 | 1.15 |
| **R. Humerus** | 79.62 | 70.47 | 11.49 | 7.10 | 4.52 | 36.34 | 15.13 | 11.60 | 23.33 | 11.50 | 8.92 | 22.43 | 15.37 | 15.00 | 2.41 |
| **Mean** | 79.33 | 70.48 | 11.16 | 7.38 | 4.51 | 38.89 | 15.82 | 11.60 | 26.68 | 11.45 | 8.91 | 22.18 | 15.43 | 15.25 | 1.17 |
| **L. Ulna** | 60.72 | 53.82 | 11.36 | 14.17 | 10.03 | 29.22 | 15.27 | 10.92 | 28.49 | 5.85 | 4.60 | 21.37 | 9.27 | 7.93 | 14.46 |
| **R. Ulna** | 60.88 | 53.55 | 12.04 | 14.10 | 10.02 | 28.94 | 15.28 | 10.87 | 28.86 | 5.40 | 4.60 | 14.81 | 9.60 | 7.93 | 17.40 |
| **Mean** | 60.82 | 53.68 | 11.74 | 14.10 | 10.03 | 28.87 | 15.29 | 10.88 | 28.84 | 5.63 | 4.60 | 18.29 | 9.40 | 7.92 | 15.74 |
| **L. Radius** | 56.23 | 49.17 | 12.56 | 9.93 | 6.32 | 36.35 | 11.95 | 8.87 | 25.77 | 6.78 | 4.43 | 34.66 | 11.90 | 8.92 | 25.04 |
| **R. Radius** | 55.05 | 49.20 | 10.63 | 9.87 | 6.33 | 35.87 | 11.82 | 8.88 | 24.87 | 5.22 | 4.48 | 14.18 | 12.12 | 8.92 | 26.40 |
| **Mean** | 55.63 | 49.20 | 11.56 | 9.91 | 6.34 | 36.02 | 11.88 | 8.89 | 25.17 | 6.01 | 4.47 | 25.62 | 12.06 | 8.91 | 26.12 |

| ***Alligator mississippiensis*** | | | | **Proximal** | | | | | | **Distal** | | | | | |
| --- | --- | --- | --- | --- | --- | --- | --- | --- | --- | --- | --- | --- | --- | --- | --- |
| **OUVC 9405** | **GreatestLength** | **w/o cart** | **Percent change** | **Craniocaudal** | **w/o cart.** | **Percent change** | **Medio**  **lateral** | **w/o cart.** | **Percent change** | **Craniocaudal** | **w/o cart.** | **Percent change** | **Medio**  **lateral** | **w/o cart.** | **Percent change** |
| **L. Femur** | 89.73 | 83.38 | 7.08 | 10.10 | 8.02 | 20.59 | 20.65 | 17.17 | 16.85 | 13.15 | 9.95 | 24.33 | 19.17 | 17.42 | 9.13 |
| **R. Femur** | 89.72 | 83.40 | 7.04 | 9.85 | 8.05 | 18.27 | 20.65 | 17.15 | 16.95 | 14.07 | 10.70 | 23.95 | 19.13 | 17.40 | 9.04 |
| **Mean** | 89.73 | 83.39 | 7.07 | 9.98 | 8.03 | 19.54 | 20.65 | 17.16 | 16.90 | 13.61 | 10.33 | 24.10 | 19.15 | 17.41 | 9.09 |
| **L. Tibia** | 69.55 | 66.07 | 5.00 | 17.02 | 12.48 | 26.67 | 17.65 | 14.30 | 18.98 | 5.33 | 4.50 | 15.57 | 16.68 | 14.45 | 13.37 |
| **R. Tibia** | 69.52 | 66.10 | 4.92 | 17.23 | 12.47 | 27.63 | 17.80 | 14.30 | 19.66 | 5.33 | 4.50 | 15.57 | 16.75 | 14.45 | 13.73 |
| **Mean** | 69.53 | 66.08 | 4.96 | 17.13 | 12.49 | 27.09 | 17.73 | 14.30 | 19.35 | 5.32 | 4.50 | 15.41 | 16.72 | 14.45 | 13.58 |
| **L. Fibula** | 69.93 | 64.43 | 7.87 | 4.27 | 2.85 | 33.26 | 9.87 | 7.50 | 24.01 | 10.30 | 8.12 | 21.17 | 7.85 | 6.05 | 22.93 |
| **R. Fibula** | 71.35 | 64.43 | 9.70 | 4.30 | 2.85 | 33.72 | 10.97 | 7.50 | 31.63 | 10.40 | 8.10 | 22.12 | 7.75 | 6.05 | 21.94 |
| **Mean** | 70.67 | 64.44 | 8.82 | 4.28 | 2.85 | 33.41 | 10.41 | 7.50 | 27.95 | 10.35 | 8.11 | 21.64 | 7.80 | 6.05 | 22.44 |
| **L. Humerus** | 83.55 | 75.70 | 9.40 | 7.57 | 4.30 | 43.20 | 18.37 | 17.10 | 6.91 | 11.95 | 8.60 | 28.03 | 18.07 | 16.20 | 10.35 |
| **R. Humerus** | 83.48 | 75.72 | 9.30 | 7.52 | 4.33 | 42.42 | 18.25 | 17.10 | 6.30 | 12.20 | 8.60 | 29.51 | 18.10 | 16.22 | 10.39 |
| **Mean** | 83.52 | 75.71 | 9.35 | 7.51 | 4.32 | 42.48 | 18.28 | 17.10 | 6.46 | 12.08 | 8.60 | 28.81 | 18.10 | 16.21 | 10.44 |
| **L. Ulna** | 62.57 | 56.80 | 9.22 | 13.63 | 9.40 | 31.03 | 12.73 | 11.45 | 10.05 | 6.20 | 4.20 | 32.26 | 10.28 | 8.00 | 22.18 |
| **R. Ulna** | 62.75 | 56.78 | 9.51 | 13.75 | 9.42 | 31.49 | 12.78 | 11.50 | 10.02 | 6.18 | 4.23 | 31.55 | 10.22 | 8.02 | 21.53 |
| **Mean** | 62.68 | 56.79 | 9.40 | 13.68 | 9.41 | 31.21 | 12.74 | 11.48 | 9.89 | 6.19 | 4.22 | 31.83 | 10.23 | 8.01 | 21.70 |
| **L. Radius** | 55.53 | 51.90 | 6.54 | 9.05 | 7.25 | 19.89 | 10.90 | 8.32 | 23.67 | 6.52 | 4.20 | 35.58 | 12.80 | 9.60 | 25.00 |
| **R. Radius** | — | — | — | — | — | — | — | — | — | — | — | ! | — | — |  |
| **Mean** | 55.53 | 51.90 | 6.54 | 9.05 | 7.25 | 19.89 | 10.90 | 8.32 | 23.67 | 6.52 | 4.20 | 35.58 | 12.80 | 9.60 | 25.00 |

| ***Alligator mississippiensis*** | | | | **Proximal** | | | | | | **Distal** | | | | | |
| --- | --- | --- | --- | --- | --- | --- | --- | --- | --- | --- | --- | --- | --- | --- | --- |
| **OUVC 9406** | **GreatestLength** | **w/o cart** | **Percent change** | **Craniocaudal** | **w/o cart.** | **Percent change** | **Medio**  **lateral** | **w/o cart.** | **Percent change** | **Craniocaudal** | **w/o cart.** | **Percent change** | **Medio**  **lateral** | **w/o cart.** | **Percent change** |
| **L. Femur** | 67.80 | 62.40 | 7.96 | 7.52 | 7.00 | 6.91 | 15.72 | 13.50 | 14.12 | 10.40 | 9.20 | 11.54 | 15.05 | 14.20 | 5.65 |
| **R. Femur** | 68.18 | 62.35 | 8.55 | 7.52 | 6.95 | 7.58 | 15.70 | 13.48 | 14.14 | 10.22 | 9.20 | 9.98 | 14.75 | 13.82 | 6.31 |
| **Mean** | 67.83 | 62.40 | 8.01 | 7.52 | 6.98 | 7.18 | 15.71 | 13.49 | 14.13 | 10.31 | 9.20 | 10.77 | 14.90 | 14.01 | 5.97 |
| **L. Tibia** | 57.05 | 54.15 | 5.08 | 13.13 | 10.83 | 17.52 | 13.40 | 11.10 | 17.16 | 5.18 | 4.40 | 15.06 | 12.75 | 11.10 | 12.94 |
| **R. Tibia** | 57.18 | 54.28 | 5.07 | 13.12 | 11.00 | 16.16 | 13.50 | 10.97 | 18.74 | 5.13 | 4.38 | 14.62 | 12.80 | 11.30 | 11.72 |
| **Mean** | 57.10 | 54.15 | 5.17 | 13.13 | 10.92 | 16.83 | 13.45 | 11.03 | 17.99 | 5.16 | 4.39 | 14.92 | 12.78 | 11.20 | 12.36 |
| **L. Fibula** | 56.07 | 51.10 | 8.86 | 7.23 | 6.20 | 14.25 | 7.23 | 6.20 | 14.25 | 7.70 | 5.90 | 23.38 | 6.02 | 5.05 | 16.11 |
| **R. Fibula** | — | — | — | 7.25 | 6.30 | 13.10 | 7.25 | 6.30 | 13.10 | — | — | — | — | — |  |
| **Mean** | 56.07 | 51.10 | 8.86 | 7.24 | 6.25 | 13.67 | 7.24 | 6.25 | 13.67 | 7.70 | 5.90 | 23.38 | 6.02 | 5.05 | 16.11 |
| **L. Humerus** | 61.70 | 55.85 | 9.48 | 13.52 | 13.18 | 2.51 | 13.52 | 13.18 | 2.51 | 4.50 | 4.00 | 11.11 | 12.83 | 11.01 | 14.19 |
| **R. Humerus** | 61.70 | 55.85 | 9.48 | 13.53 | 13.20 | 2.44 | 13.53 | 13.20 | 2.44 | 4.40 | 3.89 | 11.59 | 12.68 | 11.10 | 12.46 |
| **Mean** | 61.70 | 55.85 | 9.48 | 13.53 | 13.19 | 2.51 | 13.53 | 13.19 | 2.51 | 4.45 | 3.95 | 11.24 | 12.76 | 11.05 | 13.40 |
| **L. Ulna** | 49.00 | 43.90 | 10.41 | 10.50 | 8.55 | 18.57 | 11.38 | 8.55 | 24.87 | 4.45 | 2.48 | 44.27 | 7.68 | 6.85 | 10.81 |
| **R. Ulna** | 49.10 | 44.15 | 10.08 | 10.48 | 8.55 | 18.42 | — | — | — | 4.50 | 2.50 | 44.44 | 7.75 | 6.82 | 12.00 |
| **Mean** | 49.03 | 44.12 | 10.01 | 10.49 | 8.55 | 18.49 | 11.38 | 8.55 | 24.87 | 4.48 | 2.49 | 44.42 | 7.72 | 6.83 | 11.53 |
| **L. Radius** | 46.10 | 40.40 | 12.36 | 8.83 | 7.15 | 19.03 | 8.83 | 7.15 | 19.03 | 9.65 | 7.62 | 21.04 | 9.65 | 7.62 | 21.04 |
| **R. Radius** | 46.05 | 40.15 | 12.81 | 8.80 | 7.20 | 18.18 | 8.80 | 7.20 | 18.18 | 9.70 | 7.95 | 18.04 | 9.70 | 7.95 | 18.04 |
| **Mean** | 46.08 | 40.15 | 12.87 | 8.80 | 7.20 | 18.18 | 8.80 | 7.20 | 18.18 | 9.68 | 7.95 | 17.87 | 9.68 | 7.95 | 17.87 |

| ***Alligator mississippiensis*** | | | | **Proximal** | | | | | | **Distal** | | | | | |
| --- | --- | --- | --- | --- | --- | --- | --- | --- | --- | --- | --- | --- | --- | --- | --- |
| **OUVC 9407** | **GreatestLength** | **w/o cart** | **Percent change** | **Craniocaudal** | **w/o cart.** | **Percent change** | **Medio**  **lateral** | **w/o cart.** | **Percent change** | **Craniocaudal** | **w/o cart.** | **Percent change** | **Medio**  **lateral** | **w/o cart.** | **Percent change** |
| **L. Femur** | 67.80 | 62.40 | 7.96 | 7.52 | 7.00 | 6.91 | 15.72 | 13.50 | 14.12 | 10.40 | 9.20 | 11.54 | 15.05 | 14.20 | 5.65 |
| **R. Femur** | 68.18 | 62.35 | 8.55 | 7.52 | 6.95 | 7.58 | 15.70 | 13.48 | 14.14 | 10.22 | 9.20 | 9.98 | 14.75 | 13.82 | 6.31 |
| **Mean** | 67.83 | 62.40 | 8.01 | 7.52 | 6.98 | 7.18 | 15.71 | 13.49 | 14.13 | 10.31 | 9.20 | 10.77 | 14.90 | 14.01 | 5.97 |
| **L. Tibia** | 57.05 | 54.15 | 5.08 | 13.13 | 10.83 | 17.52 | 13.40 | 11.10 | 17.16 | 5.18 | 4.40 | 15.06 | 12.75 | 11.10 | 12.94 |
| **R. Tibia** | 57.18 | 54.28 | 5.07 | 13.12 | 11.00 | 16.16 | 13.50 | 10.97 | 18.74 | 5.13 | 4.38 | 14.62 | 12.80 | 11.30 | 11.72 |
| **Mean** | 57.10 | 54.15 | 5.17 | 13.13 | 10.92 | 16.83 | 13.45 | 11.03 | 17.99 | 5.16 | 4.39 | 14.92 | 12.78 | 11.20 | 12.36 |
| **L. Fibula** | 56.07 | 51.10 | 8.86 | 7.23 | 6.20 | 14.25 | 7.23 | 6.20 | 14.25 | 7.70 | 5.90 | 23.38 | 6.02 | 5.05 | 16.11 |
| **R. Fibula** | — | — | — | 7.25 | 6.30 | 13.10 | 7.25 | 6.30 | 13.10 | — | — | — | — | — | — |
| **Mean** | 56.07 | 51.10 | 8.86 | 7.24 | 6.25 | 13.67 | 7.24 | 6.25 | 13.67 | 7.70 | 5.90 | 23.38 | 6.02 | 5.05 | 16.11 |
| **L. Humerus** | 61.70 | 55.85 | 9.48 | 13.52 | 13.18 | 2.51 | 13.52 | 13.18 | 2.51 | 4.50 | 4.00 | 11.11 | 12.83 | 11.01 | 14.19 |
| **R. Humerus** | 61.70 | 55.85 | 9.48 | 13.53 | 13.20 | 2.44 | 13.53 | 13.20 | 2.44 | 4.40 | 3.89 | 11.59 | 12.68 | 11.10 | 12.46 |
| **Mean** | 61.70 | 55.85 | 9.48 | 13.53 | 13.19 | 2.51 | 13.53 | 13.19 | 2.51 | 4.45 | 3.95 | 11.24 | 12.76 | 11.05 | 13.40 |
| **L. Ulna** | 49.00 | 43.90 | 10.41 | 10.50 | 8.55 | 18.57 | 11.38 | 8.55 | 24.87 | 4.45 | 2.48 | 44.27 | 7.68 | 6.85 | 10.81 |
| **R. Ulna** | 49.10 | 44.15 | 10.08 | 10.48 | 8.55 | 18.42 | — | — | — | 4.50 | 2.50 | 44.44 | 7.75 | 6.82 | 12.00 |
| **Mean** | 49.03 | 44.12 | 10.01 | 10.49 | 8.55 | 18.49 | 11.38 | 8.55 | 24.87 | 4.48 | 2.49 | 44.42 | 7.72 | 6.83 | 11.53 |
| **L. Radius** | 46.10 | 40.40 | 12.36 | 8.83 | 7.15 | 19.03 | 8.83 | 7.15 | 19.03 | 9.65 | 7.62 | 21.04 | 9.65 | 7.62 | 21.04 |
| **R. Radius** | 46.05 | 40.15 | 12.81 | 8.80 | 7.20 | 18.18 | 8.80 | 7.20 | 18.18 | 9.70 | 7.95 | 18.04 | 9.70 | 7.95 | 18.04 |
| **Mean** | 46.08 | 40.15 | 12.87 | 8.80 | 7.20 | 18.18 | 8.80 | 7.20 | 18.18 | 9.68 | 7.95 | 17.87 | 9.68 | 7.95 | 17.87 |

| ***Alligator mississippiensis*** | | | | **Proximal** | | | | | | **Distal** | | | | | |
| --- | --- | --- | --- | --- | --- | --- | --- | --- | --- | --- | --- | --- | --- | --- | --- |
| **OUVC 9408** | **GreatestLength** | **w/o cart** | **Percent change** | **Craniocaudal** | **w/o cart.** | **Percent change** | **Medio**  **lateral** | **w/o cart.** | **Percent change** | **Craniocaudal** | **w/o cart.** | **Percent change** | **Medio**  **lateral** | **w/o cart.** | **Percent change** |
| **L. Femur** | 117.13 | 110.30 | 5.83 | 18.08 | 14.20 | 21.46 | 28.02 | 24.30 | 13.28 | 18.08 | 14.20 | 21.46 | 28.48 | 26.25 | 7.83 |
| **R. Femur** | 117.20 | 110.32 | 5.87 | 18.02 | 14.25 | 20.92 | 28.07 | 24.30 | 13.43 | 18.02 | 14.25 | 20.92 | 28.47 | 26.30 | 7.62 |
| **Mean** | 117.17 | 110.31 | 5.85 | 18.05 | 14.23 | 21.16 | 28.04 | 24.30 | 13.34 | 18.05 | 14.23 | 21.16 | 28.48 | 26.28 | 7.72 |
| **L. Tibia** | 92.48 | 89.40 | 3.33 | 24.63 | 21.60 | 12.30 | 20.02 | 18.30 | 8.59 | 23.52 | 20.85 | 11.35 | 14.73 | 12.70 | 13.78 |
| **R. Tibia** | 92.78 | 89.45 | 3.59 | 24.72 | 21.60 | 12.62 | 20.07 | 18.35 | 8.57 | 23.88 | 20.85 | 12.69 | 14.55 | 12.70 | 12.71 |
| **Mean** | 92.63 | 89.43 | 3.45 | 24.68 | 21.60 | 12.48 | 20.04 | 18.33 | 8.53 | 23.70 | 20.85 | 12.03 | 14.64 | 12.70 | 13.25 |
| **L. Fibula** | 92.48 | 85.83 | 7.19 | 5.47 | 4.40 | 19.56 | 13.88 | 12.00 | 13.54 | 14.52 | 10.60 | 27.00 | 11.12 | 9.20 | 17.27 |
| **R. Fibula** | 92.18 | 85.80 | 6.92 | 5.22 | 4.40 | 15.71 | 14.32 | 12.00 | 16.20 | 15.02 | 10.60 | 29.43 | 11.32 | 9.20 | 18.73 |
| **Mean** | 92.33 | 85.82 | 7.05 | 5.34 | 4.40 | 17.60 | 14.10 | 12.00 | 14.89 | 14.77 | 10.60 | 28.23 | 11.22 | 9.20 | 18.00 |
| **L. Humerus** | 105.48 | 97.80 | 7.28 | 14.67 | 10.65 | 27.40 | 14.50 | 10.60 | 26.90 | 27.22 | 23.20 | 14.77 | 18.28 | 14.20 | 22.32 |
| **R. Humerus** | 105.32 | 97.80 | 7.14 | 14.52 | 10.60 | 27.00 | 14.59 | 10.63 | 27.14 | 27.48 | 23.20 | 15.57 | 18.47 | 14.20 | 23.12 |
| **Mean** | 105.40 | 97.80 | 7.21 | 14.59 | 10.63 | 27.14 | 14.59 | 10.63 | 27.14 | 27.35 | 23.20 | 15.17 | 18.38 | 14.20 | 22.74 |
| **L. Ulna** | 78.48 | 74.23 | 5.42 | 19.02 | 16.50 | 13.25 | 17.77 | 14.30 | 19.53 | 19.02 | 16.50 | 13.25 | 13.72 | 11.15 | 18.73 |
| **R. Ulna** | 78.42 | 74.20 | 5.38 | 20.02 | 16.50 | 17.58 | 17.72 | 14.30 | 19.30 | 20.02 | 16.50 | 17.58 | 13.98 | 11.10 | 20.60 |
| **Mean** | 78.45 | 74.22 | 5.39 | 19.52 | 16.50 | 15.47 | 17.74 | 14.30 | 19.39 | 19.52 | 16.50 | 15.47 | 13.85 | 11.13 | 19.64 |
| **L. Radius** | 71.12 | 65.60 | 7.76 | 11.13 | 9.20 | 17.34 | 11.13 | 9.20 | 17.34 | 16.48 | 13.00 | 21.12 | 9.50 | 6.30 | 33.68 |
| **R. Radius** | 71.05 | 65.60 | 7.67 | 11.12 | 9.25 | 16.82 | 11.12 | 9.25 | 16.82 | 16.48 | 13.00 | 21.12 | 9.50 | 6.30 | 33.68 |
| **Mean** | 71.08 | 65.60 | 7.71 | 11.13 | 9.23 | 17.07 | 11.13 | 9.23 | 17.07 | 16.48 | 13.00 | 21.12 | 9.50 | 6.30 | 33.68 |

| ***Alligator mississippiensis*** | | | | **Proximal** | | | | | | **Distal** | | | | | |
| --- | --- | --- | --- | --- | --- | --- | --- | --- | --- | --- | --- | --- | --- | --- | --- |
| **OUVC 9409** | **GreatestLength** | **w/o cart** | **Percent change** | **Craniocaudal** | **w/o cart.** | **Percent change** | **Medio**  **lateral** | **w/o cart.** | **Percent change** | **Craniocaudal** | **w/o cart.** | **Percent change** | **Medio**  **lateral** | **w/o cart.** | **Percent change** |
| **L. Femur** | 71.02 | 66.22 | 6.76 | 10.27 | 7.50 | 26.97 | 15.97 | 13.43 | 15.90 | 11.42 | 9.42 | 17.51 | 14.90 | 12.83 | 13.89 |
| **R. Femur** | 70.95 | 66.22 | 6.67 | 10.18 | 7.52 | 26.13 | 16.02 | 13.48 | 15.86 | 11.52 | 9.42 | 18.23 | 14.53 | 12.83 | 11.70 |
| **Mean** | 70.98 | 66.22 | 6.71 | 10.23 | 7.51 | 26.59 | 15.99 | 13.46 | 15.82 | 11.47 | 9.42 | 17.87 | 14.72 | 12.83 | 12.84 |
| **L. Tibia** | 57.98 | 55.32 | 4.59 | 12.48 | 10.62 | 14.90 | 14.02 | 11.50 | 17.97 | 8.48 | 7.15 | 15.68 | 13.20 | 11.35 | 14.02 |
| **R. Tibia** | 57.52 | 55.32 | 3.82 | 12.23 | 10.60 | 13.33 | 14.12 | 11.52 | 18.41 | 8.42 | 7.15 | 15.08 | 13.02 | 11.35 | 12.83 |
| **Mean** | 57.75 | 55.32 | 4.21 | 12.36 | 10.61 | 14.16 | 14.07 | 11.51 | 18.19 | 8.45 | 7.15 | 15.38 | 13.11 | 11.35 | 13.42 |
| **L. Fibula** | 56.62 | 52.40 | 7.45 | 7.42 | 6.30 | 15.09 | 4.52 | 2.80 | 38.05 | 6.00 | 4.65 | 22.50 | 8.48 | 6.25 | 26.30 |
| **R. Fibula** | 56.48 | 52.40 | 7.22 | 7.42 | 6.30 | 15.09 | 4.50 | 2.80 | 37.78 | 6.07 | 4.65 | 23.39 | 8.50 | 6.25 | 26.47 |
| **Mean** | 56.55 | 52.40 | 7.34 | 7.42 | 6.30 | 15.09 | 4.51 | 2.80 | 37.92 | 6.03 | 4.65 | 22.89 | 8.49 | 6.25 | 26.38 |
| **L. Humerus** | 64.45 | 59.05 | 8.38 | 7.95 | 5.55 | 30.19 | 13.92 | 11.15 | 19.90 | 10.35 | 8.05 | 22.22 | 16.03 | 13.25 | 17.34 |
| **R. Humerus** | 64.48 | 59.05 | 8.42 | 7.92 | 5.55 | 29.92 | 13.95 | 11.18 | 19.86 | 10.38 | 8.02 | 22.74 | 16.02 | 13.25 | 17.29 |
| **Mean** | 64.47 | 59.05 | 8.41 | 7.93 | 5.55 | 30.01 | 13.93 | 11.17 | 19.81 | 10.37 | 8.03 | 22.57 | 16.03 | 13.25 | 17.34 |
| **L. Ulna** | 51.95 | 47.88 | 7.83 | 10.72 | 7.90 | 26.31 | 11.42 | 9.20 | 19.44 | 5.40 | 4.10 | 24.07 | 7.62 | 6.32 | 17.06 |
| **R. Ulna** | 51.98 | 47.87 | 7.91 | 10.32 | 7.90 | 23.45 | 11.58 | 9.22 | 20.38 | 5.82 | 4.12 | 29.21 | 7.48 | 6.30 | 15.78 |
| **Mean** | 51.97 | 47.88 | 7.87 | 10.52 | 7.90 | 24.90 | 11.50 | 9.21 | 19.91 | 5.63 | 4.11 | 27.00 | 7.55 | 6.31 | 16.42 |
| **L. Radius** | 46.92 | 42.45 | 9.53 | 6.47 | 5.10 | 21.17 | 8.62 | 7.15 | 17.05 | 5.48 | 4.10 | 25.18 | 9.02 | 7.37 | 18.29 |
| **R. Radius** | 46.98 | 43.45 | 7.51 | 6.48 | 5.10 | 21.30 | 8.68 | 7.15 | 17.63 | 5.53 | 4.13 | 25.32 | 9.08 | 7.40 | 18.50 |
| **Mean** | 46.95 | 42.95 | 8.52 | 6.48 | 5.10 | 21.30 | 8.65 | 7.15 | 17.34 | 5.51 | 4.12 | 25.23 | 9.05 | 7.38 | 18.45 |

| ***Alligator mississippiensis*** | | | | **Proximal** | | | | | | **Distal** | | | | | |
| --- | --- | --- | --- | --- | --- | --- | --- | --- | --- | --- | --- | --- | --- | --- | --- |
| **OUVC 9410** | **GreatestLength** | **w/o cart** | **Percent change** | **Craniocaudal** | **w/o cart.** | **Percent change** | **Medio**  **lateral** | **w/o cart.** | **Percent change** | **Craniocaudal** | **w/o cart.** | **Percent change** | **Medio**  **lateral** | **w/o cart.** | **Percent change** |
| **L. Femur** | 69.27 | 64.30 | 7.17 | 10.15 | 7.00 | 31.03 | 15.32 | 13.30 | 13.19 | 10.53 | 8.65 | 17.85 | 15.52 | 13.30 | 14.30 |
| **R. Femur** | 69.17 | 64.32 | 7.01 | 10.22 | 7.00 | 31.51 | 15.62 | 13.30 | 14.85 | 10.42 | 8.60 | 17.47 | 15.47 | 13.30 | 14.03 |
| **Mean** | 69.22 | 64.31 | 7.09 | 10.18 | 7.00 | 31.24 | 15.47 | 13.30 | 14.03 | 10.48 | 8.63 | 17.65 | 15.49 | 13.30 | 14.14 |
| **L. Tibia** | 56.30 | 53.40 | 5.15 | 13.82 | 10.70 | 22.58 | 13.82 | 10.70 | 22.58 | 13.00 | 11.62 | 10.62 | 7.72 | 6.00 | 22.28 |
| **R. Tibia** | 56.37 | 53.42 | 5.23 | 13.73 | 10.70 | 22.07 | 13.73 | 10.70 | 22.07 | 13.12 | 11.60 | 11.59 | 7.67 | 6.00 | 21.77 |
| **Mean** | 56.33 | 53.41 | 5.18 | 13.78 | 10.70 | 22.35 | 13.78 | 10.70 | 22.35 | 13.06 | 11.61 | 11.10 | 7.69 | 6.00 | 21.98 |
| **L. Fibula** | 55.03 | 51.10 | 7.14 | 3.83 | 2.30 | 39.95 | 3.83 | 2.30 | 39.95 | 8.67 | 6.10 | 29.64 | 5.90 | 4.30 | 27.12 |
| **R. Fibula** | 55.00 | 51.13 | 7.04 | 3.82 | 2.35 | 38.48 | 3.82 | 2.35 | 38.48 | 8.70 | 6.10 | 29.89 | 5.93 | 4.30 | 27.49 |
| **Mean** | 55.02 | 51.12 | 7.09 | 3.83 | 2.33 | 39.16 | 3.83 | 2.33 | 39.16 | 8.68 | 6.10 | 29.72 | 5.92 | 4.30 | 27.36 |
| **L. Humerus** | 62.93 | 56.90 | 9.58 | 7.57 | 5.45 | 28.01 | 7.57 | 5.45 | 28.01 | 10.02 | 7.10 | 29.14 | 15.82 | 13.00 | 17.83 |
| **R. Humerus** | 62.98 | 56.90 | 9.65 | 7.63 | 5.40 | 29.23 | 7.63 | 5.40 | 29.23 | 10.08 | 7.10 | 29.56 | 15.90 | 13.00 | 18.24 |
| **Mean** | 62.96 | 56.90 | 9.63 | 7.60 | 5.43 | 28.55 | 7.60 | 5.43 | 28.55 | 10.05 | 7.10 | 29.35 | 15.86 | 13.00 | 18.03 |
| **L. Ulna** | 48.58 | 44.60 | 8.19 | 10.13 | 7.30 | 27.94 | 11.28 | 8.70 | 22.87 | 4.28 | 3.40 | 20.56 | 7.12 | 5.85 | 17.84 |
| **R. Ulna** | 48.43 | 44.60 | 7.91 | 9.63 | 7.00 | 27.31 | 11.53 | 8.70 | 24.54 | 4.92 | 3.40 | 30.89 | 7.00 | 5.85 | 16.43 |
| **Mean** | 48.51 | 44.60 | 8.06 | 9.88 | 7.15 | 27.63 | 11.41 | 8.70 | 23.75 | 4.60 | 3.40 | 26.09 | 7.06 | 5.85 | 17.14 |
| **L. Radius** | 44.33 | 40.20 | 9.32 | 8.18 | 6.60 | 19.32 | 8.18 | 6.60 | 19.32 | 8.12 | 7.50 | 7.64 | 5.52 | 3.70 | 32.97 |
| **R. Radius** | 44.35 | 40.20 | 9.36 | 8.22 | 6.60 | 19.71 | 8.22 | 6.60 | 19.71 | 8.15 | 7.50 | 7.98 | 5.62 | 3.70 | 34.16 |
| **Mean** | 44.34 | 40.20 | 9.34 | 8.20 | 6.60 | 19.51 | 8.20 | 6.60 | 19.51 | 8.13 | 7.50 | 7.75 | 5.57 | 3.70 | 33.57 |

| ***Alligator mississippiensis*** | | | | **Proximal** | | | | | | **Distal** | | | | | |
| --- | --- | --- | --- | --- | --- | --- | --- | --- | --- | --- | --- | --- | --- | --- | --- |
| **OUVC 9411** | **GreatestLength** | **w/o cart** | **Percent change** | **Craniocaudal** | **w/o cart.** | **Percent change** | **Medio**  **lateral** | **w/o cart.** | **Percent change** | **Craniocaudal** | **w/o cart.** | **Percent change** | **Medio**  **lateral** | **w/o cart.** | **Percent change** |
| **L. Femur** | 73.98 | 67.90 | 8.22 | 10.40 | 6.90 | 33.65 | 15.75 | 12.50 | 20.63 | 11.45 | 8.70 | 24.02 | 16.17 | 14.00 | 14.30 |
| **R. Femur** | 73.83 | 67.95 | 7.96 | 10.35 | 6.90 | 33.33 | 15.75 | 12.50 | 20.63 | 11.53 | 8.70 | 24.54 | 16.20 | 14.00 | 14.03 |
| **Mean** | 73.91 | 67.93 | 8.09 | 10.38 | 6.90 | 33.53 | 15.75 | 12.50 | 20.63 | 11.49 | 8.70 | 24.28 | 16.18 | 14.00 | 14.14 |
| **L. Tibia** | 58.28 | 55.55 | 4.68 | 13.80 | 10.90 | 21.01 | 11.93 | 9.60 | 19.53 | 12.43 | 10.75 | 13.52 | 8.00 | 6.40 | 22.28 |
| **R. Tibia** | 58.47 | 55.55 | 4.99 | 12.77 | 10.90 | 14.64 | 11.92 | 9.60 | 19.46 | 12.50 | 10.75 | 14.00 | 8.00 | 6.40 | 21.77 |
| **Mean** | 58.38 | 55.55 | 4.85 | 13.28 | 10.90 | 17.92 | 11.95 | 9.60 | 19.67 | 12.47 | 10.75 | 13.79 | 8.00 | 6.40 | 21.98 |
| **L. Fibula** | 58.28 | 53.75 | 7.77 | 8.07 | 6.30 | 21.93 | 4.23 | 2.50 | 40.90 | 6.13 | 4.50 | 26.59 | 7.82 | 6.00 | 27.12 |
| **R. Fibula** | 58.22 | 54.40 | 6.56 | 8.00 | 6.30 | 21.25 | 4.20 | 2.50 | 40.48 | 6.00 | 4.50 | 25.00 | 7.72 | 6.00 | 27.49 |
| **Mean** | 58.25 | 54.08 | 7.16 | 8.03 | 6.30 | 21.54 | 4.22 | 2.50 | 40.76 | 6.07 | 4.50 | 25.86 | 7.77 | 6.00 | 27.36 |
| **L. Humerus** | 68.58 | 62.00 | 9.59 | 8.12 | 5.35 | 34.11 | 15.25 | 13.80 | 9.51 | 9.82 | 7.00 | 28.72 | 15.02 | 12.70 | 17.83 |
| **R. Humerus** | 68.82 | 62.00 | 9.91 | 8.17 | 5.30 | 35.13 | 15.28 | 13.80 | 9.69 | 9.90 | 7.00 | 29.29 | 15.03 | 12.70 | 18.24 |
| **Mean** | 68.70 | 62.00 | 9.75 | 8.14 | 5.33 | 34.52 | 15.27 | 13.80 | 9.63 | 9.86 | 7.00 | 29.01 | 15.03 | 12.70 | 18.03 |
| **L. Ulna** | 50.58 | 47.00 | 7.08 | 10.37 | 7.70 | 25.75 | 11.63 | 8.30 | 28.63 | 7.67 | 6.20 | 19.17 | 4.67 | 3.85 | 17.84 |
| **R. Ulna** | 50.60 | 47.00 | 7.11 | 10.42 | 7.70 | 26.10 | 11.68 | 8.30 | 28.94 | 7.70 | 6.20 | 19.48 | 4.70 | 3.85 | 16.43 |
| **Mean** | 50.59 | 47.00 | 7.10 | 10.39 | 7.70 | 25.89 | 11.66 | 8.30 | 28.82 | 7.68 | 6.20 | 19.27 | 4.68 | 3.85 | 17.14 |
| **L. Radius** | 46.53 | 41.70 | 10.38 | 8.67 | 6.83 | 21.22 | 6.55 | 5.30 | 19.08 | 10.05 | 7.20 | 28.36 | 6.83 | 5.67 | 32.97 |
| **R. Radius** | 46.57 | 41.70 | 10.46 | 8.70 | 6.83 | 21.49 | 6.60 | 5.30 | 19.70 | 10.03 | 7.20 | 28.22 | 6.83 | 5.70 | 34.16 |
| **Mean** | 46.55 | 41.70 | 10.42 | 8.68 | 6.83 | 21.31 | 6.58 | 5.30 | 19.45 | 10.04 | 7.20 | 28.29 | 6.83 | 5.68 | 33.57 |

| ***Alligator mississippiensis*** | | | | **Proximal** | | | | | | **Distal** | | | | | |
| --- | --- | --- | --- | --- | --- | --- | --- | --- | --- | --- | --- | --- | --- | --- | --- |
| **OUVC 9412** | **GreatestLength** | **w/o cart** | **Percent change** | **Craniocaudal** | **w/o cart.** | **Percent change** | **Medio**  **lateral** | **w/o cart.** | **Percent change** | **Craniocaudal** | **w/o cart.** | **Percent change** | **Medio**  **lateral** | **w/o cart.** | **Percent change** |
| **L. Femur** | 169.00 | 160.00 | 5.33 | 25.60 | 21.12 | 17.50 | 42.45 | 39.30 | 7.42 | 30.62 | 27.30 | 10.84 | 30.62 | 27.30 | 10.84 |
| **R. Femur** | 167.00 | 159.00 | 4.79 | 25.60 | 21.72 | 15.16 | 43.80 | 38.80 | 11.42 | 30.50 | 27.80 | 8.85 | 30.50 | 27.80 | 8.85 |
| **Mean** | 168.00 | 159.50 | 5.06 | 25.60 | 21.42 | 16.33 | 43.13 | 39.05 | 9.46 | 30.56 | 27.55 | 9.85 | 30.56 | 27.55 | 9.85 |
| **L. Tibia** | 131.42 | 129.00 | 1.84 | 36.25 | 33.40 | 7.86 | 29.95 | 27.30 | 8.85 | 21.40 | 19.12 | 10.65 | 21.40 | 19.12 | 10.65 |
| **R. Tibia** | 131.53 | 127.92 | 2.74 | 35.80 | 33.40 | 6.70 | 30.08 | 27.40 | 8.91 | 21.40 | 18.62 | 12.99 | 21.40 | 18.62 | 12.99 |
| **Mean** | 131.48 | 128.46 | 2.30 | 36.03 | 33.40 | 7.30 | 30.02 | 27.35 | 8.89 | 21.40 | 18.87 | 11.82 | 21.40 | 18.87 | 11.82 |
| **L. Fibula** | 131.00 | 123.45 | 5.76 | 21.15 | 18.00 | 14.89 | 8.80 | 7.50 | 14.77 | 15.50 | 13.10 | 15.48 | 15.50 | 13.10 | 15.48 |
| **R. Fibula** | 129.23 | 123.00 | 4.82 | 21.05 | 18.50 | 12.11 | 9.20 | 7.30 | 20.65 | 15.95 | 13.60 | 14.73 | 15.95 | 13.60 | 14.73 |
| **Mean** | 130.12 | 123.23 | 5.30 | 21.10 | 18.25 | 13.51 | 9.00 | 7.40 | 17.78 | 15.73 | 13.35 | 15.13 | 15.73 | 13.35 | 15.13 |
| **L. Humerus** | 158.00 | 150.00 | 5.06 | 14.60 | 10.50 | 28.08 | 25.00 | 21.90 | 12.40 | 18.20 | 14.15 | 22.25 | 18.20 | 14.15 | 22.25 |
| **R. Humerus** | 158.00 | 150.00 | 5.06 | 14.93 | 10.50 | 29.67 | 25.00 | 21.90 | 12.40 | 24.90 | 23.20 | 6.83 | 24.90 | 23.20 | 6.83 |
| **Mean** | 158.00 | 150.00 | 5.06 | 14.77 | 10.50 | 28.91 | 25.00 | 21.90 | 12.40 | 25.20 | 23.95 | 4.96 | 25.20 | 23.95 | 4.96 |
| **L. Ulna** | 120.62 | 112.02 | 7.13 | 28.80 | 23.70 | 17.71 | 31.40 | 26.50 | 15.61 | 12.10 | 11.00 | 9.09 | 12.10 | 11.00 | 9.09 |
| **R. Ulna** | 120.00 | 112.00 | 6.67 | 28.40 | 24.70 | 13.03 | 30.20 | 27.20 | 9.93 | 11.50 | 10.23 | 11.04 | 11.50 | 10.23 | 11.04 |
| **Mean** | 120.31 | 112.01 | 6.90 | 28.60 | 24.20 | 15.38 | 30.80 | 26.85 | 12.82 | 11.80 | 10.62 | 10.00 | 11.80 | 10.62 | 10.00 |
| **L. Radius** | 78.00 | 74.20 | 4.87 | 14.50 | 12.80 | 11.72 | 11.00 | 9.60 | 12.73 | 15.80 | 13.92 | 11.90 | 15.80 | 13.92 | 11.90 |
| **R. Radius** | 78.70 | 74.20 | 5.72 | 15.00 | 13.00 | 13.33 | 11.40 | 9.70 | 14.91 | 16.00 | 14.00 | 12.50 | 16.00 | 14.00 | 12.50 |
| **Mean** | 78.35 | 74.20 | 5.30 | 14.75 | 12.90 | 12.54 | 11.20 | 9.65 | 13.84 | 15.90 | 13.96 | 12.20 | 15.90 | 13.96 | 12.20 |

| ***Alligator mississippiensis*** | | | | **Proximal** | | | | | | **Distal** | | | | | |
| --- | --- | --- | --- | --- | --- | --- | --- | --- | --- | --- | --- | --- | --- | --- | --- |
| **OUVC 9413** | **GreatestLength** | **w/o cart** | **Percent change** | **Craniocaudal** | **w/o cart.** | **Percent change** | **Lateromedial** | **w/o cart.** | **Percent change** | **Craniocaudal** | **w/o cart.** | **Percent change** | **Lateromedial** | **w/o cart.** | **Percent change** |
| **L. Femur** | 174.00 | 167.00 | 4.02 | 24.00 | 20.60 | 14.17 | 39.55 | 37.20 | 5.94 | 29.28 | 26.40 | 9.84 | 40.02 | 38.50 | 3.80 |
| **R. Femur** | 174.00 | 167.00 | 4.02 | 24.00 | 20.40 | 15.00 | 40.50 | 37.20 | 8.15 | 28.70 | 26.50 | 7.67 | 40.30 | 38.70 | 3.97 |
| **Mean** | 174.00 | 167.00 | 4.02 | 24.00 | 20.50 | 14.58 | 40.03 | 37.20 | 7.07 | 28.99 | 26.45 | 8.76 | 40.16 | 38.60 | 3.88 |
| **L. Tibia** | 131.00 | 129.20 | 1.37 | 35.42 | 31.80 | 10.22 | 31.42 | 27.50 | 12.48 | 31.82 | 30.30 | 4.78 | 19.20 | 19.00 | 1.04 |
| **R. Tibia** | 130.00 | 128.93 | 0.82 | 35.80 | 33.10 | 7.54 | 31.15 | 27.50 | 11.72 | 32.40 | 30.50 | 5.86 | 20.52 | 19.00 | 7.41 |
| **Mean** | 130.50 | 129.07 | 1.10 | 35.61 | 32.45 | 8.87 | 31.28 | 27.50 | 12.08 | 32.11 | 30.40 | 5.33 | 19.86 | 19.00 | 4.33 |
| **L. Fibula** | 133.00 | 126.30 | 5.04 | 19.42 | 17.50 | 9.89 | 8.00 | 6.70 | 16.25 | 15.53 | 13.30 | 14.36 | 20.62 | 18.50 | 10.28 |
| **R. Fibula** | 131.62 | 126.50 | 3.89 | 19.00 | 17.25 | 9.21 | 8.37 | 7.00 | 16.37 | 15.52 | 13.30 | 14.30 | 21.20 | 18.52 | 12.64 |
| **Mean** | 132.31 | 126.40 | 4.47 | 19.21 | 17.38 | 9.53 | 8.18 | 6.85 | 16.26 | 15.53 | 13.30 | 14.36 | 20.91 | 18.51 | 11.48 |
| **L. Humerus** | 158.00 | 151.00 | 4.43 | 22.00 | 18.25 | 17.05 | 34.90 | 32.00 | 8.31 | 26.80 | 20.90 | 22.01 | 36.90 | 35.70 | 3.25 |
| **R. Humerus** | 158.00 | 151.00 | 4.43 | 22.00 | 18.25 | 17.05 | 34.90 | 32.00 | 8.31 | 26.82 | 20.90 | 22.07 | 36.90 | 35.70 | 3.25 |
| **Mean** | 158.00 | 151.00 | 4.43 | 22.20 | 18.53 | 16.53 | 35.49 | 31.85 | 10.26 | 26.99 | 21.25 | 21.27 | 37.30 | 35.75 | 4.16 |
| **L. Ulna** | 124.68 | 116.27 | 6.75 | 28.43 | 24.40 | 14.18 | 33.40 | 28.70 | 14.07 | 11.53 | 10.25 | 11.10 | 20.60 | 18.00 | 12.62 |
| **R. Ulna** | 124.32 | 115.50 | 7.09 | 27.50 | 25.00 | 9.09 | 32.20 | 28.20 | 12.42 | 11.33 | 10.70 | 5.56 | 20.92 | 18.20 | 13.00 |
| **Mean** | 124.50 | 115.88 | 6.92 | 27.97 | 24.70 | 11.69 | 32.80 | 28.45 | 13.26 | 11.43 | 10.48 | 8.31 | 20.76 | 18.10 | 12.81 |
| **L. Radius** | 106.00 | 100.20 | 5.47 | 23.20 | 21.00 | 9.48 | 15.82 | 13.60 | 14.03 | 13.80 | 11.30 | 18.12 | 23.60 | 20.40 | 13.56 |
| **R. Radius** | 106.00 | 100.50 | 5.19 | 23.20 | 20.65 | 10.99 | 15.72 | 13.73 | 12.66 | 14.40 | 11.50 | 20.14 | 23.05 | 20.70 | 10.20 |
| **Mean** | 106.00 | 100.35 | 5.33 | 23.20 | 20.83 | 10.22 | 15.77 | 13.67 | 13.32 | 14.10 | 11.40 | 19.15 | 23.33 | 20.55 | 11.92 |

| ***Alligator mississippiensis*** | | | | **Proximal** | | | | | | **Distal** | | | | | |
| --- | --- | --- | --- | --- | --- | --- | --- | --- | --- | --- | --- | --- | --- | --- | --- |
| **OUVC 9414** | **GreatestLength** | **w/o cart** | **Percent change** | **Craniocaudal** | **w/o cart.** | **Percent change** | **Lateromedial** | **w/o cart.** | **Percent change** | **Craniocaudal** | **w/o cart.** | **Percent change** | **Lateromedial** | **w/o cart.** | **Percent change** |
| **L. Femur** | 157.00 | 151.00 | 3.82 | 22.50 | 18.80 | 16.44 | 37.25 | 34.25 | 8.05 | 24.10 | 23.10 | 4.15 | 35.30 | 34.10 | 3.40 |
| **R. Femur** | 157.00 | 150.80 | 3.95 | 22.00 | 18.90 | 14.09 | 37.15 | 34.00 | 8.48 | 24.00 | 22.90 | 4.58 | 35.70 | 34.20 | 4.20 |
| **Mean** | 157.00 | 150.90 | 3.89 | 22.25 | 18.85 | 15.28 | 37.20 | 34.13 | 8.25 | 24.05 | 23.00 | 4.37 | 35.50 | 34.15 | 3.80 |
| **L. Tibia** | 120.02 | 118.00 | 1.68 | 33.20 | 30.10 | 9.34 | 22.23 | 21.00 | 5.53 | 31.18 | 28.60 | 8.27 | 19.12 | 16.70 | 12.66 |
| **R. Tibia** | 121.70 | 118.50 | 2.63 | 33.40 | 31.05 | 7.04 | 22.90 | 21.00 | 8.30 | 30.48 | 28.25 | 7.32 | 19.00 | 16.90 | 11.05 |
| **Mean** | 120.86 | 118.25 | 2.16 | 33.30 | 30.58 | 8.17 | 22.57 | 21.00 | 6.96 | 30.83 | 28.43 | 7.78 | 19.06 | 16.80 | 11.86 |
| **L. Fibula** | 122.23 | 115.80 | 5.26 | 18.60 | 16.95 | 8.87 | 7.50 | 6.10 | 18.67 | 13.81 | 12.45 | 9.85 | 18.52 | 15.50 | 16.31 |
| **R. Fibula** | 121.43 | 116.50 | 4.06 | 18.65 | 16.30 | 12.60 | 7.50 | 6.05 | 19.33 | 13.80 | 12.40 | 10.14 | 18.72 | 15.25 | 18.54 |
| **Mean** | 121.83 | 116.15 | 4.66 | 18.63 | 16.63 | 10.74 | 7.50 | 6.08 | 18.93 | 13.82 | 12.50 | 9.55 | 18.62 | 15.38 | 17.40 |
| **L. Humerus** | 143.10 | 136.00 | 4.96 | 19.15 | 14.50 | 24.28 | 32.30 | 27.10 | 16.10 | 23.20 | 19.20 | 17.24 | 33.50 | 32.00 | 4.48 |
| **R. Humerus** | 143.02 | 135.60 | 5.19 | 19.15 | 14.50 | 24.28 | 32.42 | 27.60 | 14.87 | 23.22 | 19.20 | 17.31 | 33.50 | 32.00 | 4.48 |
| **Mean** | 143.06 | 135.80 | 5.07 | 19.10 | 14.55 | 23.82 | 32.36 | 27.35 | 15.48 | 23.36 | 19.00 | 18.66 | 33.28 | 32.15 | 3.40 |
| **L. Ulna** | 111.23 | 103.20 | 7.22 | 24.00 | 19.60 | 18.33 | 28.82 | 23.90 | 17.07 | 10.00 | 9.50 | 5.00 | 18.65 | 14.70 | 21.18 |
| **R. Ulna** | 111.38 | 103.00 | 7.52 | 23.15 | 19.25 | 16.85 | 27.50 | 24.20 | 12.00 | 10.03 | 8.80 | 12.26 | 18.37 | 15.50 | 15.62 |
| **Mean** | 111.38 | 103.00 | 7.52 | 23.58 | 19.43 | 17.60 | 28.16 | 24.05 | 14.60 | 10.02 | 9.15 | 8.68 | 18.51 | 15.10 | 18.42 |
| **L. Radius** | 97.22 | 91.30 | 6.09 | 20.00 | 18.10 | 9.50 | 13.10 | 12.40 | 5.34 | 23.00 | 19.50 | 15.22 | 11.40 | 9.05 | 20.61 |
| **R. Radius** | 96.80 | 90.90 | 6.10 | 20.80 | 18.60 | 10.58 | 13.70 | 12.30 | 10.22 | 22.10 | 19.30 | 12.67 | 12.30 | 9.20 | 25.20 |
| **Mean** | 97.01 | 91.10 | 6.09 | 20.40 | 18.35 | 10.05 | 13.40 | 12.35 | 7.84 | 22.55 | 19.40 | 13.97 | 11.85 | 9.13 | 22.95 |

| ***Alligator mississippiensis*** | | | | **Proximal** | | | | | | **Distal** | | | | | |
| --- | --- | --- | --- | --- | --- | --- | --- | --- | --- | --- | --- | --- | --- | --- | --- |
| **OUVC 9415** | **GreatestLength** | **w/o cart** | **Percent change** | **Craniocaudal** | **w/o cart.** | **Percent change** | **Lateromedial** | **w/o cart.** | **Percent change** | **Craniocaudal** | **w/o cart.** | **Percent change** | **Lateromedial** | **w/o cart.** | **Percent change** |
| **L. Femur** | 127.60 | 121.00 | 5.17 | 18.45 | 14.50 | 21.41 | 29.00 | 25.50 | 12.07 | 20.15 | 17.00 | 15.63 | 28.50 | 26.50 | 15.63 |
| **R. Femur** | 128.40 | 121.50 | 5.37 | 18.70 | 14.50 | 22.46 | 28.00 | 25.25 | 9.82 | 19.95 | 17.00 | 14.79 | 28.90 | 26.50 | 14.79 |
| **Mean** | 128.00 | 121.25 | 5.27 | 18.58 | 14.50 | 21.96 | 28.50 | 25.38 | 10.95 | 20.05 | 17.00 | 15.21 | 28.70 | 26.50 | 15.21 |
| **L. Tibia** | 100.92 | 98.50 | 2.40 | 26.00 | 23.00 | 11.54 | 20.65 | 18.60 | 9.93 | 23.73 | 21.50 | 9.40 | 13.65 | 12.10 | 9.40 |
| **R. Tibia** | 101.00 | 98.70 | 2.28 | 25.20 | 22.75 | 9.72 | 20.30 | 18.60 | 8.37 | 23.20 | 21.60 | 6.90 | 14.22 | 12.50 | 6.90 |
| **Mean** | 100.96 | 98.60 | 2.34 | 25.60 | 22.88 | 10.63 | 20.48 | 18.60 | 9.18 | 23.47 | 21.55 | 8.18 | 13.93 | 12.30 | 8.18 |
| **L. Fibula** | 100.20 | 95.00 | 5.19 | 13.50 | 12.40 | 8.15 | 4.98 | 4.25 | 14.66 | 10.75 | 9.30 | 13.49 | 13.80 | 12.00 | 13.49 |
| **R. Fibula** | 100.22 | 95.00 | 5.21 | 13.75 | 12.40 | 9.82 | 5.35 | 4.50 | 15.89 | 11.40 | 9.40 | 17.54 | 14.35 | 12.30 | 17.54 |
| **Mean** | 100.21 | 95.00 | 5.20 | 13.63 | 12.40 | 9.02 | 4.98 | 4.25 | 14.66 | 11.08 | 9.35 | 15.61 | 14.08 | 12.15 | 15.61 |
| **L. Humerus** | 116.03 | 109.70 | 5.46 | 14.60 | 10.50 | 28.08 | 25.00 | 21.90 | 12.40 | 18.20 | 14.15 | 22.25 | 25.50 | 24.70 | 22.25 |
| **R. Humerus** | 116.32 | 109.80 | 5.61 | 14.93 | 10.50 | 29.67 | 25.00 | 21.90 | 12.40 | 18.10 | 14.10 | 22.10 | 24.90 | 23.20 | 22.10 |
| **Mean** | 116.18 | 109.75 | 5.53 | 14.77 | 10.50 | 28.91 | 25.00 | 21.90 | 12.40 | 18.15 | 14.13 | 22.15 | 25.20 | 23.95 | 22.15 |
| **L. Ulna** | 89.53 | 82.90 | 7.41 | 17.23 | 15.00 | 12.94 | 19.43 | 17.40 | 10.45 | 8.23 | 7.00 | 14.95 | 15.15 | 10.80 | 14.95 |
| **R. Ulna** | 89.00 | 82.70 | 7.08 | 18.00 | 15.47 | 14.06 | 20.70 | 17.50 | 15.46 | 8.33 | 7.00 | 15.97 | 14.40 | 10.50 | 15.97 |
| **Mean** | 89.27 | 82.80 | 7.25 | 17.62 | 15.23 | 13.56 | 20.07 | 17.45 | 13.05 | 8.28 | 7.00 | 15.46 | 14.78 | 10.65 | 15.46 |
| **L. Radius** | 78.00 | 74.20 | 4.87 | 14.50 | 12.80 | 11.72 | 11.00 | 9.60 | 12.73 | 15.80 | 13.92 | 11.90 | 10.30 | 7.10 | 11.90 |
| **R. Radius** | 78.70 | 74.20 | 5.72 | 15.00 | 13.00 | 13.33 | 11.40 | 9.70 | 14.91 | 16.00 | 14.00 | 12.50 | 9.00 | 7.00 | 12.50 |
| **Mean** | 78.35 | 74.20 | 5.30 | 14.75 | 12.90 | 12.54 | 11.20 | 9.65 | 13.84 | 15.90 | 13.96 | 12.20 | 9.65 | 7.05 | 12.20 |

| ***Struthio camelus*** | | | | | | | | | | | | | | | | | |
| --- | --- | --- | --- | --- | --- | --- | --- | --- | --- | --- | --- | --- | --- | --- | --- | --- | --- |
| **OUVC 9422** | **Length** | | | | | **Proximal** | | | | | | **Distal** | | | | | |
| **Element** | **Condyle-Trochanter** | **w/o cart** | **Trochanter-Head** | **w/o**  **Cart.** | **Percent Change** | **Medio**  **lateral** | **w/o Cart** | **Percent Change** | **Cranio**  **caudal** | **w/o Cart** | **Percent Change** | **Medio**  **lateral** | **w/o Cart** | **Percent Change** | **Cranio**  **caudal** | **w/o Cart** | **Percent Change** |
| **R. Femur** | 305.00 | 285.00 | 109.00 | 98.50 |  | 50.97 | 33.63 |  | 50.43 | 41.38 |  | 85.53 | 78.80 |  | 88.63 | 82.15 |  |
| **L. Femur** | 303.00 | 280.00 | 108.00 | 88.00 |  | 50.40 | 36.40 |  | 50.10 | 42.80 |  | 85.50 | 79.50 |  | 89.20 | 83.00 |  |
| **Mean** | 304.00 | 282.50 | 108.50 | 93.25 | 7.53 | 50.68 | 35.02 | 5.89 | 50.27 | 42.09 | 1.46 | 85.52 | 79.15 | 1.10 | 88.92 | 82.58 | 5.44 |
| **R.Tibia** | 518.67 | 490.00 |  |  |  | 82.00 | 76.25 |  | 126.63 | 108.25 |  | 69.90 | 60.57 |  | 60.57 | 55.30 |  |
| **L. Tibia** | 519.00 | 490.00 |  |  |  | 82.10 | 78.00 |  | 129.00 | 111.80 |  | 68.80 | 63.30 |  | 59.80 | 57.60 |  |
| **Mean** | 518.83 | 490.00 |  |  | 2.99 | 82.05 | 77.13 | 0.07 | 127.82 | 110.03 | 2.55 | 69.35 | 61.93 | 7.68 | 60.18 | 56.45 | 2.64 |
| **R. Fibula** | 521.00 | 497.00 |  |  |  | 24.20 | 20.80 |  | 43.37 | 39.40 |  |  |  |  |  |  |  |
| **L. Fibula** | 520.00 | 499.00 |  |  |  | 24.00 | 19.80 |  | 43.40 | 40.90 |  |  |  |  |  |  |  |
| **Mean** | 520.50 | 498.00 |  |  | 4.32 | 24.10 | 20.30 | 15.77 | 43.38 | 40.15 | 7.45 |  |  |  |  |  |  |
| **L. Humerus** | 366.67 | 354.00 |  |  |  | 33.17 | 31.00 |  | 19.12 | 16.35 |  | 30.00 | 27.50 |  | 23.27 | 16.50 |  |
| **R. Humerus** | 365.00 | 350.00 |  |  |  | 34.43 | 31.00 |  | 19.40 | 16.45 |  | 28.52 | 27.50 |  | 23.33 | 16.50 |  |
| **Mean** | 365.83 | 352.00 |  |  | 3.78 | 33.80 | 31.00 | 8.22 | 19.26 | 16.40 | 14.84 | 29.26 | 27.50 | 6.01 | 23.30 | 16.50 | 29.18 |
| **L. Ulna** | 124.50 | 114.17 |  |  |  | 22.42 | 18.43 |  | 23.67 | 20.45 |  | 25.97 | 22.78 |  | 17.87 | 13.00 |  |
| **R. Ulna** | 124.63 | 114.10 |  |  |  | 23.10 | 18.50 |  | 24.13 | 20.50 |  | 26.53 | 22.80 |  | 17.78 | 13.00 |  |
| **Mean** | 124.57 | 114.13 |  |  | 8.38 | 22.76 | 18.47 | 18.85 | 23.90 | 20.48 | 11.56 | 26.25 | 22.79 | 13.18 | 17.83 | 13.00 | 27.09 |
| **L. Radius** | 110.00 | 107.92 |  |  |  | 12.55 | 9.78 |  | 13.23 | 11.70 |  | 18.92 | 17.42 |  | 12.03 | 10.30 |  |
| **R. Radius** | 113.63 | 108.00 |  |  |  | 13.22 | 9.90 |  | 13.38 | 11.70 |  | 19.10 | 17.35 |  | 12.00 | 10.30 |  |
| **Mean** | 111.82 | 107.96 |  |  | 3.45 | 12.88 | 9.84 | 23.60 | 13.31 | 11.70 | 12.10 | 19.01 | 17.38 | 8.57 | 12.02 | 10.30 | 14.31 |

| ***Struthio camelus*** | | | | | | | | | | | | | | | | | |
| --- | --- | --- | --- | --- | --- | --- | --- | --- | --- | --- | --- | --- | --- | --- | --- | --- | --- |
| **OUVC 9423** | **Length** | | | | | **Proximal** | | | | | | **Distal** | | | | | |
| **Element** | **Condyle-Trochanter** | **w/o cart** | **Trochanter-Head** | **w/o**  **Cart.** | **Percent Change** | **Medio**  **lateral** | **w/o Cart** | **Percent Change** | **Cranio**  **caudal** | **w/o Cart** | **Percent Change** | **Medio**  **lateral** | **w/o Cart** | **Percent Change** | **Cranio**  **caudal** | **w/o Cart** | **Percent Change** |
| **R. Femur** | 285.00 | 260.00 | 80.00 | 75.00 |  | 47.30 | 45.00 |  | 45.00 | 44.00 |  | 77.65 | 76.65 |  | 97.35 | 90.00 |  |
| **L. Femur** | 280.00 | 263.00 | 85.00 | 77.00 |  | 47.80 | 44.50 |  | 44.30 | 44.00 |  | 76.20 | 75.50 |  | 93.00 | 90.00 |  |
| **Mean** | 282.50 | 261.50 | 82.50 | 76.00 | 8.91 | 47.55 | 44.75 | 30.91 | 44.65 | 44.00 | 16.26 | 76.93 | 76.08 | 7.44 | 95.18 | 90.00 | 7.13 |
| **R.Tibia** | 480.00 | 471.00 |  |  |  | 73.60 | 74.20 |  | 127.95 | 124.50 |  | 68.05 | 64.10 |  | 58.05 | 56.00 |  |
| **L. Tibia** | 490.00 | 470.00 |  |  |  | 75.40 | 74.70 |  | 129.10 | 126.00 |  | 68.75 | 62.20 |  | 57.50 | 56.50 |  |
| **Mean** | 485.00 | 470.50 |  |  | 5.56 | 74.50 | 74.45 | 6.00 | 128.53 | 125.25 | 13.92 | 68.40 | 63.15 | 10.69 | 57.78 | 56.25 | 6.20 |
| **R. Fibula** | 484.00 | 472.00 |  |  |  | 21.40 | 18.00 |  | 44.40 | 43.00 |  |  |  |  |  |  |  |
| **L. Fibula** | 485.00 | 472.00 |  |  |  | 19.90 | 18.00 |  | 40.90 | 38.50 |  |  |  |  |  |  |  |
| **Mean** | 484.50 | 472.00 |  |  | 2.58 | 20.65 | 18.00 | 15.77 | 42.65 | 40.75 | 7.45 |  |  |  |  |  |  |
| **L. Humerus** | 316.00 | 310.00 |  |  |  | 28.20 | 25.00 |  | 16.50 | 15.50 |  | 27.20 | 26.30 |  | 23.00 | 20.30 |  |
| **R. Humerus** | 365.00 | 350.00 |  |  |  | 29.00 | 25.00 |  | 17.50 | 15.50 |  | 29.00 | 26.50 |  | 22.70 | 20.00 |  |
| **Mean** | 365.83 | 352.00 |  |  | 3.78 | 28.60 | 25.00 | 8.28 | 17.00 | 15.50 | 14.84 | 28.10 | 26.40 | 6.01 | 22.85 | 20.15 | 29.18 |
| **L. Ulna** | 109.65 | 105.40 |  |  |  | 23.20 | 20.90 |  | 22.00 | 20.40 |  | 12.65 | 11.60 |  | 24.10 | 22.00 |  |
| **R. Ulna** | 109.65 | 105.00 |  |  |  | 22.10 | 20.00 |  | 22.65 | 21.40 |  | 11.80 | 11.50 |  | 23.45 | 23.00 |  |
| **Mean** | 109.65 | 105.00 |  |  | 4.24 | 22.10 | 20.00 | 9.50 | 22.65 | 21.40 | 9.50 | 11.80 | 11.50 | 2.54 | 23.45 | 23.00 | 1.92 |
| **L. Radius** | 100.70 | 98.00 |  |  |  | 13.50 | 13.10 |  | 11.05 | 10.70 |  | 11.40 | 10.20 |  | 19.00 | 17.50 |  |
| **R. Radius** | 104.00 | 100.00 |  |  |  | 13.50 | 13.00 |  | 10.80 | 10.10 |  | 11.10 | 10.10 |  | 18.65 | 17.50 |  |
| **Mean** | 102.35 | 99.00 |  |  | 3.27 | 13.50 | 13.05 | 3.33 | 10.93 | 10.40 | 3.33 | 11.25 | 10.15 | 9.78 | 18.83 | 17.50 | 7.06 |

| ***Struthio camelus*** | | | | | | | | | | | | | | | | | |
| --- | --- | --- | --- | --- | --- | --- | --- | --- | --- | --- | --- | --- | --- | --- | --- | --- | --- |
| **OUVC 9424** | **Length** | | | |  |  | **Proximal** | | | |  |  | **Distal** | | | |  |
| **Element** | **Condyle-Trochanter** | **w/o cart** | **Trochanter-Head** | **w/o**  **Cart.** | **Percent Change** | **Medio**  **lateral** | **w/o Cart** | **Percent Change** | **Cranio**  **caudal** | **w/o Cart** | **Percent Change** | **Medio**  **lateral** | **w/o Cart** | **Percent Change** | **Cranio**  **caudal** | **w/o Cart** | **Percent Change** |
| **R. Femur** | 268.00 | 257.00 | 72.00 | 70.00 |  | 43.90 | 34.30 |  | 42.90 | 37.50 |  | 75.70 | 73.05 |  | 81.25 | 77.50 |  |
| **L. Femur** | 264.00 | 257.00 | 71.25 | 70.00 |  | 45.00 | 34.00 |  | 43.80 | 38.00 |  | 75.40 | 73.00 |  | 81.40 | 77.30 |  |
| **Mean** | 266.00 | 257.00 | 71.63 | 70.00 | 4.89 | 44.45 | 34.15 | 8.28 | 43.35 | 37.75 | 4.90 | 75.55 | 73.03 | 2.81 | 81.33 | 77.40 | 4.88 |
| **R.Tibia** | 468.00 | 450.00 |  |  |  | 72.20 | 70.00 |  | 114.20 | 104.40 |  | 62.70 | 59.00 |  | 54.10 | 51.00 |  |
| **L. Tibia** | 465.00 | 450.00 |  |  |  | 74.50 | 70.50 |  | 120.95 | 104.50 |  | 61.90 | 59.10 |  | 54.25 | 51.00 |  |
| **Mean** | 466.50 | 450.00 |  |  | 1.40 | 73.35 | 70.25 | 8.28 | 117.58 | 104.45 | 4.90 | 62.30 | 59.05 | 2.81 | 54.18 | 51.00 | 4.88 |
| **R. Fibula** |  |  |  |  |  | 22.50 | 20.20 |  | 40.40 | 39.00 |  |  |  |  |  |  |  |
| **L. Fibula** |  |  |  |  |  | 23.10 | 20.20 |  | 40.50 | 39.20 |  |  |  |  |  |  |  |
| **Mean** |  |  |  |  |  | 22.80 | 20.20 | 12.84 | 40.45 | 39.10 | 4.83 |  |  |  |  |  |  |
| **L. Humerus** | 320.00 | 308.00 |  |  |  | 32.40 | 28.40 |  | 18.50 | 13.90 |  | 31.50 | 23.90 |  | 18.60 | 14.85 |  |
| **R. Humerus** | 323.00 | 308.00 |  |  |  | 32.00 | 28.40 |  | 18.60 | 14.20 |  | 24.50 | 25.30 |  | 16.60 | 15.60 |  |
| **Mean** | 321.50 | 308.00 |  |  | 1.73 | 32.20 | 28.40 | 13.25 | 18.55 | 14.05 | 4.24 | 28.00 | 24.60 | 7.72 | 17.60 | 15.23 | 4.85 |

| ***Struthio camelus*** | | | | | | | | | | | | | | | | | |
| --- | --- | --- | --- | --- | --- | --- | --- | --- | --- | --- | --- | --- | --- | --- | --- | --- | --- |
| **OUVC 9425** | **Length** | | | |  |  | **Proximal** | | | |  |  | **Distal** | | | |  |
| **Element** | **Condyle-Trochanter** | **w/o cart** | **Trochanter-Head** | **w/o**  **Cart.** | **Percent Change** | **Medio**  **lateral** | **w/o Cart** | **Percent Change** | **Cranio**  **caudal** | **w/o Cart** | **Percent Change** | **Medio**  **lateral** | **w/o Cart** | **Percent Change** | **Cranio**  **caudal** | **w/o Cart** | **Percent Change** |
| **R. Femur** | 303.00 | 287.00 | 79.55 | 73.40 |  | 42.30 | 32.50 |  | 47.90 | 40.30 |  | 80.90 | 77.30 |  | 88.70 | 85.10 |  |
| **L. Femur** |  |  |  |  |  |  |  |  |  |  |  |  |  |  |  |  |  |
| **Mean** | 303.00 | 287.00 | 79.55 | 73.40 | 5.52 | 42.30 | 32.50 | 11.58 | 47.90 | 40.30 | 3.35 | 80.90 | 77.30 | 3.61 | 88.70 | 85.10 | 4.85 |
| **R.Tibia** | 505.00 | 484.00 |  |  |  | 79.35 | 24.50 |  | 125.30 | 110.50 |  | 61.00 | 59.50 |  | 58.50 | 55.20 |  |
| **L. Tibia** | 510.00 | 482.00 |  |  |  | 79.30 | 74.60 |  | 127.20 | 110.50 |  | 61.85 | 60.00 |  | 69.20 | 56.20 |  |
| **Mean** | 507.50 | 483.00 |  |  | 2.67 | 79.33 | 49.55 | 2.33 | 126.25 | 110.50 | 1.68 | 61.43 | 59.75 | 2.93 | 63.85 | 55.70 | 3.89 |
| **R. Fibula** |  |  |  |  |  | 24.50 | 18.50 |  | 43.20 | 39.50 |  |  |  |  |  |  |  |
| **L. Fibula** |  |  |  |  |  | 23.50 | 17.80 |  | 42.70 | 37.50 |  |  |  |  |  |  |  |
| **Mean** |  |  |  |  |  | 24.00 | 18.15 | 14.97 | 42.95 | 38.50 | 4.35 |  |  |  |  |  |  |
| **L. Humerus** | 343.00 | 325.00 |  |  |  | 28.50 | 28.00 |  | 16.80 | 13.00 |  | 27.80 | 21.00 |  | 21.80 | 17.00 |  |
| **R. Humerus** | 337.00 | 329.00 |  |  |  | 28.00 | 25.20 |  | 17.40 | 15.05 |  | 27.00 | 23.10 |  | 21.40 | 19.00 |  |
| **Mean** | 340.00 | 327.00 |  |  | 3.46 | 28.25 | 26.60 | 13.97 | 17.10 | 14.03 | 4.91 | 27.40 | 22.05 | 7.72 | 21.60 | 18.00 | 4.85 |

| ***Struthio camelus*** | | | | | | | | | | | | | | | | | |
| --- | --- | --- | --- | --- | --- | --- | --- | --- | --- | --- | --- | --- | --- | --- | --- | --- | --- |
| **OUVC 9426** | **Length** | | | | | **Proximal** | | | | | | **Distal** | | | | | |
| **Element** | **Condyle-Trochanter** | **w/o cart** | **Trochanter-Head** | **w/o**  **Cart.** | **Percent Change** | **Medio**  **lateral** | **w/o Cart** | **Percent Change** | **Cranio**  **caudal** | **w/o Cart** | **Percent Change** | **Medio**  **lateral** | **w/o Cart** | **Percent Change** | **Cranio**  **caudal** | **w/o Cart** | **Percent Change** |
| **R. Femur** | 280.00 | 267.00 | 93.50 | 76.00 |  | 46.40 | 32.60 |  | 46.60 | 37.70 |  | 80.85 | 77.00 |  | 87.40 | 83.00 |  |
| **L. Femur** | 288.00 | 265.00 | 81.10 | 77.00 |  | 47.10 | 33.20 |  | 46.80 | 29.30 |  | 82.50 | 74.15 |  | 88.60 | 84.00 |  |
| **Mean** | 284.00 | 266.00 | 87.30 | 76.50 | 3.22 | 46.75 | 32.90 | 9.54 | 46.70 | 33.50 | 9.29 | 81.68 | 75.58 | 2.67 | 88.00 | 83.50 | 3.57 |
| **R.Tibia** | 502.00 | 490.00 |  |  |  | 78.70 | 77.60 |  | 129.30 | 123.00 |  | 64.80 | 63.30 |  | 61.45 | 59.70 |  |
| **L. Tibia** | 522.00 | 497.00 |  |  |  | 78.80 | 70.20 |  | 128.65 | 109.70 |  | 64.50 | 63.10 |  | 58.80 | 55.65 |  |
| **Mean** | 512.00 | 493.50 |  |  | 1.59 | 78.75 | 73.90 | 1.24 | 128.98 | 116.35 | 3.34 | 64.65 | 63.20 | 2.11 | 60.13 | 57.68 | 3.80 |
| **R. Fibula** |  |  |  |  |  | 21.30 | 19.40 |  | 44.40 | 39.00 |  |  |  |  |  |  |  |
| **L. Fibula** |  |  |  |  |  | 23.00 | 19.35 |  | 40.50 | 38.50 |  |  |  |  |  |  |  |
| **Mean** |  |  |  |  |  | 22.15 | 19.38 | 19.69 | 42.45 | 38.75 | 4.41 |  |  |  |  |  |  |
| **L. Humerus** | 350.00 | 338.00 |  |  |  | 30.40 | 27.50 |  | 20.50 | 16.10 |  | 29.00 | 20.50 |  | 23.50 | 18.20 |  |
| **R. Humerus** | 334.00 | 325.00 |  |  |  | 28.80 | 28.00 |  | 17.20 | 13.40 |  | 26.30 | 20.80 |  | 19.50 | 16.45 |  |
| **Mean** | 342.00 | 331.50 |  | ! | 0.85 | 29.60 | 27.75 | 6.07 | 18.85 | 14.75 | 2.07 | 27.65 | 20.65 | 9.46 | 21.50 | 17.33 | 6.54 |

| ***Struthio camelus*** | | | | | | | | | | | | | | | | | |
| --- | --- | --- | --- | --- | --- | --- | --- | --- | --- | --- | --- | --- | --- | --- | --- | --- | --- |
| **OUVC 9427** | **Length** | | | | | **Proximal** | | | | | | **Distal** | | | | | |
| **Element** | **Condyle-Trochanter** | **w/o cart** | **Trochanter-Head** | **w/o**  **Cart.** | **Percent Change** | **Medio**  **lateral** | **w/o Cart** | **Percent Change** | **Cranio**  **caudal** | **w/o Cart** | **Percent Change** | **Medio**  **lateral** | **w/o Cart** | **Percent Change** | **Cranio**  **caudal** | **w/o Cart** | **Percent Change** |
| **R. Femur** | 300.00 | 283.00 | 93.70 | 86.60 |  | 49.00 | 45.05 |  | 50.00 | 47.40 |  | 84.00 | 82.60 |  | 91.35 | 87.40 |  |
| **L. Femur** | 304.00 | 295.00 | 91.00 | 85.50 |  | 48.20 | 44.10 |  | 47.90 | 45.70 |  | 85.25 | 81.90 |  | 92.00 | 87.00 |  |
| **Mean** | 302.00 | 289.00 | 92.35 | 86.05 | 6.05 | 48.60 | 44.58 | 4.28 | 48.95 | 46.55 | 2.46 | 84.63 | 82.25 | 4.71 | 91.68 | 87.20 | 1.62 |
| **R.Tibia** | 535.00 | 527.00 |  |  |  | 85.90 | 82.50 |  | 132.50 | 128.40 |  | 68.00 | 65.40 |  | 63.00 | 59.35 |  |
| **L. Tibia** | 535.00 | 528.00 |  |  |  | 84.00 | 81.60 |  | 132.00 | 129.70 |  | 67.00 | 65.25 |  | 63.80 | 61.10 |  |
| **Mean** | 535.00 | 527.50 |  |  | 1.89 | 84.95 | 82.05 | 2.06 | 132.25 | 129.05 | 9.5 | 67.50 | 65.33 | 4.41 | 63.40 | 60.23 | 5.35 |
| **R. Fibula** |  |  |  |  |  | 20.50 | 20.50 |  | 41.50 | 40.70 |  |  |  |  |  |  |  |
| **L. Fibula** |  |  |  |  |  | 27.00 | 20.90 |  | 43.40 | 40.10 |  |  |  |  |  |  |  |
| **Mean** |  |  |  |  |  | 23.75 | 20.70 | 13.26 | 42.45 | 40.40 | 21.10 |  |  |  |  |  |  |
| **L. Humerus** | 344.00 | 341.00 |  |  |  | 32.45 | 28.00 |  | 16.90 | 14.85 |  | 26.50 | 26.00 |  | 21.65 | 20.70 |  |
| **R. Humerus** | 349.00 | 340.00 |  |  |  | 32.10 | 28.00 |  | 16.10 | 14.80 |  | 27.80 | 26.00 |  | 22.30 | 20.80 |  |
| **Mean** | 346.50 | 340.50 |  |  | 4.37 | 32.28 | 28.00 | 7.93 | 16.50 | 14.83 | 14.00 | 27.15 | 26.00 | 4.88 | 21.98 | 20.75 | 12.90 |

| ***Struthio camelus*** | | | | | | | | | | | | | | | | | |
| --- | --- | --- | --- | --- | --- | --- | --- | --- | --- | --- | --- | --- | --- | --- | --- | --- | --- |
| **OUVC 9428** | **Length** | | | | | **Proximal** | | | | | | **Distal** | | | | | |
| **Element** | **Condyle-Trochanter** | **w/o cart** | **Trochanter-Head** | **w/o**  **Cart.** | **Percent Change** | **Medio**  **lateral** | **w/o Cart** | **Percent Change** | **Cranio**  **caudal** | **w/o Cart** | **Percent Change** | **Medio**  **lateral** | **w/o Cart** | **Percent Change** | **Cranio**  **caudal** | **w/o Cart** | **Percent Change** |
| **R. Femur** | 268.00 | 255.00 | 82.10 | 78.45 |  | 46.10 | 44.20 |  | 47.50 | 44.50 |  | 78.10 | 77.40 |  | 81.65 | 80.30 |  |
| **L. Femur** | 265.00 | 255.00 | 81.60 | 78.00 |  | 47.00 | 44.50 |  | 47.40 | 45.80 |  | 81.40 | 80.00 |  | 87.20 | 86.60 |  |
| **Mean** | 266.50 | 255.00 | 81.85 | 78.23 | 7.76 | 46.55 | 44.35 | 29.63 | 47.45 | 45.15 | 28.27 | 79.75 | 78.70 | 7.47 | 84.43 | 83.45 | 5.11 |
| **R.Tibia** | 443.00 | 437.00 |  |  |  | 75.90 | 73.30 |  | 119.90 | 117.50 |  | 68.20 | 60.40 |  | 57.20 | 55.40 |  |
| **L. Tibia** | 449.00 | 446.00 |  |  |  | 78.95 | 75.70 |  | 129.65 | 119.70 |  | 62.10 | 61.00 |  | 57.00 | 55.00 |  |
| **Mean** | 446.00 | 441.50 |  |  | 3.61 | 77.43 | 74.50 | 6.16 | 124.78 | 118.60 | 9.79 | 65.15 | 60.70 | 2.24 | 57.10 | 55.20 | 4.07 |
| **R. Fibula** |  |  |  |  |  | 23.50 | 19.20 |  | 40.50 | 37.30 |  |  |  |  |  |  |  |
| **L. Fibula** |  |  |  |  |  | 23.30 | 19.00 |  | 41.40 | 37.50 |  |  |  |  |  |  |  |
| **Mean** |  |  |  |  |  | 23.40 | 19.10 | 1.53 | 40.95 | 37.40 | 8.72 |  |  |  |  |  |  |
| **L. Humerus** | 328.00 | 320.00 |  |  |  | 30.10 | 27.50 |  | 17.00 | 14.80 |  | 26.50 | 23.90 |  | 23.20 | 20.30 |  |
| **R. Humerus** | 328.00 | 320.00 |  |  |  | 30.00 | 27.70 |  | 17.50 | 14.55 |  | 27.10 | 24.80 |  | 22.10 | 19.60 |  |
| **Mean** | 328.00 | 320.00 |  |  | 3.07 | 30.05 | 27.60 | 6.25 | 17.25 | 14.68 | 21.75 | 26.80 | 24.35 | 25.32 | 22.65 | 19.95 | 19.42 |

| ***Struthio camelus*** | | | | | | | | | | | | | | | | | |
| --- | --- | --- | --- | --- | --- | --- | --- | --- | --- | --- | --- | --- | --- | --- | --- | --- | --- |
| **OUVC 9429** | **Length** | | | | | **Proximal** | | | | | | **Distal** | | | | | |
| **Element** | **Condyle-Trochanter** | **w/o cart** | **Trochanter-Head** | **w/o**  **Cart.** | **Percent Change** | **Medio**  **lateral** | **w/o Cart** | **Percent Change** | **Cranio**  **caudal** | **w/o Cart** | **Percent Change** | **Medio**  **lateral** | **w/o Cart** | **Percent Change** | **Cranio**  **caudal** | **w/o Cart** | **Percent Change** |
| **R. Femur** | 293.00 | 270.00 | 85.00 | 85.00 |  | 48.75 | 46.10 |  | 47.55 | 46.10 |  | 83.75 | 81.70 |  | 88.10 | 85.70 |  |
| **L. Femur** | 295.00 | 278.00 | 87.00 | 81.00 |  | 45.90 | 44.50 |  | 48.00 | 47.10 |  | 87.00 | 81.00 |  | 84.40 | 84.00 |  |
| **Mean** | 294.00 | 274.00 | 86.00 | 83.00 | 2.73 | 47.33 | 45.30 | 6.29 | 47.78 | 46.60 | 9.31 | 85.38 | 81.35 | 1.22 | 86.25 | 84.85 | 3.11 |
| **R.Tibia** | 510.00 | 496.00 |  |  |  | 83.30 | 81.00 |  | 130.00 | 127.70 |  | 65.50 | 65.00 |  | 64.00 | 58.75 |  |
| **L. Tibia** | 497.00 | 492.00 |  |  |  | 81.60 | 80.50 |  | 153.00 | 129.70 |  | 59.10 | 54.10 |  | 60.30 | 58.90 |  |
| **Mean** | 503.50 | 494.00 |  |  | 3.62 | 82.45 | 80.75 | 1.43 | 141.50 | 128.70 | 3.08 | 62.30 | 59.55 | 2.22 | 62.15 | 58.83 | 4.78 |
| **R. Fibula** |  |  |  |  |  | 25.00 | 22.30 |  | 44.90 | 40.65 |  |  |  |  |  |  |  |
| **L. Fibula** |  |  |  |  |  | 22.50 | 18.90 |  | 39.70 | 26.10 |  |  |  |  |  |  |  |
| **Mean** |  |  |  |  |  | 23.75 | 20.60 | 8.96 | 42.30 | 33.38 | 6.52 |  |  |  |  |  |  |
| **L. Humerus** | 367.00 | 350.00 |  |  |  | 35.40 | 32.00 |  | 20.20 | 17.20 |  | 28.35 | 26.60 |  | 21.80 | 19.25 |  |
| **R. Humerus** | 365.00 | 350.00 |  |  |  | 35.20 | 33.00 |  | 19.80 | 17.20 |  | 28.00 | 27.00 |  | 23.15 | 19.90 |  |
| **Mean** | 366.00 | 350.00 |  |  | 2.04 | 35.30 | 32.50 | 5.14 | 20.00 | 17.20 | 19.12 | 28.18 | 26.80 | 8.74 | 22.48 | 19.58 | 10.18 |

| ***Struthio camelus*** | | | | | | | | | | | | | | | | | |
| --- | --- | --- | --- | --- | --- | --- | --- | --- | --- | --- | --- | --- | --- | --- | --- | --- | --- |
| **OUVC 9430** | **Length** | | | | | **Proximal** | | | | | | **Distal** | | | | | |
| **Element** | **Condyle-Trochanter** | **w/o cart** | **Trochanter-Head** | **w/o**  **Cart.** | **Percent Change** | **Medio**  **lateral** | **w/o Cart** | **Percent Change** | **Cranio**  **caudal** | **w/o Cart** | **Percent Change** | **Medio**  **lateral** | **w/o Cart** | **Percent Change** | **Cranio**  **caudal** | **w/o Cart** | **Percent Change** |
| **R. Femur** | 310.00 | 290.00 | 93.00 | 90.50 |  | 52.40 | 45.75 |  | 50.00 | 48.70 |  | 87.50 | 83.90 |  | 92.50 | 87.00 |  |
| **L. Femur** | 310.00 | 290.00 | 93.50 | 91.50 |  | 52.50 | 47.00 |  | 48.50 | 46.50 |  | 87.10 | 84.40 |  | 91.10 | 87.70 |  |
| **Mean** | 310.00 | 290.00 | 93.25 | 91.00 | 5.79 | 52.45 | 46.38 | 23.17 | 49.25 | 47.60 | 15.87 | 87.30 | 84.15 | 4.45 | 91.80 | 87.35 | 4.06 |
| **R.Tibia** | 522.00 | 508.00 |  |  |  | 84.70 | 82.00 |  | 136.00 | 132.80 |  | 68.70 | 66.85 |  | 69.65 | 66.90 |  |
| **L. Tibia** | 525.00 | 511.00 |  |  |  | 85.00 | 83.75 |  | 138.00 | 136.60 |  | 67.65 | 65.50 |  | 62.70 | 60.30 |  |
| **Mean** | 523.50 | 509.50 |  |  | 4.83 | 84.85 | 82.88 | 37.54 | 137.00 | 134.70 | 12.48 | 68.18 | 66.18 | 2.73 | 66.18 | 63.60 | 12.76 |
| **R. Fibula** |  |  |  |  |  | 23.75 | 21.30 |  | 45.15 | 41.50 |  |  |  |  |  |  |  |
| **L. Fibula** |  |  |  |  |  | 26.00 | 21.00 |  | 43.40 | 43.20 |  |  |  |  |  |  |  |
| **Mean** |  |  |  |  |  | 24.88 | 21.15 | 24.38 | 44.28 | 42.35 | 10.36 |  |  |  |  |  |  |
| **L. Humerus** | 375.00 | 365.00 |  |  |  | 20.40 | 18.10 |  | 34.70 | 33.80 |  | 30.45 | 28.70 |  | 23.90 | 22.70 |  |
| **R. Humerus** | 376.00 | 360.00 |  |  |  | 20.40 | 17.00 |  | 34.50 | 32.00 |  | 30.40 | 27.45 |  | 23.50 | 22.40 |  |
| **Mean** | 375.50 | 362.50 |  |  | 3.82 | 20.40 | 17.55 | 5.84 | 34.60 | 32.90 | 17.98 | 30.43 | 28.08 | 19.53 | 23.70 | 22.55 | 16.67 |

| ***Struthio camelus*** | | | | | | | | | | | | | | | | | |
| --- | --- | --- | --- | --- | --- | --- | --- | --- | --- | --- | --- | --- | --- | --- | --- | --- | --- |
| **OUVC 9431** | **Length** | | | |  | **Proximal** | | | | | | **Distal** | | | | | |
| **Element** | **Condyle-Trochanter** | **w/o cart** | **Trochanter-Head** | **w/o**  **Cart.** | **Percent Change** | **Medio**  **lateral** | **w/o Cart** | **Percent Change** | **Cranio**  **caudal** | **w/o Cart** | **Percent Change** | **Medio**  **lateral** | **w/o Cart** | **Percent Change** | **Cranio**  **caudal** | **w/o Cart** | **Percent Change** |
| **R. Femur** | 260.00 | 253.00 | 71.00 | 69.00 |  | 42.50 | 38.00 |  | 43.00 | 39.40 |  | 68.40 | 67.90 |  | 78.50 | 76.10 |  |
| **L. Femur** | 260.00 | 253.00 | 71.10 | 69.00 |  | 41.00 | 40.25 |  | 43.50 | 39.05 |  | 71.10 | 69.90 |  | 79.00 | 76.50 |  |
| **Mean** | 260.00 | 253.00 | 71.05 | 69.00 | 3.15 | 41.75 | 39.13 | 23.17 | 43.25 | 39.23 | 12.92 | 69.75 | 68.90 | 3.34 | 78.75 | 76.30 | 4.83 |
| **R.Tibia** | 456.00 | 449.00 |  |  |  | 69.00 | 68.00 |  | 119.40 | 115.50 |  | 56.00 | 54.90 |  | 55.80 | 53.50 |  |
| **L. Tibia** | 510.00 | 482.00 |  |  |  | 71.00 | 70.00 |  | 121.00 | 117.50 |  | 56.80 | 55.40 |  | 58.15 | 55.00 |  |
| **Mean** | 483.00 | 465.50 |  |  | 3.54 | 70.00 | 69.00 | 4.23 | 120.20 | 116.50 | 11.16 | 56.40 | 55.15 | 5.22 | 56.98 | 54.25 | 5.86 |
| **R. Fibula** |  |  |  |  |  | 23.70 | 21.20 |  | 41.35 | 37.50 |  |  |  |  |  |  |  |
| **L. Fibula** |  |  |  |  |  | 23.20 | 21.50 |  | 40.80 | 39.30 |  |  |  |  |  |  |  |
| **Mean** |  |  |  |  |  | 23.45 | 21.35 | 11.40 | 41.08 | 38.40 | 3.34 |  |  |  |  |  |  |
| **L. Humerus** | 349.00 | 343.00 |  |  |  | 32.30 | 32.00 |  | 20.00 | 15.20 |  | 26.80 | 26.00 |  | 22.80 | 20.70 |  |
| **R. Humerus** | 337.00 | 329.00 |  |  |  | 28.00 | 25.20 |  | 17.40 | 15.05 |  | 27.00 | 23.10 |  | 21.40 | 19.00 |  |
| **Mean** | 343.00 | 336.00 |  |  | 4.20 | 30.15 | 28.60 | 11.80 | 18.70 | 15.13 | 24.26 | 26.90 | 24.55 | 12.14 | 22.10 | 19.85 | 13.49 |

| ***Struthio camelus*** | | | | | | | | | | | | | | | | | |
| --- | --- | --- | --- | --- | --- | --- | --- | --- | --- | --- | --- | --- | --- | --- | --- | --- | --- |
| **OUVC 9432** | **Length** | | | | | **Proximal** | | | | | | **Distal** | | | | | |
| **Element** | **Condyle-Trochanter** | **w/o cart** | **Trochanter-Head** | **w/o**  **Cart.** | **Percent Change** | **Medio**  **lateral** | **w/o Cart** | **Percent Change** | **Cranio**  **caudal** | **w/o Cart** | **Percent Change** | **Medio**  **lateral** | **w/o Cart** | **Percent Change** | **Cranio**  **caudal** | **w/o Cart** | **Percent Change** |
| **R. Femur** | 295.00 | 287.00 | 90.00 | 90.00 |  | 49.50 | 41.40 |  | 48.10 | 45.20 |  | 85.15 | 83.75 |  | 88.35 | 85.60 |  |
| **L. Femur** | 300.00 | 283.00 | 92.00 | 92.00 |  | 48.00 | 46.80 |  | 52.00 | 45.60 |  | 85.00 | 81.85 |  | 88.00 | 84.45 |  |
| **Mean** | 297.50 | 285.00 | 91.00 | 91.00 | 4.34 | 48.75 | 44.10 | 4.73 | 50.05 | 45.40 | 4.85 | 85.08 | 82.80 | 1.32 | 88.18 | 85.03 | 1.15 |
| **R.Tibia** | 506.00 | 492.00 |  |  |  | 80.10 | 78.60 |  | 132.50 | 128.50 |  | 65.10 | 64.15 |  | 62.25 | 59.40 |  |
| **L. Tibia** | 500.00 | 498.00 |  |  |  | 81.00 | 80.50 |  | 135.50 | 130.55 |  | 65.00 | 63.20 |  | 61.50 | 59.65 |  |
| **Mean** | 503.00 | 495.00 |  |  | 1.01 | 80.55 | 79.55 | 3.78 | 134.00 | 129.53 | 4.95 | 65.05 | 63.68 | 6.83 | 61.88 | 59.53 | 3.33 |
| **R. Fibula** |  |  |  |  |  | 29.10 | 23.25 |  | 46.15 | 42.40 |  |  |  |  |  |  |  |
| **L. Fibula** |  |  |  |  |  | 26.00 | 21.00 |  | 43.40 | 43.20 |  |  |  |  |  |  |  |
| **Mean** |  |  |  |  |  | 27.55 | 22.13 | 18.38 | 44.78 | 42.80 | 8.67 |  |  |  |  |  |  |
| **L. Humerus** | 352.00 | 347.00 |  |  |  | 33.30 | 30.10 |  | 17.70 | 16.50 |  | 30.20 | 26.40 |  | 22.80 | 21.70 |  |
| **R. Humerus** | 353.00 | 352.00 |  |  |  | 31.75 | 31.00 |  | 18.55 | 19.00 |  | 29.00 | 27.20 |  | 23.10 | 21.20 |  |
| **Mean** | 352.50 | 349.50 |  |  | 2.44 | 32.53 | 30.55 | 8.15 | 18.13 | 17.75 | 14.93 | 29.60 | 26.80 | 9.14 | 22.95 | 21.45 | 11.92 |

| ***Struthio camelus*** | | | | | | | | | | | | | | | | | |
| --- | --- | --- | --- | --- | --- | --- | --- | --- | --- | --- | --- | --- | --- | --- | --- | --- | --- |
| **OUVC 9433** | **Length** | | | | | **Proximal** | | | | | | **Distal** | | | | | |
| **Element** | **Condyle-Trochanter** | **w/o cart** | **Trochanter-Head** | **w/o**  **Cart.** | **Percent Change** | **Medio**  **lateral** | **w/o Cart** | **Percent Change** | **Cranio**  **caudal** | **w/o Cart** | **Percent Change** | **Medio**  **lateral** | **w/o Cart** | **Percent Change** | **Cranio**  **caudal** | **w/o Cart** | **Percent Change** |
| **R. Femur** | 287.00 | 277.00 | 90.70 | 80.00 |  | 50.70 | 45.10 |  | 46.05 | 46.05 |  | 82.20 | 82.00 |  | 88.50 | 86.60 |  |
| **L. Femur** | 278.00 | 275.00 | 85.30 | 85.00 |  | 49.90 | 43.30 |  | 47.30 | 45.80 |  | 81.40 | 80.00 |  | 87.20 | 86.60 |  |
| **Mean** | 282.50 | 276.00 | 88.00 | 82.50 | 5.26 | 50.30 | 44.20 | 13.86 | 46.68 | 45.93 | 9.21 | 81.80 | 81.00 | 3.43 | 87.85 | 86.60 | 3.93 |
| **R.Tibia** | 502.00 | 490.00 |  |  |  | 78.70 | 77.60 |  | 129.30 | 123.00 |  | 64.80 | 63.30 |  | 61.45 | 59.70 |  |
| **L. Tibia** | 502.00 | 500.00 |  |  |  | 81.00 | 80.00 |  | 133.70 | 126.30 |  | 64.60 | 62.40 |  | 62.00 | 58.70 |  |
| **Mean** | 502.00 | 495.00 |  |  | 2.84 | 79.85 | 78.80 | 5.80 | 131.50 | 124.65 | 6.63 | 64.70 | 62.85 | 4.43 | 61.73 | 59.20 | 5.15 |
| **R. Fibula** |  |  |  |  |  | 31.30 | 28.00 |  | 44.40 | 41.00 |  |  |  |  |  |  |  |
| **L. Fibula** |  |  |  |  |  | 28.40 | 28.00 |  | 42.00 | 41.00 |  |  |  |  |  |  |  |
| **Mean** |  |  |  |  |  | 29.85 | 28.00 | 14.27 | 43.20 | 41.00 | 7.44 |  |  |  |  |  |  |
| **L. Humerus** | 349.00 | 343.00 |  |  |  | 32.30 | 32.00 |  | 20.00 | 15.20 |  | 26.80 | 26.00 |  | 22.80 | 20.70 |  |
| **R. Humerus** | 349.00 | 340.00 |  |  |  | 32.00 | 27.90 |  | 16.80 | 15.80 |  | 30.80 | 25.70 |  | 22.50 | 18.20 |  |
| **Mean** | 349.00 | 341.50 |  |  | 2.80 | 32.15 | 29.95 | 8.84 | 18.40 | 15.50 | 14.05 | 28.80 | 25.85 | 10.29 | 22.65 | 19.45 | 13.06 |

| ***Coturnix japonica*** | | | | | | | | | | | | | | | | | |
| --- | --- | --- | --- | --- | --- | --- | --- | --- | --- | --- | --- | --- | --- | --- | --- | --- | --- |
| **OUVC 9416** | **Length** | | | | | **Proximal** | | | | | | **Distal** | | | | | |
| **Element** | **Condyle-Trochanter** | **w/o cart** | **Trochanter-Head** | **w/o**  **Cart.** | **Percent Change** | **Medio**  **lateral** | **w/o Cart** | **Percent Change** | **Cranio**  **caudal** | **w/o Cart** | **Percent Change** | **Medio**  **lateral** | **w/o Cart** | **Percent Change** | **Cranio**  **caudal** | **w/o Cart** | **Percent Change** |
| **Femur** | 35.65 | 35.50 | 6.80 | 6.70 | 0.59 | 2.73 | 2.55 | 6.71 | 2.63 | 2.50 | 5.06 | 5.77 | 5.60 | 2.89 | 4.27 | 4.00 | 6.25 |
| **Tibia** | 42.82 | 42.30 |  |  | 1.21 | 4.74 | 4.45 | 6.15 | 6.62 | 6.48 | 2.14 | 4.09 | 3.93 | 4.07 | 3.11 | 3.00 | 3.49 |
| **Fibula** |  |  |  |  |  | 1.39 | 1.30 | 6.59 | 3.31 | 3.12 | 5.79 |  |  |  |  |  |  |
| **Humerus** | 32.56 | 32.39 |  |  | 0.51 | 8.47 | 7.95 | 6.10 | 6.78 | 6.37 | 6.03 | 3.74 | 3.23 | 13.81 | 2.30 | 2.25 | 2.17 |
| **Radius** | 25.18 | 24.52 |  |  | 2.62 | 2.30 | 2.30 | 0.00 | 2.06 | 1.90 | 7.69 | 25.18 | 24.52 | 2.62 | 1.67 | 1.65 | 1.00 |
| **Ulna** | 27.08 | 27.03 |  |  | 0.15 | 4.97 | 4.75 | 4.36 | 5.23 | 5.10 | 2.55 | 2.33 | 2.20 | 5.38 | 3.04 | 3.00 | 1.37 |

| ***Coturnix japonica*** | | | | | | | | | | | | | | | | | |
| --- | --- | --- | --- | --- | --- | --- | --- | --- | --- | --- | --- | --- | --- | --- | --- | --- | --- |
| **OUVC 9417** | **Length** | | | | | **Proximal** | | | | | | **Distal** | | | | | |
| **Element** | **Condyle-Trochanter** | **w/o cart** | **Trochanter-Head** | **w/o**  **Cart.** | **Percent Change** | **Medio**  **lateral** | **w/o Cart** | **Percent Change** | **Cranio**  **caudal** | **w/o Cart** | **Percent Change** | **Medio**  **lateral** | **w/o Cart** | **Percent Change** | **Cranio**  **caudal** | **w/o Cart** | **Percent Change** |
| **Femur** | 38.46 | 38.13 | 6.95 | 6.80 | 1.06 | 2.50 | 2.43 | 3.00 | 2.90 | 2.65 | 8.62 | 5.79 | 5.58 | 3.74 | 4.43 | 4.43 | 0.08 |
| **Tibia** | 46.40 | 45.90 |  |  | 1.08 | 4.85 | 4.80 | 1.03 | 6.70 | 6.70 | 0.00 | 4.71 | 4.50 | 4.42 | 4.77 | 4.68 | 1.92 |
| **Fibula** |  |  |  |  |  | 1.35 | 1.30 | 3.70 | 3.80 | 3.60 | 5.26 |  |  |  |  |  |  |
| **Humerus** | 34.98 | 34.70 |  |  | 0.81 | 8.34 | 8.18 | 2.00 | 6.38 | 6.07 | 4.84 | 3.53 | 3.10 | 12.06 | 2.47 | 2.35 | 4.73 |
| **Radius** | 27.35 | 27.30 |  |  | 0.18 | 2.58 | 2.58 | 0.00 | 2.08 | 2.00 | 4.00 | 2.88 | 2.73 | 5.22 | 1.65 | 1.57 | 5.05 |
| **Ulna** | 30.33 | 29.85 |  |  | 1.57 | 3.43 | 3.29 | 4.13 | 3.03 | 2.99 | 1.57 | 2.35 | 2.25 | 4.26 | 3.20 | 3.15 | 1.56 |

| ***Coturnix japonica*** | | | | | | | | | | | | | | | | | |
| --- | --- | --- | --- | --- | --- | --- | --- | --- | --- | --- | --- | --- | --- | --- | --- | --- | --- |
| **OUVC 9418** | **Length** | | | | | **Proximal** | | | | | | **Distal** | | | | | |
| **Element** | **Condyle-Trochanter** | **w/o cart** | **Trochanter-Head** | **w/o**  **Cart.** | **Percent Change** | **Medio**  **lateral** | **w/o Cart** | **Percent Change** | **Cranio**  **caudal** | **w/o Cart** | **Percent Change** | **Medio**  **lateral** | **w/o Cart** | **Percent Change** | **Cranio**  **caudal** | **w/o Cart** | **Percent Change** |
| **Femur** | 38.37 | 38.08 | 7.03 | 6.70 | 1.35 | 2.57 | 2.50 | 2.72 | 2.80 | 2.68 | 8.62 | 5.80 | 5.60 | 3.45 | 4.33 | 4.23 | 2.31 |
| **Tibia** | 44.40 | 44.30 |  |  | 0.23 | 4.72 | 4.61 | 2.31 | 6.80 | 6.80 | 0.00 | 3.63 | 3.40 | 6.21 | 4.61 | 4.46 | 3.26 |
| **Fibula** |  |  |  |  |  | 1.30 | 1.20 | 7.69 | 3.28 | 3.19 | 2.77 |  |  |  |  |  |  |
| **Humerus** | 34.35 | 34.23 |  |  | 0.35 | 4.94 | 4.00 | 19.06 | 6.75 | 6.20 | 8.15 | 4.80 | 4.49 | 6.46 | 3.50 | 3.15 | 10.00 |
| **Radius** | 26.48 | 25.35 |  |  | 4.27 | 2.45 | 2.40 | 0  2.04 | 2.05 | 1.95 | 4.88 | 2.64 | 2.50 | 5.37 | 1.60 | 1.58 | 1.25 |
| **Ulna** | 28.67 | 28.63 |  |  | 0.14 | 4.06 | 3.92 | 3.47 | 5.50 | 5.45 | 1.57 | 2.35 | 2.27 | 4.26 | 3.03 | 2.99 | 1.35 |

| ***Gallus gallus*** | | | | | | | | | | | | | | | | | |
| --- | --- | --- | --- | --- | --- | --- | --- | --- | --- | --- | --- | --- | --- | --- | --- | --- | --- |
| **OUVC 9419** | **Length** | | | | | **Proximal** | | | | | | **Distal** | | | | | |
| **Element** | **Condyle-Trochanter** | **w/o cart** | **Trochanter-Head** | **w/o**  **Cart.** | **Percent Change** | **Medio**  **lateral** | **w/o Cart** | **Percent Change** | **Cranio**  **caudal** | **w/o Cart** | **Percent Change** | **Medio**  **lateral** | **w/o Cart** | **Percent Change** | **Cranio**  **caudal** | **w/o Cart** | **Percent Change** |
| **Femur** | 112.10 | 106.37 | 22.62 | 18.60 | 7.24 | 10.02 | 7.23 | 27.87 | 11.09 | 9.13 | 17.73 | 24.11 | 20.50 | 14.97 | 20.14 | 17.95 | 10.88 |
| **Tibia** | 150.50 | 145.95 |  |  | 3.02 | 21.55 | 18.40 | 14.62 | 28.31 | 26.36 | 6.89 | 18.00 | 17.58 | 2.36 | 18.38 | 17.70 | 3.67 |
| **Fibula** |  |  |  |  |  | 4.80 | 4.18 | 13.02 | 12.14 | 10.63 | 12.42 |  |  |  |  |  |  |
| **Humerus** | 101.00 | 99.50 |  |  | 1.49 | 19.95 | 17.10 | 14.29 | 10.10 | 9.91 | 1.88 | 15.50 | 14.00 | 9.68 | 13.50 | 10.30 | 23.70 |
| **Radius** | 93.00 | 90.00 |  |  | 3.23 | 7.91 | 6.80 | 14.03 | 7.43 | 6.66 | 10.36 | 7.40 | 7.00 | 5.41 | 5.27 | 5.00 | 5.12 |
| **Ulna** | 101.00 | 98.90 |  |  | 2.08 | 7.34 | 6.33 | 13.76 | 8.85 | 8.25 | 6.78 | 9.80 | 8.75 | 10.71 | 11.20 | 9.50 | 15.18 |

| ***Gallus gallus*** | | | | | | | | | | | | | | | | | |
| --- | --- | --- | --- | --- | --- | --- | --- | --- | --- | --- | --- | --- | --- | --- | --- | --- | --- |
| **OUVC 9420** | **Length** | | | | | **Proximal** | | | | | | **Distal** | | | | | |
| **Element** | **Condyle-Trochanter** | **w/o cart** | **Trochanter-Head** | **w/o**  **Cart.** | **Percent Change** | **Medio**  **lateral** | **w/o Cart** | **Percent Change** | **Cranio**  **caudal** | **w/o Cart** | **Percent Change** | **Medio**  **lateral** | **w/o Cart** | **Percent Change** | **Cranio**  **caudal** | **w/o Cart** | **Percent Change** |
| **Femur** | 99.66 | 90.76 | 24.58 | 18.77 | 11.84 | 10.06 | 6.70 | 33.39 | 10.17 | 7.93 | 22.05 | 21.48 | 18.29 | 14.82 | 15.04 | 14.58 | 3.10 |
| **Tibia** | 138.47 | 127.52 |  |  | 7.91 | 18.84 | 15.97 | 15.26 | 26.37 | 25.15 | 4.61 | 18.59 | 17.48 | 5.96 | 18.95 | 13.13 | 30.69 |
| **Fibula** |  |  |  |  |  | 3.68 | 3.50 | 4.76 | 12.28 | 10.38 | 15.48 |  |  |  |  |  |  |
| **Humerus** | 93.98 | 87.92 |  |  | 6.45 | 17.65 | 15.50 | 12.18 | 10.02 | 8.51 | 15.06 | 15.53 | 13.00 | 16.31 | 11.03 | 6.70 | 39.23 |
| **Radius** | 81.31 | 77.60 |  |  | 4.56 | 7.34 | 5.79 | 21.11 | 6.43 | 5.28 | 18.01 | 9.94 | 8.50 | 14.50 | 4.64 | 3.35 | 27.83 |
| **Ulna** | 89.22 | 80.91 |  |  | 9.31 | 14.93 | 10.88 | 27.18 | 10.55 | 10.50 | 0.47 | 9.76 | 8.50 | 12.89 | 10.00 | 9.00 | 10.00 |

| ***Aix sponsa*** | | | | | | | | | | | | | | | | | |
| --- | --- | --- | --- | --- | --- | --- | --- | --- | --- | --- | --- | --- | --- | --- | --- | --- | --- |
| **OUVC 9421** | **Length** | | | | | **Proximal** | | | | | | **Distal** | | | | | |
| **Element** | **Condyle-Trochanter** | **w/o cart** | **Trochanter-Head** | **w/o**  **Cart.** | **Percent Change** | **Medio**  **lateral** | **w/o Cart** | **Percent Change** | **Cranio**  **caudal** | **w/o Cart** | **Percent Change** | **Medio**  **lateral** | **w/o Cart** | **Percent Change** | **Cranio**  **caudal** | **w/o Cart** | **Percent Change** |
| **Femur** | 39.83 | 39.60 | 9.08 | 8.85 | 0.92 | 3.51 | 3.45 | 1.66 | 5.05 | 4.83 | 4.46 | 9.63 | 9.50 | 1.30 | 6.81 | 6.70 | 1.59 |
| **Tibia** | 60.27 | 60.20 |  |  | 0.11 | 7.49 | 7.25 | 3.23 | 10.75 | 10.43 | 3.02 | 7.32 | 7.25 | 0.91 | 7.51 | 7.40 | 1.44 |
| **Fibula** |  |  |  |  |  | 2.10 | 1.95 | 7.14 | 4.55 | 4.43 | 2.75 |  |  |  |  |  |  |
| **Humerus** | 50.88 | 50.85 |  |  | 0.07 | 4.65 | 4.58 | 1.70 | 8.35 | 7.23 | 13.41 | 7.01 | 6.80 | 3.03 | 5.06 | 4.99 | 1.48 |
| **Radius** | 51.38 | 51.22 |  |  | 0.31 | 4.43 | 4.13 | 6.78 | 4.58 | 4.45 | 2.73 | 5.43 | 5.40 | 0.46 | 2.53 | 2.36 | 6.60  0.00 |
| **Ulna** | 55.63 | 55.63 |  |  | 0.00 | 7.90 | 7.45 | 5.70 | 7.27 | 7.20 | 0.92 | 4.83 | 4.79 | 0.69 | 7.27 | 7.20 | 0.92 |
